# Immersive Nature-Experiences as Health Promotion Interventions for Healthy, Vulnerable, and Sick Populations? A Systematic Review and Appraisal of Controlled Studies

**DOI:** 10.3389/fpsyg.2019.00943

**Published:** 2019-05-03

**Authors:** Lærke Mygind, Eva Kjeldsted, Rikke Dalgaard Hartmeyer, Erik Mygind, Mads Bølling, Peter Bentsen

**Affiliations:** ^1^Steno Diabetes Center Copenhagen, Gentofte, Denmark; ^2^Department of Geosciences and Natural Resource Management, University of Copenhagen, Copenhagen, Denmark

**Keywords:** *friluftsliv* (outdoor life), green exercise, green space, therapy, social ecology

## Abstract

In this systematic review, we summarized and evaluated the evidence for effects of, and associations between, immersive nature-experience on mental, physical, and social health promotion outcomes. Immersive nature-experience was operationalized as non-competitive activities, both sedentary and active, occurring in natural environments removed from everyday environments. We defined health according to the World Health Organization's holistic and positive definition of health and included steady-state, intermediate, and health promotion outcomes. An electronic search was performed for Danish, English, German, Norwegian, and Swedish articles published between January 2004 and May 2017. Manual approaches, e.g., bibliographies from experts, supplemented the literature search. Data were extracted from 461 publications that met the inclusion criteria. To assess the status and quality of the evidence for health promotion effects of immersive nature-experience, we focused on the subset of studies based on controlled designs (*n* = 133). Outcome level quality of the evidence was assessed narratively. Interventions most often involved adventure-based activities, short-termed walking, and seated relaxation in natural environments. We found positive effects on a range of health promotion outcomes grouped under psychological wellbeing (*n* = 97; ≈55% positive; ≈13% mixed; ≈29% non-significant; 2% negative); psychosocial function (*n* = 67; ≈61% positive; ≈9% mixed; ≈30% non-significant); psychophysiological stress response (*n* = 50; ≈58% positive; ≈18% mixed; ≈24% non-significant), and cognitive performance (*n* = 36; ≈58% positive; ≈6% mixed; ≈33% non-significant; 3% negative); and social skills and relationships (*n* = 34; ≈70% positive; ≈7% mixed; ≈22% non-significant). Findings related to outcomes categorized under physical health, e.g., risk of cardiovascular disease, were less consistent (*n* = 51; ≈37% positive; ≈28% mixed; ≈35% non-significant). Across the types of interventions and outcomes, the quality of the evidence was deemed low and occasionally moderate. In the review, we identify, discuss, and present possible solutions to four core methodological challenges associated with investigating immersive nature-experience and health outcomes: (1) intervention and program complexity; (2) feasibility and desirability of randomization; (3) blinding of participants and researchers; and (4) transferability and generalizability. The results of the review have been published as a popular-scientific report and a scientific research overview, both in Danish language.

## Introduction

Nature may be an affordable, upstream health promotion intervention (Maller et al., 2006) and is widely considered to enhance mental, physical, and social health (Hartig et al., 2011, 2014; Twohig-Bennett and Jones, 2018). However, there are countless ways, situations, and contexts in which nature may be encountered, visited, or used, which in turn may lead to varying health outcomes. While reviews have synthesized the wealth of predominantly correlational literature exploring nature contact and benefits for health (Bowler et al., 2010; Bratman et al., 2012; Hartig et al., 2014; Twohig-Bennett and Jones, 2018), the evidence is both diverse and dispersed. In agreement with Tillmann et al. (2018), we argue that a distinction between types of nature interaction is needed when assessing the evidence, and that existing reviews concerning nature and health have tended to compile interventions that are highly heterogenous (e.g., Twohig-Bennett and Jones, 2018). This approach involves a risk of obscuring the conditions under which contact with nature may or may not promote health outcomes. The consequences are simplified conclusions, reduced interpretational value, and potentially inappropriate health promotion recommendations.

Indicatively, Tillmann et al. (2018) found that that the ratio of positive to non-significant findings varied across three types of nature contact: exposure, i.e., direct and passive or non-specified encounters with natural environments and elements; accessibility, i.e., the likelihood of encountering or interacting with nature; and engagement, i.e., direct, intentional and sustained contact with nature. Exposure to nature and natural elements was most consistently associated with benefits for child and adolescent mental health, whereas accessibility to greenspace and direct engagement with nature provided more mixed results. The difference between the types of contact with nature could be caused by method-related issues or actual differences in achieved outcomes. For example, it is possible that there is a more widespread use of rigorously controlled designs and experimental conditions for exposure-type studies than for accessibility- and engagement-type studies. In this is the case, a focused effort to identify method weaknesses and to improve the quality of the research with consideration and adaptation to the type of nature contact is warranted. However, it is also possible that passive exposure to nature more often provides beneficial outcomes than direct engagement or accessibility to greenspace. This suggests that under some conditions or during specific activities, contact with nature is more likely to have health promoting outcomes than others. This highlights a need for an increased awareness to the context and type of activity involved with nature contact and the circumstances under which positive health promotion outcomes are obtained.

In this systematic review, we focused on the Scandinavian tradition of *friluftsliv*, which includes concepts such as “outdoor life,” “outdoor recreation and education,” or “adventure recreation and education,” but with an emphasis on achieving a closeness to nature during the activity (Gelter, 2000; Sandell, 2003; Bentsen et al., 2009a). While the tradition is considered to be philosophically rooted in an industrialized, Scandinavian setting, activities in nature which encourage the feeling of being away from everyday life and immersion in the experience is practiced more widely (Gelter, 2000; Sandell, 2003; Bentsen et al., 2009a). These criteria are theorized to be fundamental to, for example, restorative experiences according to the Attention Restoration Theory (Kaplan, 1995) which has inspired much research, in both natural and manmade environments, outside of Scandinavia. However, existing reviews of *friluftsliv*, henceforth termed “immersive nature-experience,” were mainly oriented toward Scandinavian practice and published in Scandinavian languages (Sandell, 2004; Schantz and Silvander, 2004). Although highly informative, these reviews were based on narrative identification, quality appraisal, and syntheses of the literature. From a medical, best-evidence paradigm point of view, the quality of the evidence was of low quality (Sandell, 2004; Schantz and Silvander, 2004). In these reviews, the research field anno 2004 was described as vast and interdisciplinary, dominated by qualitative and quantitative, correlational research. Therefore, the aim of this systematic review was to provide an updated, comprehensive overview of the existing research literature about the effects of immersive nature-experience and both mental, physical, and social health promotion outcomes.

Three main research questions frame this systematic review: (1) What types of immersive nature-experience and (2) health outcomes have been investigated, and (3) how do different types of immersive nature-experience influence or associate with mental, physical, and social health promotion outcomes.

We did not consider it meaningful or possible to evaluate the participants' acute and individual experience of being away or closeness to nature in the identified studies. Therefore, we operationalized some, perhaps arbitrary, conditions under which the nature experience should take place for the nature experience to be considered immersive: Inspired by Bentsen et al. (2009a), immersive nature-experience was operationalized as non-competitive activities, both sedentary and active, occurring in public natural environments removed from everyday environments. This, for example, did not include activities in sports fields with greenery, competitive sports in natural environments or transport to and from work or school through natural environments. While private gardens may promote health and afford activities in which individuals may immerse themselves in as well as experience a sense of closeness with nature, they are not removed from everyday life settings. Being away is a central experiential element of the type of immersive nature-experience under review, and we therefore excluded garden-based activities unless they occurred in settings removed from the participants' day-to-day life. All motorized activities in natural environments were excluded. We argue that a strength of this approach involves including nature-based interventions and programs that are more comparable in terms of content, e.g., activities and experiential character of the nature contact, situation, e.g., deliberate visits to nature, and activation of pathways, e.g., direct, multisensory contact with nature, to improved health (Kuo, 2015). To differentiate between contexts and situations of the immersive nature-experience in relation to health promotion outcomes, we divided the individual studies in three rough categories, namely recreation, health and social, and education. These are described in more detail in the methods section.

We defined health according to the World Health Organization's holistic and positive definition of health. Health promotion denotes the process of providing structures and empowering people to exert control over the determinants of health and risk factors (Nutbeam, 1998; Marmot, 2005). In other words, health promotion includes actions directed toward supporting active and healthy living and facilitating supportive environments (Nutbeam, 1998). Inspired by the outcome classification by Nutbeam (1998), we included health and social outcomes, e.g., quality of life and health status, but also proximal outcomes that influence health outcomes, i.e., intermediate health outcomes and health promotion outcomes. Intermediate health outcomes represent the determinants of health outcomes, e.g., lifestyle choices and actions. Health promotion outcomes reflect modulations of those personal, social and environmental factors which are means to improving people's control, e.g., improved health literacy, and thereby changing the determinants of health (intermediate health outcomes). Health literacy could, for example, be specified as physical literacy, which is defined as a person's capacities and attitude for engagement in physical activities (Edwards et al., 2018). In other words, this review covers not only narrowly defined health outcomes, such as functional independence or physical and mental health status, but also actions, e.g., physical activity (PA) and diet, and perceptions, e.g., self-concept and health literacy, that are involved in the process of health promotion. The focus of this review will be individual-oriented outcomes, not environmental or community level outcomes, although these are relevant in evaluations of health promotion interventions more broadly. While we maintain an analytical distinction between mental, physical, and social health promotion outcomes for communicative purposes, these are highly interdependent and developments in one outcome is likely to influence others.

The results of the literature search have previously been published as a popular-scientific report (Mygind et al., 2018a) and a scientific research overview (Mygind et al., 2018b), both in Danish language.

## Methods

The systematic review was inspired by the Preferred Reporting Items for Systematic Reviews and Meta-Analyses (PRISMA) statement (Moher et al., 2009) (for PRISMA checklist, please see Supplementary Material A). The review protocol can be accessed on the PROSPERO register of systematic reviews (ID: CRD42017057988)^1^.

### Eligibility Criteria

Publications were included if reporting on health promotion outcomes of immersive nature-experience (please see the introduction for the definition of immersive nature-experience and health promotion outcomes). We applied no restrictions pertaining to quality of the studies or population characteristics. To assess the status of the evidence concerning effects of the interventions, we focused our analysis on the subset of studies that included a control group. Since little research based on randomized trials was found, we included non-randomized controlled research with attention to the biases involved with this type of research.

We included existing reviews when all included studies investigated immersive nature-experience and health. In some cases, reviews of exposure to nature more broadly were included if it was possible to extract findings related to immersive nature-experience specifically (e.g., Haluza et al., 2014). We included studies that had been published in Danish, English, German, Norwegian, and Swedish language between January 2004 and May 2017. The latter of these criteria was chosen to extend the knowledge from previous reviews about immersive nature-experience and health, which included studies published before 2004 (Sandell, 2004; Schantz and Silvander, 2004).

### Information Sources

Six electronic databases were searched using a generic search string that was adapted to the individual electronic databases (for the generic search string, see Supplementary Material B). These included Dissertation Abstracts, ERIC, PsycINFO, Scopus, SPORTDiscus, and Web of Science. We obtained additional literature on selected websites, through contributions from experts and by searching the reference lists in identified relevant publications.

### Study Selection

**Figure 1:**
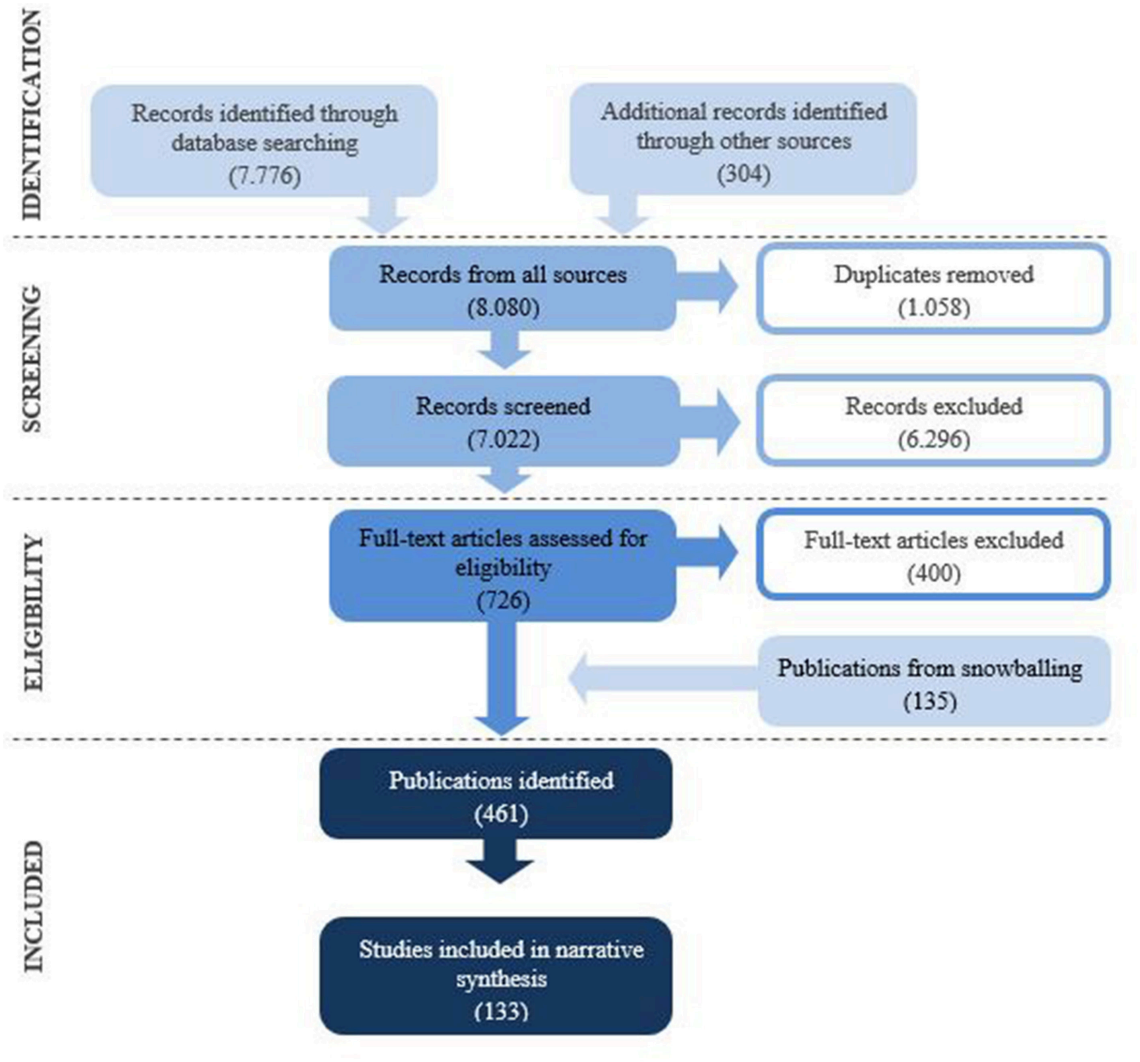
Flow chart.

Identified literature was screened by pairs of two individual reviewers (LM, EK, RH, and EM) by reading through titles and abstracts. Subsequently, full-text eligibility was determined by two independent reviewers. Disagreements were settled through discussion between the two reviewers. If an agreement could not be made, a third reviewer (PB or LM) made the final decision. Please see Figure 1 for PRISMA flow chart.

Two reviewers screened titles and abstracts of 7,022 identified citations, excluding 6,296 publications that were outside the scope of the review. Main reasons for excluding studies at this stage were that studies either investigated only health outcomes, but not immersive nature-exposure, or immersive nature-exposure, but not health.

Subsequently, two reviewers assessed 726 publications in their full length. At this level, studies of types of nature-exposure that did not fall under the definition of immersive nature-experience were excluded. This, for example, included gardening activities (e.g., Sato and Conner, 2013) and school ground greening (e.g., Dyment and Bell, 2008), but also studies in which the place or uses of the natural environments could not be identified (e.g., Zhang et al., 2015). Additional reasons for excluding studies included specifications that, for example, activities occurred indoors (e.g., Siegel et al., 2015); that full-texts could not be obtained (e.g., Jelley, 2005); or the body of the text was written in languages not spoken by members of the review team (e.g., de Assis Pimentel, 2008).

The inclusion of publications identified through snowballing (*n* = 135) resulted in 461 publications included in the systematic review. Within these 461 publications, 489 individual studies were represented since some publications included more individual studies.

### Data Items and Extraction Process

Data were obtained from the literature by a single investigator (LM and EK) using the same generic data extraction form. Data extracted from the literature included: study information (i.e., publication year, authors, and country in which the study was performed); study sample (i.e., sample size, sex, participant characteristics, e.g., information relating to any diagnoses, sociodemographic, or particular group affiliation, and age); study design [inspired by (Ryan et al., 2013)]; activity; duration of exposure; characteristics of natural and control conditions; outcome measures; and reported results. This information is displayed in Tables 1–3.

**Table 1:** Study characteristics, recreation.

**Reference**	**Country**	**Sample size**	**Target group**	**Program content**	**Duration**	**Health domain(s)**	**Outcome(s)**	**Follow-up**
**RANDOMIZED CONTROLLED TRIAL**								
Brown et al., 2014	UK	i: 27 c1: 27 c2: 19	$\bar{x}$ = 40.0 (SD: 10.6), office workers from international firm, ♀♂	Walking (measured during(1) relaxation(2) stressful task(3) after stressful task)	8 weeks (2*20 min weekly)	Mental and Physical	BMI Heart rate Heart rate variability Cardiovascular disease risk score Systolic and diastolic blood pressure Perceived general, physical, and mental health	No
**QUASI-RANDOMIZED CONTROLLED TRIAL**								
Calogiuri et al., 2015	Norway	i: 6 c: 5	$\bar{x}$ = 49 (SD: 8), healthy sedentary or moderately active individuals, ♀♂	Exercise (biking and rubber band exercises)	2 days (2*45 min a day)	Mental and physical	Affective state Blood pressure Serum cortisol Serum cortisol awakening response	Pa.^I^
Mao et al., 2012b	China	i: 10 c: 10	$\bar{x}$ = 20.79 (SD: 0.54), university students, ♂	Walking	2 days [2*1.5 h on the same day (morning and afternoon)]	Mental and Physical	BMI Cortisol Cytokine production Lymphocyte subsets Malondialdehyde level Mood SOD activity Testosterone	No
Bratman et al., 2015b	USA	i: 19 c: 19	$\bar{x}$ = 26.6, healthy adult urban residents, ♀♂	Walking	90 min	Mental and physical	Heart rate Respiration rate Rumination Subgenual prefrontal cortex (sgPFC)	No
Bratman et al., 2015a	USA	i: 30 c: 30	$\bar{x}$= 22.9, university students, ♀♂	Walking	50 min	Mental	Anxiety Cognitive performance Rumination Positive and negative affect	No
Ryan et al., 2010	Australia	80 (i and c N.R.)	18–22, university students, ♀♂	Walking	15 min	Mental	Vitality	No
Perkins et al., 2011	USA	i: 26 c1: 8 c2: 9	19–24, university students, ♀♂	Walking	20 min	Mental	Attention Concentration Mood Short term memory	No
Mayer et al., 2009	USA	76 (i and c N.R.)	N.R., university students, ♀♂	Walking	10 min	Mental	Cognitive performance Positive and negative affect Introverted and extroverted self-consciousness Reflection on negative memory	No
Mayer et al., 2009	USA	92 (i and c N.R.)	N.R., university students, ♀♂	Walking	10 min	Mental	Cognitive performance Positive and negative affect Introverted and extroverted self-consciousness Reflection on negative memory	No
Mayer et al., 2009	USA	64 (i and c N.R.)	N.R., university students, ♀♂	Walking	10 min	Mental	Cognitive performance Positive and negative affect Introverted and extroverted self-consciousness Reflection on negative memory	No
Martens et al., 2011	Switzerland	i: 52 c: 44	$\bar{x}$ = 37.6, students, full time workers, retirees, ♀♂	Walking	30 min	Mental	Positive and negative affect Activation and arousal	No
**CROSS-OVER TRIAL**								
Beil and Hanes, 2013	USA	15	$\bar{x}$ = 42.3 (SD: N.R.), healthy “Non-Hispanic White” adults, ♀♂	Seated relaxation	20 min	Mental	Cortisol Salivary amylase Self-reported stress	No
Berman et al., 2008	USA	38	$\bar{x}$ = 22,62, university students, ♀♂	Walking	55 min	Mental	Cognitive performance	No
Gidlow et al., 2016	UK	38	$\bar{x}$ = 40,9 (SD: 17,6), university students, ♀♂	Walking	30 min	Mental	Cortisol Heart rate variability Mood Working memory	No
Hohashi and Kobayashi, 2013	Japan	27	12–14, ♀	(1) Seated relaxation and (2) walking	(1) 15 min (2) 30 min	Mental	Anxious mood Fatigue Refreshing mood Salivary amylase activity Tension and excitement	No
Johansson et al., 2011	Sweden	20	19–20, university students, ♀♂	Walking	40 min over 5 weeks (with or without friend)	Mental	Positive and negative affect Attention Perceived stress	No
Kerr et al., 2006	Japan	g1^I^: 22 g2: 22	g1: $\bar{x}$ = 22.7 (SD: 1.72) g2: $\bar{x}$ = 20.6 (SD: 1.29), university students, ♂	Running	N.R.	Mental	Somatic feelings Transactional emotions	No
Lee et al., 2009	Japan	12	20–23, university students, ♂	Seated relaxation	15 min	Mental and physical	Cortisol Systolic and diastolic blood pressure	No
Lee et al., 2011	Japan	12	N.R., university students, ♂	Seated relaxation	15 min	Mental and physical	Blood pressure Cortisol Heart rate variability Mood	No
Matsuura et al., 2011	Japan	26	19–25, university students, ♀♂	Horseback riding	30 min	Mental	Salivary alpha-amylase activity Heart rate variability Mood	No
Park et al., 2007	Japan	12	$\bar{x}$ = 22.8 (SD: 1.4), university students, ♂	(1) Walking and (2) seated relaxation	20 min	Mental	Cortisol Hemoglobin concentration in the prefrontal cortex	No
Park et al., 2008	Japan	12	$\bar{x}$ = 21.3 (±SD: 1.1), university students, ♂	Seated relaxation	15 min	Mental and Physical	Blood pressure Cortisol Heart rate variability Pulse	No
Park, 2009	Japan	12	$\bar{x}$ = 21.8 (SD: 0.8), university students, ♂	(1) Seated relaxation and (2) walking	15 min	Mental and Physical	Blood pressure Cortisol Heart rate variability Pulse	No
Park et al., 2010	Japan	280	$\bar{x}$ = 21.7 (SD: 1.5), university students, ♂	(1) Seated relaxation and (2) walking	(1) 14 min (2) 16 min	Mental and physical	Blood pressure Cortisol Heart rate variability Mood Pulse	No
Park et al., 2011	Japan	168	$\bar{x}$ = 20.4 (SD: 4.1), university students, ♀♂	(1) Seated relaxation and (2) walking	15 min	Mental	Mood	No
Roe and Aspinall, 2011a	Scotland	24	g1: $\bar{x}$ = 46, individuals with mental diagnoses g2: $\bar{x}$ = 35, individuals without mental diagnoses, ♀♂	Guided walking	1 h	Mental	Mood Reflection over personal goals Self-esteem	No
Sahlin et al., 2016	Sweden	51	21–72, ♀♂	Guided relaxation	30 min	Mental and physical	Cognitive performance Blood pressure Cognitive fatigue Pulse	No
Schutte et al., 2017	USA	67	4–8, ♀♂	Walking	20 min	Mental	Cognitive performance Inhibitory control Working memory	No
Shin et al., 2011	South Korea	60	$\bar{x}$ = 23.27, university students, ♀♂	Walking	55 min	Mental	Cognitive performance Mood	No
Takayama et al., 2014	Japan	45	19–23, university students, ♂	(1) Seated relaxation and (2) walking	15 min	Mental	Emotional and cognitive restoration Mood Positive and negative affect Vitality	No
Tsunetsugu et al., 2007	Japan	12	$\bar{x}$ = 22.0 (SD: 1.0), university students, ♂	(1) Walking and (2) seated relaxation	15 min	Mental and physical	Cortisol Heart rate variability Systolic and diastolic blood pressure	No
Tyrväinen et al., 2014	Finland	77	$\bar{x}$ = 47.6 (SD: 8.68), ♀♂	(1) Seated relaxation and (2) walking	(1) 15 min (2) 30 min	Mental	Cortisol Creativity Positive and negative affect Vitality	No
Yamaguchi et al., 2006	Japan	10	$\bar{x}$ = 23.2 (SD: 1.1), healthy individuals, ♂	(1) Seated relaxation and (2) walking	20 min	Mental	Cortisol	No
**CONTROLLED BEFORE-AND-AFTER STUDY**								
Bertone, 2015	USA	i: 17 c1: 11 c2: 16	26–75, ♀♂	Yoga	1.5 h per week over 8 to 10 weeks	Mental and physical	Body awareness Cortisol Spiritual wellbeing Quality of life	No
Furman and Sibthorp, 2014	USA	i: 57 c: 60	14–15, ♀♂	Outdoor adventure	14 days	Social	Expedition behavior Prosocial behavior	3 months
Gatersleben and Andrews, 2013	England	i: 17 c: 17	18–43, ♀♂	Walking ^II^	10 min	Mental and physical	Affect Attention Heart rate	No
Li et al., 2008	Japan	i: 12 c: 11	35–56, individuals from four companies in Tokyo, ♂	Walking	3 days, 2 nights	Physical	Adrenaline Granulysin Granzymes Killer cells, amount and activity Lymphocytes Perforin T-cells	1 month (only i)
Orren and Werner, 2007	USA	i:67 c: 76	$\bar{x}$ = 14.64 (SD: 1.91), ♀♂	Wilderness program	1 day, 2 nights	Mental	Self-concept Internalizing and externalizing behaviors	No
Roe and Aspinall, 2011a	Scotland	i:83 c:40	i: $\bar{x}$ = 50 c: $\bar{x}$ = 44, individuals with or without mental diagnoses, ♀♂	Guided walking	1 h	Mental	Mood Reflection over personal goals Self-esteem	No
Thompson, 2014	UK	i: 33 c: 32	$\bar{x}$ = 44.5 (SD: 13.5), ♀♂	Walking	2*45 min per week over 8 weeks	Mental and physical	Arterial stiffness Blood pressure Cognitive function Eating habits Hemodynamic measures Physical activity Self-esteem	No
**WITHIN-SUBJECTS WITHOUT CROSS-OVER**								
Aspinall et al., 2015	Scotland	12	$\bar{x}$ = 30.08 (SD: N.R.), university students, ♀♂	Walking	~10 min	Mental	Activity in frontal cortex Frustration Engagement Arousal Meditation	No
Durr, 2009	USA	20	23–36, volunteers ♀♂	Adventure experience	N.R.	Mental	Subjective wellbeing	No
Fattorini et al., 2012	Italy	14	$\bar{x}$ = 23.9, college students, ♂	Walking	7–15 min	Physical	Oxygen consumption (VO2) Carbon dioxide output Pulmonary ventilation Respiratory exchange ratio Peak oxygen uptake and ventilatory threshold Heart rate	No
Li et al., 2011	Japan	16	36–77, healthy individuals, ♂	Walking	1 day, two sessions	Mental and physical	Adrenaline Noradrenalin Dopamine Blood pressure	No
Li et al., 2016	Japan	19	40–69, ♂	Walking (2.6 km)	1 day, two sessions (80 min)	Mental and physical	Adrenaline Blood pressure Blood sugar Cholesterol Dopamine Mood Noradrenaline Pulse Serum insulin Serum triglyceride	No
Morita et al., 2007	Japan	498^III^	$\bar{x}$ = 56.2 (SD: 10.6), forest visitors, ♀♂	Forest visit ($\bar{x}$ = 5.7 km)	$\bar{x}$ = 2.2 h (SD: 51 min)	Mental	Anxiety Mood	No
Peacock et al., 2007	UK	20	31–70, members of organization that arranges activities in natural environments, ♀♂	Walking	N.R.	Mental	Self-esteem Mood	No
Reed et al., 2013	England	75	11–12, ♀♂	Running	10–20 min	Mental	Self-esteem Enjoyment of running	No
Toda et al., 2013	Japan	20	64–74, healthy individuals, ♂	Walking	45 min	Mental and physical	Blood pressure Cortisol Chromogranin A Pulse Self-reported stress Perceived fatigue and excitement	No

*c, control group; c1, control group number 1; c2, control group number 2; i, intervention group; N.R., not reported; pa., partial; $\bar{x}$ = sample mean (years, km or hours/min)*.

I *Two intervention groups. g1 consisted of occasional male runners, and g2 consisted of specialized male runners*.

II *The publication included two studies: one in which the impacts of qualities of natural environments, high vs. low refuge, were compared during a walk, and another in which a walk in the actual environment was compared to a sitting, indoor condition where the participants viewed the same walk on a screen*.

III *Participants were asked to join the project at entrance to a park. Comparison condition during holiday were participants were asked to fill out the same questionnaire. Therefore, it was not clear what comparison conditions entailed, although authors reported having controlled for physical activity. ♀, female participants; ♂, male participants*.

**Table 2:** Study characteristics, health and social.

**Reference**	**Country**	**Sample size**	**Target group**	**Program content**	**Duration**	**Health domain(s)**	**Outcome(s)**	**Follow-up**
**RANDOMIZED CONTROLLED TRIAL**								
Antonioli and Reveley, 2005	USA and Honduras	i:15 c:15	$\bar{x}$ = 40.2, individuals with diagnosis of mild or moderate depression without psychotic features, ♀♂	Animal-facilitated therapy	1 h per day over two weeks	Mental	Anxiety Depression	No
Lee and Lee, 2014	South Korea	i: 43 c:19	60–80, individuals of which a large proportion were taking various types of medications, ♀	Walking	1 h	Physical	Arterial stiffness Pulmonary function Systolic and diastolic blood pressure	No
Sonntag-Öström et al., 2015	Sweden	i:51 c:48	24–60, individuals diagnosed with exhaustion disorder, ♀♂	Relaxation exercises, seated relaxation in solitude, walking	2*4 h per week over 11 weeks	Mental	Anxiety Attention capacity Depression Fatigue Level of burnout Mood Perceived generalized stress Self-concept Self-esteem Sick leave	12 months
**QUASI-RANDOMIZED CONTROLLED TRIAL**								
Chun et al., 2017	South Korea	i:30 c:29	36–79, individuals with stroke, ♀♂	Forest bathing (meditation and walks)	4 days, 3 nights	Mental	Anxiety Biological antioxidant potentials Depression Reactive oxygen metabolite levels	No
Gelkopf et al., 2013	Israel	i:22 c:20	24–59, patients with chronic war-related PTSD, ♂	Adventure rehabilitation program, sailing and expeditions	3 h per week + 2*3 days over 1 year	Mental and social	Depression PTSD symptoms Social function Quality of life Control of symptoms Hope	No
Hepperger et al., 2016	Austria	i:25 c:23	55–75, patients who had undergone knee surgery, ♀♂	Guided hiking program	2–3 h per week, 3 months	Physical	Functional test (walking on stairs) Muscle strength Self-reported general functionality/wellbeing	2 months
Jelalian et al., 2006	USA	i:37 c:39	13–16, overweight, ♀♂	Adventure therapy + cognitive behavioral therapy	1 session per week, 16 weeks	Mental, social and physical	BMI Peer rejection Self-perception Social support	6 months
Jelalian et al., 2010	USA	i: 62 c: 56	13–16, overweight, ♀♂	Adventure therapy	1 session per week, 16 weeks	Physical	BMI Waist circumference Physical activity	12 months
Jelalian et al., 2011	USA	89 (i and c N.R.)	13–16, overweight, ♀♂	Adventure therapy	1 session per week, 16 weeks	Mental, social and physical	BMI Peer rejection Social anxiety Self-perception	8 months
Johansson et al., 2015	Sweden	i:31 c:7	20–65, individuals with long-lasting mental fatigue after either a traumatic brain injury or stroke, ♀♂	Walking	1 session per week, 8 weeks	Mental	Anxiety Attention capacity Depression Perceived mental fatigue Self-pity	No
Mao et al., 2012a	China	i:12 c:12	60–75, individuals with hypertension, ♀♂	Forest bathing, (walking and relaxation)	2*1.5 h daily over 1 week	Mental and physical	Blood pressure Cytokines (IL-6, TNF) Endothelin Homocysteine Subcomponents of renin-angiotensin system Mood	No
Paquette and Vitaro, 2014	Canada	i1: 101i2: 109	$\bar{x}$ = 20, juvenile delinquents, ♀♂	Wilderness therapy	i1: 8–10 days i2: 17–20 days	Mental and social	Accomplishment motivation Antisocial behaviors Interpersonal skills Socio-professional status	3 and 6 months
Scheinfeld et al., 2016	USA	i:159c:18	22–66, veterans, ♂	Outward bound (OB4V)	6 days	Mental and social	Masculine role conformity Mental health (symptoms, interpersonal relations, and social role performance)	No
Shin et al., 2012	South Korea	i:47 c:45	$\bar{x}$ = 45.26, alcoholics upon inpatient treatment, ♀♂	Forest therapy (games, climbing, walking, orienteering, meditation, coaching)	9 days	Mental	Depression	No
**CLUSTER-RANDOMIZED CONTROLLED TRIAL**								
Zachor et al., 2017	Israel	i: 30c: 21	3.4–7.4, diagnosed with autism, ♀♂	Outdoor adventure	30 min weekly, over 13 weeks	Social	Social functioning	No
**CROSS-OVER TRIAL**								
Berman et al., 2012	USA	20	$\bar{x}$ = 26.0, patients diagnosed with major depressive disorder, ♀♂	Walking	50–55 min	Mental	Short term memory Positive and negative affect Negative memories and feelings toward self	No
Taylor and Kuo, 2009	USA	17	7–12, diagnosed with ADHD, ♀♂	Walking	20 min	Mental	Cognitive performance	No
Frühauf et al., 2016	Austria	14	$\bar{x}$ = 32.7 (SD: 10.8), patients diagnosed with mild to moderate depression, ♀♂	Walking	60 min	Mental	Affective valence Mood	No
hspace*-0.01ptKjellgren and Buhrkall, 2010	Sweden	18	$\bar{x}$ = 36.83, individuals with stress or exhaustion disorders, ♀♂	Seated relaxation	30 min	Mental and physical	Systolic and diastolic blood pressure Perceived stress Pulse	No
Mann, 2007	USA	35	13–17, at-risk, ♀	Outdoor adventure program*	Unclear. Min. 4 days	Mental and social	Identity Mattering Perceived social support Self-confidence Self-esteem	2 weeks
Neunhäuserer et al., 2013	Austria	17	$\bar{x}$ = 43.9 (SD: 8.3), suicidal patients, ♀♂	Hiking	2–3 sessions of 2–2.5 h over 9 weeks	Mental	Cytokines (IL-6, TNF, S100)	No
Roe and Aspinall, 2011a	Scotland	24	g1: $\bar{x}$ = 46, individuals with mental diagnoses g2: $\bar{x}$ = 35, individuals without mental diagnoses, ♀♂	Guided walking	1 h	Mental	Mood Reflection over personal goals Self-esteem	No
Song et al., 2015	Japan	20	$\bar{x}$ = 58, individuals with hypertension, ♂	Walking	17 min	Mental	Heart rate variability Mood	No
Sonntag-Öström et al., 2014	Sweden	20	$\bar{x}$ = 41.6, patients with stress-related burnout syndrome, ♀	Seated relaxation	3*90 min	Mental	Attention capacity Mood Systolic and diastolic blood pressure	No
Sturm et al., 2012	Austria	i1: 10 i2: 10	i1: $\bar{x}$ = 45.1 (SD: 10.4), i2: $\bar{x}$ = 41.0 (SD: 6.3), suicidal patients, ♀♂	Hiking	3*2–3 h per week, 9 weeks	Mental	Depression Hopelessness Thoughts of suicide Sense of belonging	No
**CONTROLLED BEFORE-AND-AFTER STUDY**								
Ang et al., 2014	Singapore	i:76 c:60	13–18, truant behavior, ♀♂	Outward Bound, co-curricular activity	5 days	Mental	Goal setting Problem solving School attendance	1 and 3 months
Barton et al., 2012	England	i: 24 c1: 14 c2: 14	21–83, individuals with various mental diagnoses, ♀♂	Walking	45 min per week, 6 weeks	Mental	Mood Self-esteem	No
Eikenaes et al., 2006	Norway	i:16c:37	i: $\bar{x}$ = 36 (SD: 9.1) c: $\bar{x}$ = 37 (SD: 11.4), patients with avoidant personality disorder, ♀♂	Wilderness therapy	6 days + 3 days (total of 11.5 weeks at hospital)	Mental and social	Avoidance Depression Interpersonal problems Self-efficacy Generalized symptoms of psychopathology	12 months
Gill et al., 2016	USA	i:50c:66	18–39, cancer survivors, ♀♂	Outdoor adventure therapy	7 days	Physical	Self-reported physical activity Perceived barriers for physical activity	3 months
Guthrie, 2004	USA	i: 39c: 33	6–17, mental or emotional disorders, ♀♂	Forest therapy (using Cognitive Behavior Therapy)	2 h weekly, 9 weeks	Mental	Problem Severity Functioning	No
Han et al., 2016	South Korea	i:33 c:28	25–49, individuals with widespread chronic pain, ♀♂	Forest therapy(based on Cognitive Behavior Therapy, for individuals with chronic pain, also includes mindfulness, physical activity, music therapy etc.)	2 days	Mental and physical	Depression Heart rate variability Natural killer cells Perceived pain Quality of life	No
Hough and Paisley, 2008	USA	14^I^	N.R., adults with disabilities, ♀♂	Adventure program	3 days	Mental	Empowerment Situational control	No
Kim et al., 2009	Korea	i: 23 c1: 19 c2: 21	i: $\bar{x}$ = 38.6 (SD: 11) c: $\bar{x}$ = 43.6 (SD: 13.6) c1: $\bar{x}$ = 43.3 (SD: 8.35), patients with major depressive disorder, ♀♂	Forest therapy(using Cognitive Behavior Therapy-Based Psychotherapy)	4 weeks [4 sessions (3 h each)]	Mental	Heart rate variability Cortisol Depression General health	No
Larson, 2007	USA	i:31c: 30	9–17, behavioral issues, ♀♂	Adventure camp	5 days	Mental	Self-concept	No
Margalit and Ben-Ari, 2014	Israel	i:64 c: 29	14–16 at-risk, ♂	Wilderness therapy	Unclear ^II^	Mental	Self-efficacy Cognitive autonomy	5 months
Romi and Kohan, 2004	Israel	i: 36 c1: 88 c2: 33	15–18, drop-outs, ♀♂	Wilderness adventure therapy	6 days	Mental	Self-esteem Locus of control	No
Rosenberg et al., 2014	USA	i: 162 k: 234	18-39, cancer-patients, ♀♂	Outward bound program	6 days	Mental	Body image Self-compassion Self-esteem Depression Alienation	No
Schell et al., 2012	Australia	i: 21 c: 12	12–25, individuals with mental diagnoses, ♀♂	Outdoor adventure group	7 weeks (1 day per week over 6 weeks, then 3 days excursion)	Mental and Social	Self-esteem Self-control Social connectedness	No
Sung et al., 2012	South Korea	i:28 c:28	i: $\bar{x}$ = 63 (SD: 11) c: $\bar{x}$ = 66 (SD: 7), individuals with hypertension, ♀♂	Forest therapy (using Cognitive Behavior Therapy)	8 weeks	Mental and physical	Blood pressure Cortisol Quality of life	1, 4, and 8 weeks
Thomas, 2004	Australia	i:14 c:8	i: $\bar{x}$ = 31.54 (SD: 10.37) c: $\bar{x}$ = 38.38 (SD: 12.14), individuals with acquired brain injury, participants or previous participants in rehabilitation program, ♀♂	Outward bound + outdoor experiential education	9 days + pre and post meetings and activities (total approx. 5–6 months)	Mental	Quality of life	6 months and 2 years
Voruganti et al., 2006	Canada	i:23 c:31	i: $\bar{x}$ = 32.04 (SD: 7.51) c: $\bar{x}$ = 40.83 (SD: 9.44), clinically stabilized schizophrenia patients, ♀♂	Group based adventure therapy	8 months	Mental and social	Cognitive dysfunction Functionality Self-esteem Social, professional, and mental functioning Schizophrenia symptom severity Weight	12 months
Walsh, 2009	USA	i:43 c:43	13–17, referrals from justice system, at-risk and criminals, ♀♂	Wilderness adventure program	21 days (8 days expedition and 4 days solo)	Mental and social	Hope Recidivism Resilience Risk of recidivism Self-efficacy	6 months
Wells, 2004	USA	i: 86 c: 35	12–26, individuals with risky behaviors (and their families), ♀♂	Challenge-based recreation	4 days	Social	Family function Conflict solving	6 weeks
**WITHIN-SUBJECTS WITHOUT CROSS-OVER**								
van den Berg and van den Berg, 2011	Netherlands	12	9–17, diagnosed with ADHD, ♀♂	Stay at care farms	1 h	Mental	Cognitive performance Mood	No

*c, control group, c1, control group number 1; c2, control group number 2; i, intervention group; N.R., not reported, $\bar{x}$ = sample mean (years, km or hours/min)*.

I *Nested control group: i and c both undergo treatment and two different adventure programs*.

II *consisted of participants who participated in a full program (10 weekly preparation meeting involving camping and outdoor skills as well as a 4-day expedition) and partial program. Not clear what a partial program entailed*.

*♀, female participants; ♂, male participants*.

**Table 3:** Study characteristics, education.

**Reference**	**Country**	**Sample size**	**Target group**	**Program content**	**Duration**	**Health domain(s)**	**Outcome(s)**	**Follow-up**
**QUASI-RANDOMIZED CONTROLLED TRIAL**								
Connelly, 2012	USA	i: 18 c: 18	13–16, ♀♂	Adventure-based counseling	1 day	Mental and social	Self-efficacy Self-concept	No
Lethbridge et al., 2005	Canada	i:16 c: 17	$\bar{x}$ = 19, nursing students, ♀♂	Walking (following lecture)	1 h	1 time	Directed attention Attention fatigue Mood Quality of life	No
Mutz and Müller, 2016	Germany	i: 15 c: 7	19–25, university students, ♀♂	Hiking, co-curricular	8 days	Mental	Perceived stress Self-efficacy Attention to surroundings Perceived wellbeing	No
O'Brien and Lomas, 2017	England	i:103 c: 93	N.R., children from three schools, ♀♂	Outdoor personal development course	5 days	Mental	Self-efficacy Resilience Growth mindset	1 month
White, 2012	England	i: 24 c: 24	$\bar{x}$ = 13 (SD: 7), social and emotional challenges, ♀♂	Outdoor Education	3 months	Mental	Self-concept	No
**CROSS-OVER TRIAL**								
Roe and Aspinall, 2011b	Scotland	18	$\bar{x}$ = 11, ♀♂	Forest school	5 h	Mental	Mood Reflection on personal development	No
Wood et al., 2014	England	60	♀: $\bar{x}$ = 13 (SD: 0.5) ♂: $\bar{x}$ = 12.9 (SD: 0.6)	Orienteering	20 min	Mental and physical	Self-esteem Physical activity	No
**CONTROLLED BEFORE-AND-AFTER STUDY**								
American Institutes for Research, 2005	USA	i: 125 c: 109	N.R. (6.th grade students), at-risk, ♀♂	Outdoor science school	5 days	Mental and social	Cooperation Conflict solving skills Self-esteem Problem solving Motivation for learning Academic performance Relationship to peers School behavior	6–10 weeks
Ang et al., 2014	Singapore	i:76 c:60	13–18, truant behavior, ♀♂	Outward bound, co-curricular activity	5 days	Mental	Goal setting Problem solving School attendance	1 and 3 months
Beightol, 2012	USA	i:51 c:54	N.R., fifth grade students, ♀♂	Experiential, adventure-based program (Anti-Bullying Initiative)	10 sessions of 2 h in school setting, 3 excursion days	Mental	Empathy Goal setting Problem solving Resilience Self-efficacy	4 months
Bailey and Kang, 2015	USA	i:95 c:200	N.R., first-year, bachelor art students, ♀♂	Wilderness orientation	10 days (measured over 1 semester)	Mental and Social	Life purpose Self-efficacy Social support	3–4 months
Collins, 2014	USA	i:91 c:65	16–24, university students, ♀♂	Extended wilderness education experience	1 semester	Mental	Problem solving	No
Dettweiler et al., 2017	Germany	i:37 c: 11	$\bar{x}$ = 11.23 (SD: 0.46), ♀♂	Education outside the classroom	1 day weekly over one school term	Mental and physical	Cortisol Physical activity	No
Duerden et al., 2009	USA	i:45 c: 43	11–15, ♀♂	Adventure recreation	2 weeks	Mental	Identity formation antecedents	No
Ewert and Yoshino, 2011	USA	i:28 c:27	N.R., university students, ♀♂	Adventure education	3 weeks (measured over 1 semester)	Mental	Resilience	2 years (interviews)
Fjørtoft, 2004	Norway	i:46 c:29	5–7, ♀♂	Free play	9 months	Physical	Motor skills	No
Foley, 2009	USA	i:193 c:93	+14 years, volunteers, ♀♂	Outward Bound	5–22 days	Mental and social	Leadership skills Personal development Social and environmental awareness	6 months
Frauman and Waryold, 2009	USA	i1: 18 i2: 65 c: 25^I^	N.R., participants in residential learning community and students, ♀♂	Wilderness-based program	4 days (measured over 1 semester)	Mental and Social	Life effectiveness	No
Fuller et al., 2017	England	i:12 c: 12	14–16, underachieving academically, ♀♂	Outdoor residential experiences	Two 3–day sessions yearly, over 3 years	Mental	Academic performance Self-efficacy	No
Gatzemann et al., 2008	Germany	i:26 c:19	19–27 ($\bar{x}$ = 22.79, SD: 1.99), university students (sports), ♀♂	Outdoor education	2*8 days	Mental	Self-value Social self-esteem Bodily self-esteem	No
Gehris, 2007	USA	i: 27 c1: 13 c2: 14	N.R., 10th grade technical students, ♀♂	Adventure education	24 sessions of 40 min over 44 days	Mental and physical	Self-esteem Physical self-concept Physical fitness	No
Johnson-Pynn et al., 2014	Uganda	i:12 c:71	16-24, students from secondary school and members of Wildlife Clubs of Uganda, ♀♂	Environmental education workshops	2–3 days	Mental and social	Self-efficacy Social competencies Leadership skills	No
Harris, 2005	USA	i:29 c:54	16–21, high school students with special needs, ♂	Adventure education	5 days	Mental and social	Academic achievement Self-efficacy School attendance	10 days
Hayhurst et al., 2015	New Zealand	i: 63 c: 63	i: $\bar{x}$ = 16.55, high school students c: $\bar{x}$ = 19.42, university students, ♀♂	Developmental voyage	10 days	Mental	Resilience	No
Hayhurst et al., 2015	New Zealand	i: 72c: 74	i: $\bar{x}$ = 16.51 c: $\bar{x}$ = 16.43, ♀♂	Developmental voyage	10 days	Mental	Resilience Self-esteem Self-efficacy Social effectiveness	5 months
Hunter et al., 2013	New Zealand	i:31 c: 31	$\bar{x}$ = 16.48, ♀♂	Developmental voyage	10 days	Mental	Self-esteem	No
Hunter et al., 2013	New Zealand	i:132 c: 264	i: $\bar{x}$ = 16.28 c: $\bar{x}$ = 17.25, ♀♂	Developmental voyage	10 days	Mental	Self-esteem	12 months
Hunter et al., 2010	New Zealand	i:33 c: 33	i: $\bar{x}$ = 15.79, high school students c: $\bar{x}$ = 19.69, university students, ♀♂	Developmental voyage	10 days	Mental	Self-efficacy	No
Hunter et al., 2010	New Zealand	i:82 c: 31	i: $\bar{x}$ = 15.79 c: $\bar{x}$ = 15, ♀♂	Developmental voyage	10 days	Mental	Self-efficacy	5 months
Kafka et al., 2012	New Zealand	i:27 c: 33	i: $\bar{x}$ = 16.21 c: $\bar{x}$ = 16.25, ♀♂^*^	Developmental voyage	10 days	Mental and social	Self-esteem Gender prejudice	No
Kafka et al., 2012	New Zealand	i:89 c: 53	i: 14–18 c: 15–18, ♀♂	Developmental voyage	10 days	Mental and social	Self-esteem	4–5 months
Darrin Kass, 2011	USA	i:12 c:21	$\bar{x}$ = 26.58, university students, ♀♂	Outdoor Management Training	3–4 days (full course 14 weeks)	Social	Leadership skills	No
McKenzie, 2015	USA	i: 19 c: 14^II^	N.R., primary school students, grades 0 to 5, ♀♂	Access to outdoor classroom	6 months	Social	Social environment in classroom	No
Paquette et al., 2014	USA	i: 32 c1: 17 c2: 35	i: $\bar{x}$ = 17.9 c1: $\bar{x}$ = 18.3 c2: $\bar{x}$ = 16.4, college students, ♀♂	Developmental adventure program	18 days	Mental	Self-esteem	2 months
Sanders et al., 2005	Canada	i:14 c: 18	23–51, registered nurses enrolled in associate degree, ♀♂	Walking	60 min (after lecture)	Mental	Cognitive performance Perceived attention	No
Shirilla, 2009	USA	N.R.	11–12, students enrolled in program, ♀♂	Adventure-based Program	2 school years	Social	Social skills	No
Sibthorp et al., 2015, p. 2015	N.R.	i:32 c:45	$\bar{x}$ = 19.8, volunteers to NOLS course work, ♀♂	Adventure education	3 semesters (70–90 days)	Mental	Self-regulation	4 weeks
Vlamis et al., 2011	USA	i:32 c:45	N.R., first-year college students, ♀♂	Adventure orientation program	6 days	Mental and social	Autonomy Goal setting Interpersonal relations	5 months

*c, control group; c1, control group number 1; c2, control group number 2; I, intervention group, N.R., not reported; $\bar{x}$, sample mean (years, km or hours/min)*.

I *In all groups, there was a large percentage of participant who dropped out of the study from allocation, baseline measures and post measures. i1 consisted of 38 participants, but at the first measurement the number of participants was reduced to 18. Likewise, i2 encompassed 81 participants before the first measurement where only 65 showed up. At the post-measurement, only 35 were included. In c, only 25 participated in pre and post measurements*.

II *Participants were primary school teachers, but the subject of the research was the classroom environment, e.g., relationships between students. ♀, female participants; ♂, male participants*.

According to the context in which the nature-experience was inscribed, studies were divided into three sectors: (1) recreation; (2) social and health; and (3) day care and education. The first category included types of interventions and programs that occurred during healthy participants' free time, e.g., outside school or work hours. The second category encompassed types of interventions and programs that were used as a form of treatment or offer for specific ill, vulnerable or socially disadvantaged populations. The last category included studies that investigated interventions that were integrated in educational practice, e.g., education outside the classroom (EOtC), or add-ons to educational programs, e.g., semester-start courses or adventure education.

### Risk of Bias in and Across Individual Studies

Given the wealth of identified literature and limited resources, we did not systematically assess risk of bias of the individual studies. Risk of bias for individual studies was narratively described. Special attention was given to selection bias related to appropriate randomization of group allocation. In the absence of randomization, we assessed whether recruitment strategies were likely to result in systematic differences between intervention and control groups, and whether sufficient information was provided to ascertain that groups were comparable. Other risk of bias focus areas included ascertainment bias, face-validity of constructs, carry-over effects, as well as attrition, verification, and reporting bias. Lastly, we considered whether power calculations were reported and if sample sizes seemed appropriate. The nature of the interventions makes it impossible to blind participants and instructors to group allocation which might introduce performance bias. However, if this was the only potential source of bias identified, we did not downgrade the quality of evidence at an outcome level. To provide a rough indication of level of evidence, studies were categorized according to the Cochrane Collaboration Study Design Guide (Ryan et al., 2013) from which typical potential sources of bias for the individual types of designs may deduced.

Additionally, we considered the strength of the quantitative, controlled evidence at an outcome level: if most of the studies were randomized, large-scale and without apparent issues relating to bias, we considered the quality of the evidence high. If randomization had been used in most of the studies, but the number of participants was limited, or other issues relating to bias were likely, we considered the quality moderate. If the evidence primarily was based on non-randomized studies, or there were apparent, serious issues relating to bias that could skew the results, we considered the quality of evidence low. If, for example, the effects of immersive nature-experience on psychophysiological stress response was based on a number of non-randomized studies and a few sufficiently powered, appropriately randomized cross-over trials, the quality of the evidence would be considered high. However, if the randomized studies were based on small samples, the quality would be rated moderate. If we additionally found that the studies did not cross-over and counter-balance the order of the exposures, and a carry-over effect would be likely to affect the investigated outcomes, further reductions in the overall assessment of the quality of evidence would be made.

### Summary Measures and Synthesis of Results

Given the heterogeneity of outcomes and interventions, we did not consider meta-analyses appropriate. Results for individual outcomes were therefore not quantitatively synthesized but summarized by sector, health domain, and results and described narratively in the text (Green et al., 2006). Results were divided into four categories: (1) Intervention had significant positive effect on outcome; (2) intervention had significant positive or non-significant effect on subsets of outcome; (3) findings were non-significant; and (4) intervention had significant negative effect on outcome. Mixed findings thus indicated that subsets of the constructs or markers used to measure the same outcome displayed significantly positive changes, but other subsets were non-significant. For example, in a study investigating the effects of immersive nature-experience on mood, individual subscales that contribute to overall mood were considered subscales and not individual constructs. We did not come across any studies in which some subsets were negatively affected and some non-significant or positive and do therefore not include this category. In the text, we present findings according to the categories relating to type of intervention under the three sectors, i.e., recreation, health and social, and education.

## Results

### Study Characteristics

The included studies mainly derived from USA (*n* = 174), UK (*n* = 63), Denmark (*n* = 32), Japan (*n* = 28), Australia (*n* = 24), Norway (*n* = 19), Canada (*n* = 18), New Zealand (*n* = 15), Sweden (*n* = 14), South Korea (*n* = 10), Austria (*n* = 7), Germany (*n* = 5), and Switzerland (*n* = 5). Other countries represented were Finland, Israel, Italy, Iran, Malaysia, Croatia, Bulgaria, Peru, Tanzania, Uganda, and Brazil.

Amongst the 489 included studies, ≈5% were existing reviews (*n* = 23), ≈23% qualitative analyses (*n* = 115), ≈13% a combination of quantitative and qualitative analyses (*n* = 62), and ≈59% quantitative analyses (*n* = 289). Amongst the 315 studies including quantitative analyses, ≈42% involved control groups or conditions (*n* = 133), e.g., randomized controlled-trials, randomized cross-over trials, and controlled before-and-after studies, and ≈69% of the quantitative studies (*n* = 218) did not involve a control group or condition, e.g., before-and-after and case studies. See Tables 1–3 for characteristics of the individual quantitative, controlled studies and Supplementary Material C for a full list of references for qualitative and observational, quantitative studies.

Participants included healthy adults, adolescents and children as well as populations with behavioral or emotional disturbances [e.g., Attention Deficit/Hyperactivity Disorder (ADHD) or depression], substance abuse issues, delinquent behaviors, social disadvantage, or who were overweight. Amongst all the identified research, ≈28% included only child and adolescent populations (*n* = 129) under the age of 18.

Results were grouped under psychological wellbeing (*n* = 97; ≈55% positive; ≈13% mixed; ≈29% non-significant; 2% negative); psychosocial function (*n* = 67; ≈61% positive; ≈9% mixed; ≈30% non-significant); psychophysiological stress response (*n* = 50; ≈58% positive; ≈18% mixed; ≈24% non-significant); cognitive performance (*n* = 36; ≈58% positive; ≈6% mixed; ≈33% non-significant; 3% negative); social skills and relationships (*n* = 34; ≈70% positive; ≈7% mixed; ≈22% non-significant); and physical health, e.g., risk of cardiovascular disease (*n* = 51; ≈37% positive; ≈28% mixed; ≈35% non-significant). See Table 4 for results from all outcomes groups, divided by sector, and Supplementary Material D for all individual outcomes. Table 4, for example, shows that we found 57 studies in which positive results were reported, 22 that reported mixed results, 31 non-significant results, and one negative results on mental health outcomes within the recreation sector.

**Table 4:** Outcomes across sectors and health domains.

	**Recreation**					**Health & Social**					**Education**					**Total**					
	**+**	**+/**	**/**	**–**		**+**	**+/**	**/**	**–**		**+**	**+/**	**/**	**–**		**+**	**+/**	**/**	**–**	**% *p***	**% *p*+m**
**Mental health**	57	22	31	1		50	5	28	1		37	5	12	1		144	32	71	3	57.6	70.4
Psychological wellbeing	22	13	11	0		26	2	16	1		5	0	0	1		53	15	27	2	54.6	70.1
Psychophysiological stress-indicators	23	9	12	0		5	0	0	0		1	0	0	0		29	9	12	0	58.0	76.0
Cognitive indicators	9	0	5	1		5	1	1	0		7	1	6	0		21	2	12	1	58.3	63.9
Psychosocial indicators	3	0	3	0		14	2	11	0		24	4	6	0		41	6	20	0	61.2	70.1
**Physical health**	6	10	7	0		12	4	10	0		1	0	1	0		19	14	18	0	37.3	64.7
Cardiovascular indicators	5	7	5	0		3	3	1	0		0	0	0	0		8	10	6	0	33.3	75.0
Immune function	1	3	1	0		2	1	1	0		0	0	0	0		3	4	2	0	33.3	77.8
Body composition and function	0	0	1	0		4	0	6	0		1	0	1	0		5	0	8	0	38.5	38.5
Active behaviors	0	0	0	0		3	0	2	0		0	0	0	0		3	0	2	0	60.0	60.0
**Social health**	1	0	0	0		15	0	6	0		12	3	4	0		28	3	10	0	68.3	75.6
Supportive environments	0	0	0	0		4	0	2	0		0	1	0	0		4	1	2	0	57.1	71.4
Behaviors	1	0	0	0		3	0	1	0		1	0	1	0		5	0	2	0	71.4	71.4
Skills and relationships	0	0	0	0		8	0	3	0		11	2	3	0		19	2	6	0	70.4	77.8
**Total** ***n***	64	32	38	1		77	9	44	1		50	8	17	1		191	49	99	3	55.8	70.2

*+: Intervention had significant positive effect on outcome (raw count), –: Intervention had significant negative effect on outcome (raw count), +/: Intervention had significant positive or nonsignificant effect on subsets of outcome (raw count), /: Findings were non-significant (raw count), % p: Percentage of total that had positive effect on outcome, %p + m: Percentage of total that had positive or mixed effect on outcome*.

In the following, findings are presented in relation to types of interventions and programs categorized by sector, i.e., recreation, health and social, or education, and domain, i.e., mental, physical, and social health. Any identified meta-analyses and reviews are presented first, followed by supplementary, individual studies.

### Recreational Immersive Nature-Experience

Recreational immersive nature-experience encompassed free-time activities and programs in which healthy participants partook voluntarily. The most common type of activities included short-termed walks or seated relaxation. These activities were sometimes practiced more times over several days in conjunction with cognitive or behavioral therapy. This was often termed *shinrin-yuko* or forest bathing. We found 168 studies that explored relationships between recreational immersive nature-experience and health of which ≈82% (*n* = 138) included mental, ≈52% physical (*n* = 88), and ≈17% social health (*n* = 29) outcomes. Seven reviews addressed various forms of nature-experience in the recreational sector and presented the literature in a manner allowing for extraction of results from studies relating to immersive nature-experience (Thompson, 2006; Bischoff et al., 2007; Tsunetsugu et al., 2010; Bratman et al., 2012; Voutselas, 2012; Konijnendijk et al., 2013; Haluza et al., 2014).

Below, we focus on reviews and studies that were based quantitative, controlled analyses given the wealth of material. Please see Table 1 for a full summary of quantitative, controlled analyses from the recreational sector.

#### Mental Health

##### Psychological wellbeing

Several distinct psychological wellbeing constructs were investigated in the identified literature: positive and negative affect; perceived stress; vitality; quality of life; mental and spiritual wellbeing; anxiety; rumination; internalizing and externalizing behaviors, and mood states. Overall, the number of interventions that had a positive (*n* = 22) or mixed effect on these outcomes (*n* = 13) outweighed the number of non-significant findings (*n* = 11) (see Table 4 for the distribution of positive, negative, mixed, and non-significant findings for the individual outcomes). Mixed findings indicate that subsets of the constructs used to measure the outcome displayed significantly positive changes, but not all. Mixed findings were frequent for the outcome mood states. This is discussed further in the following. Given the predominant use of non-randomized designs with small sample sizes and the dispersion of the findings between and, for the outcome mood states, within the distinct outcome constructs, strong conclusions could not be accumulated, and the quality of the evidence was considered low. Below, we provide examples of the interventions and findings.

Levels of self-reported positive affect were increased relatively more for participants when they walked in natural environments compared to urban environments (Mayer et al., 2009; Johansson et al., 2011; Martens et al., 2011; Takayama et al., 2014; Tyrväinen et al., 2014; Bratman et al., 2015a; Calogiuri et al., 2015), but the same could generally not be observed for negative affect (Mayer et al., 2009; Johansson et al., 2011; Martens et al., 2011; Takayama et al., 2014; Bratman et al., 2015a). Roe and Aspinall (2011a) observed that participants with and without mental diagnoses reported decreased stress and enhanced happiness. This was supported in two other studies evaluating the effects of short-termed walks for healthy adults (Beil and Hanes, 2013; Toda et al., 2013). In another study, no significant stress reducing effects was reported (Johansson et al., 2011). Quality of life and spiritual wellbeing was not found to differ following weekly yoga practice over a period of 8 to 10 weeks in natural environments compared to indoors (Bertone, 2015).

Mixed findings stemmed from studies of the effects of short-termed immersive nature-experience on self-reports of transient mood states, encompassing anxiety, anger, vigor, fatigue, depression, and confusion subscales. Here, we treat these subscales as distinct parts of mood, not as individual constructs. Twelve studies investigated effects of short-termed, i.e., 15 min to 2 days (Peacock et al., 2007; Park et al., 2010, 2011; Lee et al., 2011; Matsuura et al., 2011; Perkins et al., 2011; Shin et al., 2011; Mao et al., 2012b; Hohashi and Kobayashi, 2013; Takayama et al., 2014; Li et al., 2016), and 8 weeks of repeated short-termed recreational immersive nature-experience (Thompson, 2014). In all studies, it was reported that some constructs relating to these mood states were improved, although some subscales remained unchanged. No individual constructs consistently changed across the studies. For example, across short exposures in 14 different types of forests, Park et al. (2011) found that all subscales, except for the construct depression, were improved in comparison to exposures to various urban environments. Comparatively, Peacock et al. (2007) found that anger, confusion, depression, and anxiety were relatively more improved, while no such difference could be observed for vigor and fatigue. Likewise, upon an 8 week program, reductions in anxiety and depression were observed, but not the other mood states (Thompson, 2014). The findings relating to individual, distinct mood states seem to point in various directions. As such, immersive nature-experience may improve mood, but the finer distinctions remain unclear.

##### Psychophysiological stress-indicators

We found that positive findings (*n* = 23) only just outweighed mixed (*n* = 9) and insignificant (*n* = 12) findings across all psychophysiological indicators of stress, i.e., heart rate variability; cortisol; adrenaline; noradrenaline; dopamine; salivary amylase; and cortisol awakening response^2^. More uncommon approaches to measuring acute stress response included activity in the frontal cortex and hemoglobin concentration in the prefrontal area of the brain. The studies were mainly designed as randomized, cross-over trials with small samples. Most of the studies (*n* = 13) included university students of which most were male (85% of the studies). Based on the types of designs and sample sizes used, we considered the overall quality of the evidence moderate. The investigated types of interventions are elaborated in the following and may be supplemented by results from the health and social sectors which are reviewed in section Psychophysiological stress-indicators.

Short-termed walking or seated relaxation in natural environments was frequently (*n* = 9) found to reduce psychophysiological stress indicators (Lee et al., 2009, 2011, 2014; Park et al., 2010; Li et al., 2011, 2016; Mao et al., 2012b; Toda et al., 2013; Aspinall et al., 2015) more than the same activities in control conditions. Amongst these studies, Toda et al. (2013) compared a physically active natural environment condition with a sedentary indoor condition. As such, results may have been affected by differences relating to differences of PA and not the conditions as such. Six studies also investigating the effects of short-termed walking or seated relaxation reported mixed (Park et al., 2007, 2008; Tsunetsugu et al., 2007; Park, 2009; Calogiuri et al., 2015; Lee et al., 2015) and eight insignificant findings (Yamaguchi et al., 2006; Matsuura et al., 2011; Beil and Hanes, 2013; Hohashi and Kobayashi, 2013; Brown et al., 2014; Tyrväinen et al., 2014; Bertone, 2015; Gidlow et al., 2016), but none directly negative outcomes of the programs.

Calogiuri et al. (2015) found that some, but not all, stress-indicators were improved more upon work day breaks involving green exercise (i.e., biking and rubber band exercises) compared to when the same exercises were performed indoors. Brown et al. (2014) did not find an influence of walking in natural environments during breaks in the work day. Likewise, Matsuura et al. (2011) did not find differences in stress-indicators upon a short horseback ride in natural environments compared to an indoor simulator.

##### Cognitive indicators

A number of distinct cognitive indicators were investigated within the recreation sector: cognitive task performance; creativity; attention; concentration; inhibitory control; working memory; and short-term memory. Positive findings marginally outweighed (*n* = 9), negative (*n* = 1), and non-significant findings (*n* = 5). The included studies were designed as randomized cross-over trials with small samples, and the overall quality of evidence was considered moderate.

Short-termed walking in natural environments was frequently found to enhance cognitive performance more than the same activities in control conditions (Berman et al., 2008; Mayer et al., 2009; Shin et al., 2011; Sahlin et al., 2016). Indicators of creativity (Tyrväinen et al., 2014) and working memory (Gidlow et al., 2016) were likewise enhanced more after walking in natural environments compared to urban control conditions. One study reported that no differences relating to attention, concentration, and working memory could be observed when comparing three contexts, i.e., one natural and two built environments (Perkins et al., 2011), and another that participants performed worse in an attention task after a walk in a park (Johansson et al., 2011). We found one study conducted with children in which it was found that preschool children responded more quickly in an attention task following a walk in natural environments compared to urban environments (Schutte et al., 2017), but no differences could be observed relating to regulative control and verbal working memory.

#### Physical Health

##### Cardiovascular indicators

Findings within the recreation sector related to cardiovascular indicators, i.e., acute changes in blood pressure and cardiovascular disease risk factors, were more often absent (*n* = 5) or mixed (*n* = 7) than positive (*n* = 5). The included studies were mainly designed as randomized, cross-over trials with small samples. Based on the types of designs and sample sizes used, we considered the overall quality of the evidence moderate.

Acute reductions in blood pressure were most often mixed (*n* = 6) or insignificant (*n* = 2): only three studies reported larger reductions in blood pressure after exposure to natural than comparison environments. Haluza et al. (2014) reviewed partially overlapping studies using blood pressure measures, amongst other physiological indicators, and echoed the finding. In addition to studies reviewed by Haluza et al. (2014) and Mao et al. (2012b) found that ET-1, a so-called vasoconstrictor involved with the progression of cardiovascular disease, was reduced subject to two walks in natural environments, but not urban environments amongst a small sample of healthy adults. Likewise, Thompson found that arterial stiffness and hemodynamic measures, both associated with cardiovascular risk of disease, were improved differentially following an 8 week walking program (Thompson, 2014). Another indicator of the occurrence and development of cardiovascular disease, platelet activation, was not significantly different (Mao et al., 2012b), nor was the Framingham cardiovascular disease risk score (Brown et al., 2014).

##### Immune function

Four controlled studies investigated the effects of short-termed immersive nature-experience on outcomes related to immune function, i.e., so-called oxidative stress (i.e., the disturbance in the balance between the production of free radicals and antioxidant defenses), pro-inflammatory cytokines (i.e., signaling molecules that mediate innate immune response), and leukocyte or white blood cell subsets. The evidence for impacts of immersive nature-experience on the immune system in healthy subjects was spread over many indicators related to oxidative stress, pro-inflammatory cytokines, and leukocyte subsets. Findings were positive (*n* = 1) or mixed (*n* = 4). The included studies were based on between- and within-subjects designs and included small samples, and the overall quality of evidence was considered low to moderate.

Programs of 3 days including one or two walks in forested areas a day were found to have positive effects on a number of immune function indicators, but some subsets were unchanged. study (Li et al., 2008): Leukocyte subsets, i.e., the number of CD3+ cells; granulsyn; granzymes A/B expressing cells; natural killer cells; and perforin, were increased post nature-programs, but not after the same walking activities in urban environments. However, differences in overall white blood cell counts were non-significant. Another study identified no significant effects on immunoglobin A (Tsunetsugu et al., 2007). A quasi-randomized trial (Mao et al., 2012b) included reports of a vast number of immune function indicators: levels of serum pro-inflammatory cytokines were reduced in the forest group; indicators of oxidative stress status were mixed, i.e., levels of malondialdehyde was decreased, but T-SOD not significantly different; and the distribution of leukocyte subsets was likewise mixed, i.e., the levels of B-lymphocytes were increased, but the percentage of natural killer cells; T; T-helper; and T-suppressor lymphocytes were not significantly different.

#### Social Health

In the absence of controlled studies relating to social health in the recreation sector, we here discuss findings from observational studies. Konijnendijk et al. (2013) reviewed research on the benefits of recreational use of urban parks, including aspects such as social interaction, collective efficacy, and sense of community. The review included mainly correlational studies of which only three studies explicitly addressed immersive nature-experience as defined in the present review. The scarcity of relevant literature identified in this review corresponds with our review. These studies, two qualitative and one observational, quantitative, indicated that adults and children alike considered that urban green space was a place that enhanced social support and social interaction. The authors of the review found that the field of research was limited and deemed the certainty of the evidence weak, as studies were mainly qualitative or observational (Konijnendijk et al., 2013). Due to the use of designs with no control group, the quality of the evidence for the outcomes related to supportive environments, i.e., social interaction and support, was low.

### Immersive Nature-Experience in Social and Health Sectors

One hundred-and-seventeen studies investigated health outcomes of immersive nature-experience in the social and health sectors of which ≈86% included mental (*n* = 147), ≈52% physical (*n* = 89), and ≈36% social health (*n* = 62) outcomes. Thirteen reviews addressed various forms of nature-experience in social and health sectors and presented the literature in a manner allowing for extraction of results from studies relating to immersive nature-experience (Bedard, 2004; Wendell, 2004; Bischoff et al., 2007; Grinde and Patil, 2009; Shanahan et al., 2009; Annerstedt and Währborg, 2011; Lubans et al., 2012; Bowen and Neill, 2013; Ejbye-Ernst, 2013; Norton, 2014; Poulsen et al., 2015; Bettmann et al., 2016; Fernee et al., 2017). Programs were similar in content to those of recreational immersive nature-experience but were targeted and adapted to a range of unhealthy, disadvantaged, or at-risk populations. Intense and demanding expeditions in wild or urban nature or short-and-longer termed primitive camp-based experiences were a predominant type of activity within the sector. Please see Table 2 for a full summary of quantitative, controlled analyses from the social and health sectors.

#### Mental Health

##### Psychological wellbeing

A vast range of outcomes related to psychological wellbeing were investigated in the health and social sector. Some measures represented clinical assessments, i.e., symptoms of psychopathology; schizophrenia; depression; anxiety; and post-traumatic stress disorder (PTSD). Others related to more generalized or non-clinical assessments of positive and negative affect; stress; mood states; depressive states; fatigue; level of burnout; anxiety; thoughts of suicide; negative memories and feelings toward self; quality of life; general wellbeing; and internalizing and externalizing behaviors. Most findings were positive (*n* = 26). One study indicated that fatigue was improved more during the comparison condition, which included a mindfulness course (Johansson et al., 2015). Two studies in the sector reported mixed and 16 non-significant findings. The quality of the evidence was considered low to moderate and is discussed further in the following.

Sustained expedition or base camp adventure experiences in natural environments were commonly used in a variety of social and health care contexts and applied amongst diverse target groups. In a comprehensive meta-analysis based primarily on observational studies, Bowen and Neill (2013) reported that outdoor behavioral health care was associated with moderate effect sizes for psychological state and level of mental functioning, an aggregate measure of parameters such as anxiety and locus of control (*g* = 0.5). However, associations were absent at follow-up. Participants were most commonly male, between age 10 and 17, resided in USA, identified as at-risk and Caucasian (Bowen and Neill, 2013). Populations were characterized as abuse victims; adjudicated youth; behaviorally disordered; disabled; educationally disengaged; emotionally disturbed; having mental health issues; having physical issues (exemplified by brain injury or weight-loss); and substance abusers. The authors observed a substantial level of heterogeneity in the outcome estimates across the individual original studies and found that study, program and, participant characteristics partially explained the variance. Age was highlighted as a singular predictor that influenced the achieved associations: older participants, who more often volunteered to participate than younger participants, achieved larger improvements. Therefore, despite the vastness of the research, the generalizability of the results is questionable. Since subsequent controlled studies have supported the positive findings (e.g., Gelkopf et al., 2013; Scheinfeld et al., 2016), we considered the quality of the evidence for positive associations and effects on psychological wellbeing of adventure experiences in nature low to moderate amongst adolescent and adult populations.

Short-termed seated relaxation and walking in natural environments was found to improve various indicators of self-reported wellbeing. Patients diagnosed with mild to moderate depression reported improved mood indicators and affective valences upon a 60 min walk compared to indoor biking and resting (Frühauf et al., 2016). However, affective valences did not differ significantly from the indoor active control group. Patients with major depressive disorder reported higher levels of positive affect, but similar levels of negative affect, after a 50–55 min of walking in natural environments compared to urban environments (Berman et al., 2012). Likewise, women diagnosed with exhaustion disorder reported improved mood indicators upon three 1.5 h long sessions of seated relaxation in a forest compared to an urban environment (Sonntag-Öström et al., 2015). Mood indicators were likewise higher after 90 min of seated relaxation in three different natural environments compared to a parking lot in an urban area amongst patients with stress-related burnout syndrome (Sonntag-Öström et al., 2014). Amongst two different groups of individuals with and without mental diagnoses, 1 h of guided walking in a natural environment compared to urban environments resulted in higher ratings of mood, with the largest effect sizes observed amongst individuals with mental diagnoses (Roe and Aspinall, 2011a). Similar effects were found for mood indicators amongst hypertensive males after 17 min of walking in a natural environment (Song et al., 2015), compared to urban environments. Thirty minutes of seated relaxation in a forest was found to reduce perceived stress amongst individuals with stress or exhaustion disorders, although no more than the indoor control condition which involved watching a slideshow of the same forest environment (Kjellgren and Buhrkall, 2010). The quality of the evidence was considered moderate being based both randomized and non-randomized controlled designs and small study samples.

Interventions of longer duration with repeated exposures were also investigated. Participants with various mental diagnoses displayed improved mood indicators following a 6-week walking program in natural urban and rural environments, but changes were no larger than two control groups who underwent alternative treatments (social activities and swimming) (Barton et al., 2012). For individuals diagnosed with hypertension, two daily sessions with seated relaxation and walking in forests over a week improved mood indicators more than the same activities in an urban environment did (Mao et al., 2012a).

Patients characterized as high-risk suicidal reported lower levels of depression and hopelessness after a 9 week program with triweekly hikes compared to a waitlist control group (Sturm et al., 2012). Individuals with mental fatigue following acquired brain injury did not report improved anxiety or depression after 8 weeks of weekly 1.5 h walks in a park compared to participants taking part in face-to-face or online mindfulness courses (Johansson et al., 2015).

Two days of forest therapy in conjunction with cognitive behavior therapy provided small effects on self-reported depression and perceived pain amongst individuals with widespread chronic pain (Han et al., 2016). A similar 4-week forest therapy program provided larger reductions in depressive symptoms than a no treatment control and alternative treatment hospital-based control group for patients with major depressive disorder (Kim et al., 2009). Depression and anxiety decreased amongst patients with chronic stroke who partook in a 4 day forest bathing intervention, while no such changes could be observed in the control group who performed similar activities in an urban environment (Chun et al., 2017). Likewise, alcoholics' depression ratings decreased after a 9 day forest therapy camp with no changes occurring in the control group (Shin et al., 2012). The reviewed wellbeing indicators were multifarious, and the included studies covered a range of populations. As such, across various wellbeing measures, immersive nature-experience seemed to be beneficial, but the finer distinctions remained uncertain. The quality of the evidence was considered low to moderate.

##### Psychosocial indicators

There was a vast amount of research indicating that outdoor behavioral health care improved psychosocial parameters (Cason and Gillis, 1994; Hattie et al., 1997; Wilson and Lipsey, 2000; Bedard, 2004; Bowen and Neill, 2013; Bettmann et al., 2016)^3^. The identified psychosocial outcomes represented interrelated yet different phenomena, such as identity formation; confidence; autonomy; locus of control; empowerment; resilience; daily functioning; and self-compassion, -efficacy, -esteem, -concept, -perception, -pity, and -control. Overall, outcomes were often improved (*n* = 14), but many were non-significant (*n* = 11) or mixed (*n* = 2). Due to the predominant use of pre-post designs with no randomization and the dispersion of outcomes, the quality of evidence for these outcomes was considered low.

Bowen and Neill (2013) reported that outdoor behavioral health care was associated with moderate increase in self-concept, which was an aggregate measure of, amongst other parameters, self-efficacy and self-control (*g* = 0.3). Bedard (2004) compared changes in self-esteem and self-concept in delinquent youth, who participated in outdoor behavioral health care with delinquent youth who engaged in standard processes of probation or rehabilitation. Outdoor behavioral health care was considerably more favorable.

##### Psychophysiological stress-indicators

As was observed amongst healthy populations (see section Psychophysiological stress-indicators), short-termed walking or seated relaxation in natural environments was found to reduce psychophysiological stress-indicators, i.e., heart rate variability and cortisol. Amongst the included studies, measures were exclusively found to be improved (*n* = 5). The studies in the social and health sector investigating psychophysiological stress-indicators were mainly designed as controlled before-and-after studies with small samples. Only one study was a cross-over trial. We considered the quality of evidence low to moderate. However, if findings from healthy populations may be translated to the populations included in this section, the evidence could be considered moderate. Examples of interventions and outcomes are provided in the following.

In a randomized cross-over trial, walking for 17 min increased heart rate variability more than the same activities in control conditions for middle-aged males with pre-hypertensive or stage 1 hypertension (Song et al., 2015). Longer-term forest therapy likewise enhanced psychophysiological stress indicators amongst individuals with hypertension (Sung et al., 2012), major depressive disorder (Kim et al., 2009), and chronic pain (Han et al., 2016).

##### Academic achievement and cognitive indicators

Identified outcomes representing cognitive indicators included cognitive performance; attention capacity; short term memory; and goal setting. These were predominantly improved (*n* = 5), but some were mixed (*n* = 1) or non-significant (*n* = 1). Interventions included short-termed walks and seated relaxation, and sustained expeditions and adventure programs. Quality of evidence is discussed in relation to these types of interventions below.

In the meta-analysis described in section Psychological wellbeing, Bowen and Neill (2013) reported that participation in sustained expeditions and adventure programs was associated with moderate improvements in school achievement, e.g., academic performance in English, Math, and Reading (*g* = 0.41). Associations were absent at follow-up. The research was based on studies about outdoor behavioral health care and behaviorally unadjusted adolescents in schools. Therefore, these findings are also relevant to the education sector (section Psychological and psychophysiological wellbeing indicators). The cognitive parameters were scattered and based on mainly observational studies. Given that the studies were based on a wealth of types of designs, many of which utilized poor or no control groups, the quality of the evidence was deemed low.

As was observed amongst healthy populations (see section Cognitive indicators), walking or sitting in nature provided larger acute enhancements in cognitive indicators than the same activity in control conditions: patients diagnosed with major depressive disorder displayed an enhanced short term memory upon walking in a park relatively to an urban environment (Berman et al., 2012). Likewise, women diagnosed with exhaustion disorder (Sonntag-Öström et al., 2015) had improved attention capacity upon three 1.5 h long sessions of seated relaxation in a forest compared to an urban environment. Patients diagnosed with mild to moderate depression participated in an 11 week program consisting of relaxation exercises, seated relaxation in solitude, and walking in a forest environment (Frühauf et al., 2016). Here, it was found that attention capacity was increased after single forest visits, but there was no effect found for the rehabilitation period as a whole when comparing measures to a waitlist control group (Frühauf et al., 2016). Amongst children diagnosed with ADHD (Taylor and Kuo, 2009), acute increases in cognitive performance after a 20 min walk in a natural environment compared to an urban environment. These studies were designed as randomized cross-over trials and one as a randomized controlled trial (RCT), thereby providing a moderate quality of evidence.

#### Physical Health

##### Body composition and physical health

Physical health in the social and health sector was investigated using diverse indicators of bodily function, i.e., pulmonary function; progress in walking rehabilitation; muscle strength; sick leave; perceived pain; and body composition measures, i.e., body-mass index (BMI) and waist circumference. Cardiovascular indicators, i.e., blood pressure and biomarkers of cardiovascular disease risk, and immune function indicators, i.e., oxidative stress, pro-inflammatory cytokines, and leukocyte subsets, were also used. Lastly, various indicators relating to PA were applied (*n* = 5). Most of the studies found that individual outcomes were improved (*n* = 14), but quite a few reported mixed (*n* = 4) and non-significant (*n* = 10) findings. Given that the evidence was predominantly based on observational or controlled pre-post studies without allocation randomization, the quality of the evidence was deemed low.

In the abovementioned meta-analysis, Bowen and Neill (2013) concluded that outdoor behavioral health care was associated with a small effect size related to the participants' bodily function and physical health, exemplified by changes in weight and so-called somatic health (*g* = 0.32). However, associations had decreased at follow-up (*g* = 0.23). A substantial level of heterogeneity in the outcome estimates across the original studies was observed. Hence, the understanding of the mechanisms that cause the observed changes were limited and generalization should be practiced with caution. While the meta-analysis (Bowen and Neill, 2013) primarily was based on observational studies, subsequent experimental, controlled studies have supported the conclusions: outdoor behavioral health care interventions contributed to an enhancement of PA and reductions of the participants' BMI (although no larger than an alternative PA intervention), but improvements were absent at follow-up (Jelalian et al., 2010, 2011). Caution should be taken in interpreting these results since changes in weight and BMI provide inaccurate measures of unhealthy body fat and general bodily health.

#### Social Health

##### Social skills, relationships, and behaviors

Most studies in the social and health sector indicated positive social health outcomes (*n* = 15), although some reported non-significant findings (*n* = 6). The outcomes were spread over a number of measures, for example relating to the participants' social environment, i.e., family function; social support; alienation; and sense of belonging. Social behavior outcomes included antisocial behaviors; social avoidance; and ability to maintain socio-professional status. Lastly, social skills and relationships included outcomes social cognition and functioning; amount of conflict; and interpersonal relationships and problems. Given that the studies utilized a vast number of outcomes and were based on non-randomized designs, the evidence was deemed low to moderate.

In addition to, and partly overlapping with, the results from the above mentioned original studies, Bowen and Neill (2013) concluded that outdoor behavioral health care interventions associated with the participants' social development (*g* = 0.42), e.g., alienation and social skills, and family development (*g* = 0.36), e.g., parent-child relationship and family functioning. Associations were absent by follow-up. Across five original studies, Bedard (2004) found that interpersonal competencies and behavioral change in delinquent youth was positively influenced by participation in outdoor behavioral health care, and that this form of treatment was preferable to standard processes of probation or rehabilitation. Most of the studies on which the reviews were based used non-controlled designs and the quality of evidence was considered low to moderate.

The effects of nature-based sustained expedition or base camp adventure experiences on risky health behaviors were also investigated. Bowen and Neill (2013) collapsed substance use with other parameters, such as recidivism and home behavior, into one behavior-oriented outcome category, and found that the observed associations (*g* = 0.41) remained at follow-up, although reduced (*g* = 0.21). Due to the predominant use of non-controlled designs, the evidence was considered low quality.

### Immersive Nature-Experience in Education

The final sector, immersive nature-experience used in educational context, encompassed 172 individual studies ≈72% included mental (*n* = 124), ≈16% physical (*n* = 27), and ≈58% social health (*n* = 100) outcomes. Three reviews addressed various forms of nature-experience in the education sector and presented the literature in a manner allowing for extraction of results from the studies relating to immersive nature-experience (Furie, 2011; Daniel et al., 2014; Cooley et al., 2015). Adventure education was the main type of activity. Other types of activities included green breaks from teaching and various types of educational activities taking place in natural environments. Please see Table 3 for a full summary of quantitative, controlled analyses from the education and daycare sectors.

#### Mental Health

##### Psychosocial indicators

The dominant type of mental health category in the education sector was psychosocial indicators. Positive findings (*n* = 24) outweighed mixed (*n* = 4) and non-significant (*n* = 6) findings. Frequent outcomes were self-efficacy; self-esteem; and resilience. Other outcomes that were used on a more sporadic basis included self-concept; life effectiveness; sense of identity; autonomy; self-regulation; and the development of a so-called Growth Mindset which is a form of openminded or adaptive thinking. Given that the research was based mainly on small samples and controlled designs with no group allocation randomization, the evidence for the outcomes was considered low quality.

The main type of intervention was sustained adventure-based experiences in natural environments. Adventure education programs ranging from four to ~90 days of varying intensity were found to increase psychosocial indicators such as self-esteem (Romi and Kohan, 2004; Mann, 2007; Kafka et al., 2012; Hunter et al., 2013; Hayhurst et al., 2015), self-efficacy (Hunter et al., 2010; Connelly, 2012; Fuller et al., 2017), self-concept (White, 2012), and resilience (Hayhurst et al., 2015) more than comparison conditions. A few studies in the education sector reported mixed effects on the psychosocial outcomes. For example, Gehris (2007) found that 44 days of adventure education improved the 10th grade pupils' self-esteem more than the control group that had undergone a health education programme, but not more than the control group that participated in a PA program. No effect on self-concept could be observed following the same program in comparison to either control group (Gehris, 2007).

##### Academic achievement and cognitive indicators

Effects of extra- and co-curricular and curriculum-integrated immersive nature-experience on problem solving; goal setting; attention capacity; and academic performance were investigated. Overall, positive effects of the interventions (*n* = 7) were almost matched by the number of non-significant findings (*n* = 6), with one study displaying mixed effects. Since the studies used small samples and did not randomize allocation to groups, the evidence was considered low quality.

Extra- and co-curricular immersive nature-experience for adolescents and young adults provided some positive and non-significant findings. For examples, adventure education as a co-curricular activity for adolescents with truant behavior was found to improve goal setting but not problem solving (Ang et al., 2014) and weekly sessions of outdoor residential experiences improved academically underachieving adolescents' academic performance (Fuller et al., 2017).

Whereas extra- and co-curricular adventure education was usually oriented toward shorter or longer durations of time removed from everyday life, included studies also addressed the potentials of outdoor activities in nature that were inscribed in educational practices. Studies, for example, investigated whether a break from lectures in the form of a 1 h walk in natural environments would increase the capacity to direct attention of undergraduate nursing students (Lethbridge et al., 2005) and diploma-prepared nursing students enrolled in a baccalaureate nursing program (Sanders et al., 2005). Results were inconclusive: in both studies, comparison groups, who did not participate in any form of restorative experience, also increased directed attention scores. In consequence, it seems the results indicated a learning effect rather than an effect of walking in a natural environment. This, however, did not imply inefficacy of natural environments to induce attention restoration, but rather a method weakness related to repeated use of measurement instruments that were not developed for such use. Amongst children, it was found that a 5-day science teaching program in a forest lead to improvements in motivation and attention capacity, as well as academic performance, amongst elementary school children in comparison to similar children who went to school as usual (American Institutes for Research, 2005). Research on EOtC in natural environments, a more long-termed, curriculum-integrated approach than the 5 day science teaching program, resulted in similar findings indicated by an observational study (Mygind, 2005).

##### Psychological and psychophysiological wellbeing indicators

The number of outcomes categorized under psychological wellbeing (positive effects: *n* = 5, negative effect: *n* = 1) and psychophysiological stress-indicators (positive effect: *n* = 1) was limited and we therefore present them together. Included outcomes were mood states; quality of life; mental wellbeing; purpose in life; self-reported stress; and the psychophysiological indicator of stress response, cortisol. Being based on mainly non-randomized study designs and small sample sizes, the evidence was considered low quality.

Interventions ranged from breaks from studying in natural environments (Lethbridge et al., 2005) and curriculum-integrated forest school (Roe and Aspinall, 2011b) to co-curricular hiking trips (Mutz and Müller, 2016). For example, nursing students reported increased mood states and quality of life (Lethbridge et al., 2005). Likewise, children who participated in 5 h of forest school reported improved mood (Roe and Aspinall, 2011b). Cortisol levels, indicative of stress response, were decreased when elementary school students partook in EOtC over 1 year in comparison to typical classroom-based education (Dettweiler et al., 2017).

Interestingly, a 10-day wilderness orientation experience was associated with a decrease in first-year university students' sense of purpose in life (Bailey and Kang, 2015). The authors argued that occurrences unrelated to the program caused the decline in sense of life purpose (Bailey and Kang, 2015). This is probable since outcomes were measured before and after the first semester at university (and not the program), a time of substantial stimuli and rapid development. Furthermore, the study was based on a nonequivalent controlled pre-post design in which participants self-selected into intervention and control groups. As such, the design would be susceptible to influence from these exogenous factors.

#### Physical Health

##### Motor skills and physical activity

We found a limited number of studies about physical health promotion outcomes within the education sector. We identified only two which were based on controlled designs. In these motor skills and physical fitness was assessed. We therefore discuss the observational literature within the sector related to physical health. Considering the scarcity of controlled studies and the use of small samples, the quality of the evidence for these outcomes was considered low.

There were indications that outdoor activities in nature that were integrated in pedagogical practice can enhance preschool-aged children's PA and motor skills. Motor skills are believed to be an important predictor of later life PA (Larsen et al., 2015; Lima et al., 2017a,b). In a preschool context, children's motor skills improved more when participating in daycare taking place in a forest environment compared to children who went to a traditional kindergarten (Fjørtoft, 2004). The observation was supported by an observational study (Vigsø and Nielsen, 2006).

Indicators of PA were mainly used as an acute and highly context-dependent indicator of bodily movement during immersive nature experience. As such, perhaps unsurprisingly, no studies compared PA before and after interventions. Research on EOtC in natural environments, a form of recurring nature-integrated teaching that stems from Scandinavia (Bentsen et al., 2009b; Jordet, 2010), was consistently found to encourage higher levels of moderate to vigorous PA (MVPA) than traditional, classroom-based teaching (Grønningsæter et al., 2007; Mygind, 2007, 2016; Dettweiler et al., 2017). For example, children were estimated to spend 11.5 min longer in MVPA per 2 h unit, averaged over three seasons (Dettweiler et al., 2017).

#### Social Health

##### Social skills and relationships

Social health promotion outcomes within the education sector were most often oriented toward social skills and relationships, i.e., cooperation skills; social competence; amount of conflict and conflict resolution skills; peer relations; interpersonal relations; and knowledge about bullying. Other outcomes included perceived social support; bullying; and school attendance. Mostly, interventions had significant positive effects on outcomes (*n* = 12). Some cases were mixed (*n* = 3) or non-significant (*n* = 4). The quality of the evidence was considered low and is discussed further in relation to the main types of interventions in the following.

A review and three subsequently published original studies reported that various aspects of cooperation skills, such as trust in the group, responsibility, leadership and mutual aid, were improved after both short and long term outdoor education interventions (Frauman and Waryold, 2009; Ewert and Overholt, 2010; Harun and Salamuddin, 2010; White, 2012; Cooley et al., 2015). The review (Cooley et al., 2015) included four controlled studies, two of which were published within the time frame reviewed in the present systematic review (Harun and Salamuddin, 2010; Vlamis et al., 2011), as well as qualitative and observational studies which are not discussed here. The studies included children, adolescents, and adults. While the studies reported promising developments in cooperation skills pre to post programs, the transferability of the skills to everyday life remained uncertain. These types of outdoor education interventions were also found to associate with reduced gender-based prejudice (Kafka et al., 2012) and an increase in knowledge about bullying amongst adolescents, although no direct effect on bullying was observed (Furie, 2011). Effects of adventure-based pre-orientation courses for first-year university students on social competencies (Frauman and Waryold, 2009), social support (Bailey and Kang, 2015), and interpersonal relations (Vlamis et al., 2011) were mixed. The quality of the evidence for effects relating to cooperation skills, gender-based prejudice, bullying, knowledge about bullying, social competences, social support, and interpersonal relations was considered low.

Research on immersive nature-experience integrated into pedagogical and didactical practice provided mixed results. Elementary school students, who took part in a 5-day outdoor science school intervention, rated their conflict resolution and cooperative skills higher than waitlist controls (American Institutes for Research, 2005). Likewise, teachers estimated that the intervention group improved their conflict resolution skills, peer relations, and behavior in class more than the waitlist controls. Over the course of a 6-month outdoor class intervention, McKenzie (2015) could not observe any differences in teacher-reported changes in the quality of peer relations (i.e., friction and cohesion between students) between the intervention and control group. Furthermore, McKenzie (2015) did not report differences between competition orientation and the amount of problems in the class. The intervention and control groups were not completely comparable: students were recruited from different schools and differed in terms of ratio of ethnic minorities and socio-economy with more poor and ethnic minority backgrounds in the intervention group. In supplementary observational studies, it was indicated that participation in EOtC was associated with making more friends and higher levels of social wellbeing (Mygind, 2005, 2009). The used study designs in conjunction with small sample sizes provided the background for considering the quality of the evidence low.

## Discussion

The aims of this systematic review were to investigate (1) what types of immersive nature-experience, (2) which health promotion outcomes had been investigated, and (3) how immersive nature-experience influenced or associated with mental, physical, and social health outcomes. Here, we briefly summarize the key findings relating to (1) prominent types of immersive nature-experience and (2) the most commonly investigated outcomes and associated findings. To provide an overview across the three sectors used above, we also (3) discuss the two main types of immersive nature-experience, short-termed walks, and seated relaxation and adventure-based activities, and associated findings. Since these activities were more often found in the recreational or social and health sectors, we also briefly discuss findings from the educational sector. Lastly, we elaborate on the development of the body of research before discussing strengths and limitations of the present work.

### Prominent Types of Immersive Nature-Experience

Within the recreational sector, the most common type of activity included short-termed walks or seated relaxation, frequently termed *shinrin-yuko* or forest bathing. In the social and health sector, interventions were similar in content to those of recreational immersive nature-experience, but targeted and adapted to a range of unhealthy, disadvantaged, or at-risk populations. The main type of intervention was sustained expeditions and adventure programs in which participants were challenged mentally and physically through activities in natural environments. In the educational sector, adventure education was predominant, but other types of activities included green breaks from teaching and various types of educational activities taking place in natural environments, e.g., forest school or EOtC.

### Prominent Health Promotion Outcomes

Use of immersive nature-experience was most frequently positively, or positively and non-significantly, associated with mental, social, and physical health outcomes. We found conditional support for positive effects on a range of health promotion outcomes grouped under psychological wellbeing (*n* = 97; ≈55% positive; ≈13% mixed; ≈29% non-significant; 2% negative); psychosocial function (*n* = 67; ≈61% positive; ≈9% mixed; ≈30% non-significant); psychophysiological stress response (*n* = 50; ≈58% positive; ≈18% mixed; ≈24% non-significant), and cognitive performance (*n* = 36; ≈58% positive; ≈6% mixed; ≈33% non-significant; 3% negative); and social skills and relationships (*n* = 34; ≈70% positive; ≈7% mixed; ≈22% non-significant). Findings related to outcomes categorized under physical health, e.g., risk of cardiovascular disease, were less consistent (*n* = 51; ≈37% positive; ≈28% mixed; ≈35% non-significant). Only three studies indicated a negative impact of immersive nature-experience on cognitive performance (Johansson et al., 2011), life purpose (Bailey and Kang, 2015), and fatigue (Johansson et al., 2015). Across the sectors, mental health outcomes were the dominant type of outcome. Since these were often psychosocial in character, e.g., self-esteem or self-concept, many mental health outcomes could also be categorized as social health outcomes.

### Health Promotion Outcomes of Short-Termed Immersion in Nature

Across the recreational and social and health sectors, walking, and seated relaxation in natural environments was most frequently found to enhance aspects of wellbeing; reduce stress; and enhance cognitive performance. However, wellbeing measures were diverse and aspects of wellbeing, e.g., individual scales relating to depressive or anxious moods, were not consistently improved. Likewise, the connection between exposure to natural environments while walking or during seated relaxation and acute reductions in psychophysiological indicators of stress was somewhat obscured by a relatively high proportion of mixed or insignificant findings. Much of the research focused on adults, especially university students.

It may seem surprising that the amount of research identified within the field of attention restoration was not larger. In a recent meta-analysis, Ohly et al. (2016) concluded that across 31 studies, some cognitive measures (i.e., Digit Span Forward, Digit Span Backward, and Trial Making Test B) were improved upon exposure to visual representations of natural environments, exposures to natural environments, and views to natural environments. As such, many of these studies were not eligible for inclusion in the present review of immersive nature-experience. However, the findings may supplement the studies identified in the present review.

### Health Promotion Outcomes of Adventure-Based Activities in Nature

Intense and demanding expeditions in wild or urban nature or primitive camp-based experiences targeted toward behavioral change, or personal or social development, appeared to have immediate effects on psychosocial indicators; the ability to engage in social contexts; cooperation skills; family development; behavior (e.g., substance abuse and crime); and physical health (e.g., changes in body weight) across a range of populations. There were indications that behavioral changes endured at follow-up. The activities were mainly inscribed in the educational or social and health sectors. The activities, pedagogies, and places of the programs under investigation were generally poorly described which hinders transfer and reproducibility of the study results. The evidence for the effects of outdoor behavioral health care and outdoor education amongst children is limited, perhaps because the practice is more common amongst adolescents and young adults. For example, the abovementioned meta-analysis (Bowen and Neill, 2013) included only four studies addressing (unspecified) impacts of outdoor education and outdoor behavioral health care amongst children under the age of 9 years.

### Health Promotion Outcomes of Educational Activities in Nature

Use of immersive nature-experience in educational contexts, e.g., schools or kindergartens, was positively associated with mental, social, and physical health outcomes. The research was mainly based on correlational studies that were spread over a range of outcomes and contexts, and we therefore considered the quality of the evidence low. Recent large-scale EOtC studies have supplied that 3 h of weekly EOtC increased boys' MVPA (Schneller et al., 2017a) and girls light PA (Schneller et al., 2017b) considerably over a full 7-day week when compared to students in a traditional classroom setting. Furthermore, the children who participated in EOtC over the course of 1 year maintained a higher level of motivation for school (Bølling et al., 2018). The studies were based on types of EOtC that did not exclusively occur in natural environments, but also other informal learning settings. Subsequent analyses will investigate whether the environments had differential impacts (Nielsen, 2016).

### Developments in the Research Field

In agreement with previous reviews about immersive nature-experience, or *friluftsliv*, we found that the body of research was characterized by a considerable volume and interdisciplinary breadth (Sandell, 2004; Schantz and Silvander, 2004). The main focus of previous reviews was on Scandinavian literature, but attempts were made in previous reviews to also include research published in English language (Sandell, 2004; Schantz and Silvander, 2004). While contributions from outside of Scandinavia predominantly derived from USA at the time of the previous reviews, we unearthed a considerable body of literature from Asian countries relating to the phenomenon of forest bathing or, in Japanese, *shinrin-yuko*.

In previous reviews, the authors reflected upon a development from a culture of practicing immersive nature-experience for the sake of the experience itself toward a more commodified, outcome-driven culture (Sandell, 2004; Schantz and Silvander, 2004). In response to discussions concerning whether this development was within the spirit of immersive nature-experience, Sandell (2004) argued that the continuous growth of immersive nature-experience into other sectors of everyday life should be encouraged and investigated. In the present review, we focused exclusively on health promotion outcomes related to immersive nature-experience within discrete sectors, and the work may thus be considered a continuation and concretization of the outcome-driven focus. While the intermediate health promotion outcomes of immersive nature-experience were discussed in the previous reviews within the context of public health potentials, the main sectors in which immersive nature-experience was practiced were recreational or educational. Perhaps as a consequence of the observed expansion of research and practice of immersive nature-experience, our review differs by including and discussing immersive nature-experience used as an explicit treatment modality, in particular relating to mental and emotional disorders. The present review thus builds on, adds to, and nuances existing insights, rather than presenting a radical paradigm shift in our understanding of the health promotion outcomes of immersive nature-experience.

Direct comparison between health promotion outcomes of immersive nature-experience and non-immersive experience is difficult to make due to the use of varying definitions, categorization, and descriptions of nature contact in the literature (Hartig et al., 2014) as well as pooling of different types of nature-experience in individual reviews (e.g., Twohig-Bennett and Jones, 2018). However, there is empirical evidence to support that the character of the nature contact influences achieved effects. In a recently published meta-analysis (Stevenson et al., 2018), for example, cognitive indicators, e.g., working memory, cognitive flexibility, and attentional control, were found to be significantly higher following direct experience of natural environments compared to indirect exposures, e.g., viewing photos or videos of natural environments. Since our review focused exclusively on direct, immersive contact with natural environments, our results do not allow inferences about this type of nature contact compared with others, e.g., incidental, accumulated exposure due to transport through or views to neighborhood greenery. However, our results suggest that across all outcomes positive effects of immersive nature-experiences occur most consistently for social health [percentage positive (%*p*) = 68.3%, percentage positive and mixed (%*p* + m) = 75.6%) compared to mental (%*p* = 57.6%, %*p* + m = 70.4%) and physical health (%*p* = 43.9%, %*p* + m = 70.2). This suggests that immersive nature-experience might be well-suited for promoting, in particular, social behaviors, skills, and relationships, as well as psychosocial parameters. Since the outcome-level quality assessments were predominantly low, there is some uncertainty regarding the findings. Consequently, subsequent high-quality findings could change the impression made and the knowledge is premature as a background for recommendations for practice.

Although the research connected to physical health was inconsistent, this does not necessarily imply inefficacy. Firstly, the results should be interpreted with attention to the condition that some interventions were compared to active control groups (e.g., Jelalian et al., 2010) and others to waitlist or “treatment as usual” control groups (e.g., Fjørtoft, 2004). Results are influenced by this due to the comparative nature of the research. Secondly, studies in the physical health domain often used many distinct physiological markers to characterize the same phenomenon or closely related phenomena, for example, immune function. If one or more the individual markers were not significantly improved, we categorized the effect as mixed. This might have differentially influenced the physical health domain since many studies in the domain used several markers for the same phenomenon. Mixed findings indicate that positive effects were observed, but interpretation requires caution: Using many markers to address one outcome might be effective for identifying significant effects. In one study (Mao et al., 2012b), for example, six leukocyte subsets were investigated, alongside a number of other physiological indicators. However, in conjunction with small sample sizes, the vast number of variables introduces the risk of finding a significant effect by chance, i.e., type I mistakes. Thirdly, the intermediate health promotion indicator frequently and consistently associated with natural environments, PA (Konijnendijk et al., 2013), was mostly used in observational studies. Furthermore, there is a considerable field of epidemiological research connecting accessibility to green space with PA which was outside the scope of this review. It is well-documented that increased PA and decreased sedentary time reduces obesity, risk of NCDs, such as cardiovascular disease and diabetes, and morbidity (Lee et al., 2012; Ekelund et al., 2016), irrespective of the place where PA takes place. Recently, a connection between PA and cancer has also been established (Monninkhof et al., 2007; Wolin et al., 2009; Speck et al., 2010) and there is sound evidence that PA, in natural outdoor environments or elsewhere, has a direct effect on cognitive function and mental health (Biddle and Asare, 2011). There are indications that PA in natural environments has additional benefits to mental health compared to PA indoors or in urban settings (Bowler et al., 2010), as the present review also suggests. As such, immersive nature-experiences may also contribute to overall PA levels.

### Strengths, Limitations, and Future Perspectives

In the following, we discuss strengths and limitations in the reviewed literature. On this basis, we formulate methodological focus points from which the research field may grow further. Although we focused exclusively on immersive nature-experience and distinguished between types of designs; sectors (e.g., recreation, education, or treatment); and health outcomes, the research was spread across diverse intervention characteristics (e.g., activity, pedagogical and instructional approach, duration or type of natural environment); target groups (e.g., adults and children); and control conditions (e.g., no treatment, short nature exposure, and alternative treatment). As such, we did not consider the research suited for meta-analyses, which, we argue, requires more streamlining to avoid simplified conclusions and recommendations. Elsewhere, the inappropriateness of compiling research conducted with adults and children, for example, with the aim to generalize findings without regard to the target group has been addressed (Tillmann et al., 2018). The aim of this review was to accumulate and evaluate the research relating to specific types of nature-based activity. Therefore, we chose to include both child and adult populations and only distinguish between target groups narratively. Subsequent in-depth analyses of the research pertaining to children will follow.

We did not conduct systematic appraisal of risk of bias in the individual studies. Although we assessed studies according to design and apparent methodological issues, potential sources of bias may have been overlooked. For subsets of the review (psychophysiological outcomes and the literature addressing health promotion outcomes for children and adolescents), we have subsequently performed systematic risk of bias and quality appraisals. The systematic assessments made correspond well with the overall narrative assessments made in this review. Furthermore, we included both randomized and non-randomized controlled studies. The latter type of design is by some considered an inappropriate source for inferences relating to effects (Ryan et al., 2013; Gottfredson et al., 2015). We acknowledge the criticisms that can be raised toward this type of design but chose to include this research due to the scarcity of randomized studies. Since the type of design is so widespread in the reviewed literature, results should be interpreted with care and function only as conditional evidence of effects: Findings may be subject to bias and future rigorous studies might provide other results.

Across the types of interventions and outcomes, the quality of the evidence was deemed low; low to moderate; and occasionally moderate. In many studies, sample sizes were small and possibly underpowered which introduces a risk of small-study effects, i.e., inflated effect sizes and false positives as well as negatives (Schwarzer et al., 2015). Most of the studies identified through the literature searches were qualitative (23.3%, *n* = 114), observational, quantitative (34.6%, *n* = 169), or a mix of qualitative and observational, quantitative (10.2%, *n* = 50). However, in comparison to previous reviews that focused exclusively on immersive nature-experience (Sandell, 2004; Schantz and Silvander, 2004), a growth in quantitative, controlled studies may be discernible. However, since the exact numbers of quantitative, controlled studies were not clear in previous reviews, the comparison is speculative. While previous reviews did not apply systematic quality appraisal methods, the quality of the research was considered low (Sandell, 2004; Schantz and Silvander, 2004). In comparison, the quality of the research included in the present review was most often low or low to moderate, and occasionally moderate at an outcome level. As such, it is possible that a development in the quality has occurred. Still, the knowledge base remains only a conditional basis for effect inference and premature as a basis on which to formulate recommendations for practice. It should be emphasized that the intention behind the interventions was not necessarily health promotion, nor for all original studies to provide gold-standard evidence for health promotion effects of the interventions. However, following the broad and holistic conceptualization of health promotion applied in the present review, we deemed the outcomes relevant for health promotion. We appreciate that some of the outcomes could also have been conceptualized in terms of wellbeing or functioning, which in some cases may have been more in line with the original intentions.

#### Methodological Challenges and Solutions for Future Research

Immersive nature-experience as a subject presents certain core challenges for the conduct of research of the type that is, from a medical, gold-standard point of view, of high quality: (1) immersive nature-experience involves complex interactions between individual subjective experience, activities, pedagogies, and places that are difficult to operationalize and measure in quantitative terms, (2) immersive nature-experiences are often inscribed in real-life contexts in which it is not feasible or desirable to randomize participant allocation, (3) blinding of participants and personnel from group allocation and condition is often not possible, (4) given the complexity of the research subject, transferability between studies and generalizability is challenging. A number of methodological focus points and tools could be used to enhance the quality and transferability of the studies.

The first challenge requires a substantial effort put into formulating a program theory, i.e., identification of intervention input; activities; outputs; intermediate outcomes; and long-term outcomes, and theory of change, i.e., a description of how and why the desired change is expected, pertaining to the specific type of immersive nature-experience and health outcome (Gottfredson et al., 2015). Kuo (2015) and Hartig et al. (2014) reviewed the existing research about nature exposure, in a broad sense, and various health outcomes, and formulated distinct pathways through which exposure to nature could influence health outcomes. The formulated pathways provide handy starting points for formulating theories of change for future studies, but should, at the minimum, be adapted to the specific target group; type of immersive nature-experience; and health outcome. Correspondingly, Hartig et al. (2014) highlighted a need for theory to guide research on which types of nature, and which qualities of those types of nature, are relatively effective for particular outcomes. Based on a review of empirical findings, Kuo (2015) suggested that some pathways are more central or effective than others, and shared Supplementary Materials from which additional pathways may be formulated. Here, we would add that the way the natural environments are “activated” and used, and the social or pedagogical processes that it affords, and not only the types and characteristics of the nature, should be considered in theories of change. The first challenge also necessitates the use of process evaluation to describe and, when possible, quantify the actual implementation of the intervention or mechanisms of action. Recent methodological innovations involved monitoring exposure to EOtC by intervention and comparison teachers (Bølling et al., 2018) and highly increased the understanding of the intervention and findings. The monitoring tool did not concern pedagogical practice or qualities of the environments. Future tools could attempt to also address these aspects.

In the reviewed studies, group allocation was often performed by convenience: participants were often recruited amongst individuals who had self-selected into the programs or who had been directed to the program by, for example, their therapist, teacher, or social worker. Furthermore, sociodemographic group comparability, e.g., age, gender, ethnicity, socioeconomic status (SES), or other relevant background variables of intervention and control groups, was seldom thoroughly described and analyzed. In contrast, the randomized controlled trial is commonly considered the gold-standard that provides the highest quality evidence (Gottfredson et al., 2015). While well-conducted randomization is often possible and provides studies with strong internal validity, it may not always be feasible, ethical, or acceptable, for example, when the subjects are very vulnerable or nested in schools or communities (Gottfredson et al., 2015; Frieden, 2017). Alternatively, regression discontinuity designs and comparison time series designs may provide unbiased estimates of intervention effects, where randomization is not practical or possible (Gottfredson et al., 2015). Other types of quasi-experimental designs that maximize feasibility and acceptability for use in prevention research, which in turn increases ecological validity, includes dynamic wait-listed designs, stepped wedge, or regression point displacement designs (Fok et al., 2015; Wyman et al., 2015). We did not identify any such designs amongst the included studies. Furthermore, the usefulness of experimental research may be increased if supported and supplemented by large, high quality cohort studies (Frieden, 2017). This would also contribute to understandings of nature impacts in a life-course perspective (Hartig et al., 2014). We argue, that an increased detail of reporting about, and general attention to, group allocation generation (preferably randomized, when feasible and ethical) and intervention and control group comparability (including, as a minimum, sociodemographic information such as age, gender, ethnicity, and SES) would significantly improve the quality of evidence.

In clinical research, blinding of participants and personnel from treatment is a fundamental tool to avoid performance bias. Elsewhere, the issues relating to blinding in the context of interventions in which environments and visual representations are a core component have been discussed (Brussoni et al., 2015; Ohly et al., 2016). Although full blinding is not possible, researchers could consider blinding participants and personnel from research aims and questions. In general, studies did not address the extent to which participants or personnel were aware of research aims and questions. As such, this may represent the detail of reporting rather than conduct. We suggest that researchers use blinding of participants and personnel from research aims and questions, to the extent that it is ethically valid. Furthermore, we recommend that the manner of blinding is reported clearly.

The fourth challenge requires an increased transparency and detail in reporting that is currently piecemeal in the reviewed literature: in general, activities; pedagogies; and types and qualities of the natural environments of the interventions and programs under investigation were not presented in sufficient detail. This hinders reproducibility and generalizability of the study results, and ultimately accumulation of solid evidence. The issue transferability and generalizability relates to that of cultural specificity in practices of immersive nature-experience and was also identified by Sandell (2004). Given that practices are culturally and geographically bounded, descriptions of these conditions, approaches, and underlying assumptions should to be described carefully. This challenge could be addressed in a systematic manner by utilizing intervention description checklists, for example the Template for Intervention Description and Replication (TIDieR) (Hoffmann et al., 2014). Using such a checklist will provide a transparency about basic intervention and program information pertaining to, for example, intervention delivery, e.g., physical or informational materials and procedures used; provision, e.g., profession, expertise, and specific training of key intervention providers; mode, e.g., face-to-face or group or alone; location, e.g., place and infrastructure; and intensity, e.g., number of sessions, duration, and schedule. Naturally, the checklist is generic in character and, depending on the specific type of activity and the cultural and geographical context, further description is warranted. For example, information about the intended type and actual practice of pedagogical or psychological approach used, e.g., positive psychology or cognitive-therapy, should be included. Likewise, thorough description of the intervention and control environments are also needed, for example, type of landscape, vegetation, elevation, and season.

In addition to these four methodological challenges and suggestions for solutions, the utility of immersive nature-experience as an affordable, upstream health promotion approach (Maller et al., 2006) would be strongly solidified by supplying studies with cost-effectiveness information (Hartig et al., 2014; Gottfredson et al., 2015). In agreement with Hartig et al. (2014), we suggest that future studies consider reporting cost-effectiveness information, or, in the absence of the resource or skill required, the costs related to interventions (Gottfredson et al., 2015).

## Conclusion

The aim of this systematic review was to summarize and evaluate the evidence for effects of, and associations between, immersive nature experience on mental, physical, and social health. We identified 461 publications including 489 individual studies that met the inclusion criteria. Most studies were qualitative or observational, quantitative. Amongst 133 quantitative analyses in which control groups or conditions had been utilized, we found conditional support for effects of various immersive nature-experience on a range of outcomes: psychological wellbeing (*n* = 97; ≈55% positive; ≈13% mixed; ≈29% non-significant; 2% negative); psychosocial function (*n* = 67; ≈61% positive; ≈9% mixed; ≈30% non-significant); psychophysiological stress response (*n* = 50; ≈58% positive; ≈18% mixed; ≈24% non-significant), and cognitive performance (*n* = 36; ≈58% positive; ≈6% mixed; ≈33% non-significant; 3% negative); and social skills and relationships (*n* = 34; ≈70% positive; ≈7% mixed; ≈22% non-significant). Findings related to outcomes categorized under physical health, e.g., risk of cardiovascular disease, were less consistent (*n* = 51; ≈37% positive; ≈28% mixed; ≈35% non-significant). Walking and seated relaxation in natural environments was most frequently found to enhance aspects of psychological wellbeing, reduce psychophysiological stress, and enhance cognitive performance. Intense and demanding expeditions in wild or urban nature or primitive camp-based experiences targeted toward behavioral change, or personal or social development, promoted psychosocial indicators, the ability to engage in social contexts, cooperation skills, family development, behavior (e.g., substance abuse and crime) across a range of populations.

Across the types of interventions and outcomes, the quality of the evidence was deemed low and occasionally moderate and therefore only serves as a conditional basis for inferences about effects. Based on the main reasons for considering the quality of the evidence low, we discussed four core methodological challenges identified in the reviewed literature and provided tools to address these challenges in future studies: Given the complexity of pathways between immersive nature-experience and health outcomes, studies should develop and be guided by theories of change and develop logic models for the individual immersive nature-experience and health outcome under question. Furthermore, an increased detail of reporting about, and general attention to, group allocation generation (randomized, when ethical and feasible); intervention and control group comparability (including sociodemographic information); blinding (participants, personnel, and outcome assessors); and recruitment and sampling would increase interpretability and quality of the research. Finally, further use of process evaluation and systematic intervention description is warranted. An additional focus point for future research that could provide a strong argument for immersive nature-experience as a health promotion initiative involves reporting costs related to the intervention and, when possible, cost-efficiency.

## Author Contributions

LM, PB, and EM designed and planned the systematic review. LM, EK, and RH performed electronic and supplementary literature searches. All authors contributed to literature screening, full-text eligibility assessments, and data extraction. LM and EK analyzed the literature and wrote up an early paper draft. All authors contributed to the final manuscript.

### Conflict of Interest Statement

The authors declare that the research was conducted in the absence of any commercial or financial relationships that could be construed as a potential conflict of interest.

## Footnotes

^1^http://www.crd.york.ac.uk/PROSPERO/display_record.php?ID=CRD42017057988

^2^Heart rate is sometimes used as an indicator of stress-related phenomena. However, heart rate is subject to a multitude of physiologic cardiac functions and therefore not an interpretationally robust measure of stress.

^3^The majority of the original studies included in the meta-analyses by Bedard (2004) and Bettmann et al. (2016) were also included in the meta-analysis by Bowen and Neill (2013). Since the reviews by Bedard (2004) and Bettmann et al. (2016) focused on different target groups, the overlap between these two reviews was insubstantial: only one original study was included in both. The references (Cason and Gillis, 1994; Hattie et al., 1997; Wilson and Lipsey, 2000) pertain to reviews published previously to the period covered in this review, but were obtained when snowballing references and provide a supplement to the more recent reviews and meta-analysis.
