# Supplementary material for: Immersive Nature-Experiences as Health Promotion Interventions for Healthy, Vulnerable, and Sick Populations? A Systematic Review and Appraisal of Controlled Studies
Source: Front Psychol. 2019 May 3;10:943. doi: 10.3389/fpsyg.2019.00943 (PMC6509207; doi:10.3389/fpsyg.2019.00943)
Supplement: Supplementary file 2 [file Table_2.docx]

**Supplementary material B: Search string**

Example of search string from the electronic database SCOPUS: ( ( TITLE-ABS-KEY ( ( physical W/7 health ) OR ( somatic W/7 health ) OR "well-being" OR illness OR sickness OR "non-communicable disease" OR diabetes OR "cardiovascular disease" OR obesity OR overweight OR "physical activity" OR "physical exercise" OR sedentary OR "active living" OR fitness OR ( mental W/7 health ) OR "cognitive function" OR cognition OR ( psychological W/7 stress ) OR ( life W/7 stress ) OR ( psychological W/7 resilience ) OR ( social W/7 resilience ) OR dementia OR anxiety OR adhd OR "attention deficit" OR attention OR "cognitive flexibility" OR "working memory" OR "executive function" OR "mental disorder" OR "autonomic nervous system" OR "vagal tone" OR "quality of life" OR loneliness OR leadership OR "helping behaviour" OR friendship OR bullying OR "social relation*" OR "peer relati*" OR "pro-social behaviour" OR "self-efficacy" OR "collective efficacy" OR "personal autonomy" OR "social functioning" OR empowerment ) ) AND ( ( ( ( TITLE ( "forest school" OR "forest kindergarten" OR "outdoor learning" OR "outdoor education" OR "outdoor classroom" OR "education outside the classroom" OR "outdoor pedagogy" OR "outdoor experiential education" OR "outdoor teaching" OR "adventure education" OR "outward bound" ) ) ) ) OR ( TITLE ( hiking OR trekking OR mountaineer* OR rock-climb* OR skiing OR "nature visit" OR "park visit" OR "adventure therapy" OR "forest therapy" OR "forest bathing" OR "nature experience" OR "green exercise" OR "outdoor adventure" OR "woodland adventure" OR "outdoor play" OR "adventure sport" ) ) OR ( TITLE ( ( nature W/7 recreation ) OR ( nature W/7 leisure ) OR "outdoor recreation" OR "wilderness therapy" ) ) ) ) OR ( ( TITLE ( ( physical W/7 health ) OR ( somatic W/7 health ) OR "well-being" OR illness OR sickness OR "non-communicable disease" OR diabetes OR "cardiovascular disease" OR obesity OR overweight OR "physical activity" OR "physical exercise" OR sedentary OR "active living" OR fitness OR ( mental W/7 health ) OR "cognitive function" OR cognition OR ( psychological W/7 stress ) OR ( life W/7 stress ) OR ( psychological W/7 resilience ) OR ( social W/7 resilience ) OR dementia OR anxiety OR adhd OR "attention deficit" OR attention OR "cognitive flexibility" OR "working memory" OR "executive function" OR "mental disorder" OR "autonomic nervous system" OR "vagal tone" OR "quality of life" OR loneliness OR leadership OR "helping behaviour" OR friendship OR bullying OR "social relation*" OR "peer relati*" OR "pro-social behaviour" OR "self-efficacy" OR "collective efficacy" OR "personal autonomy" OR "social functioning" OR empowerment ) ) AND ( ( ( ( TITLE-ABS-KEY ( "forest school" OR "forest kindergarten" OR "outdoor learning" OR "outdoor education" OR "outdoor classroom" OR "education outside the classroom" OR "outdoor pedagogy" OR "outdoor experiential education" OR "outdoor teaching" OR "adventure education" OR "outward bound" ) ) ) ) OR ( TITLE-ABS-KEY ( hiking OR trekking OR mountaineer* OR rock-climb* OR skiing OR "nature visit" OR "park visit" OR "adventure therapy" OR "forest therapy" OR "forest bathing" OR "nature experience" OR "green exercise" OR "outdoor adventure" OR "woodland adventure" OR "outdoor play" OR "adventure sport" ) ) OR ( TITLE-ABS-KEY ( ( nature W/7 recreation ) OR ( nature W/7 leisure ) OR "outdoor recreation" OR "wilderness therapy" ) ) ) .
