# Supplementary material for: Immersive Nature-Experiences as Health Promotion Interventions for Healthy, Vulnerable, and Sick Populations? A Systematic Review and Appraisal of Controlled Studies
Source: Front Psychol. 2019 May 3;10:943. doi: 10.3389/fpsyg.2019.00943 (PMC6509207; doi:10.3389/fpsyg.2019.00943)
Supplement: Supplementary file 3 [file Table_3.docx]

**Supplementary material C: Qualitative and observational, quantitative study references**

# Recreation

Annerstedt, M. (2011). *Nature and public health: aspects of promotion, prevention, and intervention*. Swedish University of Agricultural Sciences, Alnarp.

Annerstedt, M., Norman, J., Boman, M., Mattsson, L., Grahn, P., & Währborg, P. (2010). Finding stress relief in a forest. *Ecological Bulletins*, *53*, 33–42.

Atchley, R. A., Strayer, D. L., & Atchley, P. (2012). Creativity in the Wild: Improving Creative Reasoning through Immersion in Natural Settings: e51474. *PLoS One; San Francisco*, *7*(12). http://dx.doi.org.ep.fjernadgang.kb.dk/10.1371/journal.pone.0051474

Bahaeloo-Horeh, S., & Assari, S. (2008). Students Experience Self-Esteem Improvement During Mountaineering. *Wilderness & Environmental Medicine; New York*, *19*(3), 181–5.

Baklien, B., Ytterhus, B., & Bongaardt, R. (2016). When everyday life becomes a storm on the horizon: families’ experiences of good mental health while hiking in nature. *Anthropology & Medicine*, *23*(1), 42–53. https://doi.org/10.1080/13648470.2015.1056056

Barnett, S. (2010). *Aspects of camp ministry that facilitate spiritual growth: A study of two single gender wilderness camps*. Trinity International University. Available at http://search.proquest.com/openview/7303fb7d36aa7b9c5f3fb778687e3771/1?pq-origsite=gscholar&cbl=18750&diss=y

Barton, J., Bragg, R., Pretty, J., Roberts, J., & Wood, C. (2016). The Wilderness Expedition: An Effective Life Course Intervention to Improve Young People’s Well-Being and Connectedness to Nature. *Journal of Experiential Education*, *39*(1), 59–72.

Barton, J., Hine, R., & Pretty, J. (2009). The health benefits of walking in greenspaces of high natural and heritage value. *Journal of Integrative Environmental Sciences*, *6*(4), 261–278. https://doi.org/10.1080/19438150903378425

Brennan, L. A. (2006, december). *A phenomenological inquiry into the experience of reflection by older adults in adventure-based experiential education*. Oregon State University.

Bricker, K. S., Hendricks, W. W., & Aschenbrenner, C. A. (2016). Californians’ Perceptions of the Influence of Parks and Recreation on Quality of Life. *Journal of Park & Recreation Administration*, *34*(3), 64–82. https://doi.org/10.18666/JPRA-2016-V34-I3-7441

Buchecker, M., & Degenhardt, B. (2015). The effects of urban inhabitants’ nearby outdoor recreation on their well-being and their psychological resilience. *Journal of Outdoor Recreation and Tourism*, *10*, 55–62. https://doi.org/10.1016/j.jort.2015.06.007

Castanier, C., Scanff, C. L., & Woodman, T. (2011). Mountaineering as affect regulation: the moderating role of self-regulation strategies. *Anxiety, Stress, & Coping*, *24*(1), 75–89. https://doi.org/10.1080/10615801003774210

Chhetri, P., Arrowsmith, C., & Jackson, M. (2004). Determining hiking experiences in nature-based tourist destinations. *Tourism Management*, *25*(1), 31–43. https://doi.org/10.1016/S0261-5177(03)00057-8

Cole, D. N., & Hall, T. E. (2010). Experiencing the Restorative Components of Wilderness Environments: Does Congestion Interfere and Does Length of Exposure Matter? *Environment and Behavior*, *42*(6), 806–823. https://doi.org/10.1177/0013916509347248

Crust, L., Henderson, H., & Middleton, G. (2013). The acute effects of urban green and countryside walking on psychological health: A field-based study of green exercise. *International journal of sport psychology*, *44*(2), 160–170. https://doi.org/10.7352/IJSP.2013.44.160

Davidson, C. (2016). *Building character through adventure education: A study of levels of grit and resilience in outward bound students*. Indiana University. Available at http://search.proquest.com/openview/70ba3026e4611fb0cf348065f7208f3f/1?pq-origsite=gscholar&cbl=18750&diss=y

Duerden, M. D., Taniguchi, S., & Widmer, M. (2012). Antecedents of Identity Development in a Structured Recreation Setting: A Qualitative Inquiry. *Journal of Adolescent Research*, *27*(2), 183–202. https://doi.org/10.1177/0743558411417869

Dzhambov, A. M., & Dimitrova, D. D. (2014). Elderly visitors of an urban park, health anxiety and individual awareness of nature experiences. *Urban Forestry & Urban Greening*, *13*(4), 806–813. https://doi.org/10.1016/j.ufug.2014.05.006

Evenson, K. R., Wen, F., Hillier, A., & Cohen, D. A. (2013). Assessing the Contribution of Parks to Physical Activity Using Global Positioning System and Accelerometry: *Medicine & Science in Sports & Exercise*, *45*(10), 1981–1987. https://doi.org/10.1249/MSS.0b013e318293330e

Faber Taylor, A., & Kuo, F. E. M. (2011). Could Exposure to Everyday Green Spaces Help Treat ADHD? Evidence from Children’s Play Settings: EVERYDAY GREEN SPACES AND ADHD SYMPTOMS. *Applied Psychology: Health and Well-Being*, *3*(3), 281–303. https://doi.org/10.1111/j.1758-0854.2011.01052.x

Fisker, H. J. (2009). *Unges friluftsliv i Danmark i det 21. århundrede: unges friluftsliv som personligt, socialt og kulturelt identitetsprojekt på firluftslivets felt*. Frederiksberg: Forest & Landscape.

Fuller, R. A., Irvine, K. N., Devine-Wright, P., Warren, P. H., & Gaston, K. J. (2007). Psychological benefits of greenspace increase with biodiversity. *Biology Letters*, *3*(4), 390–394. https://doi.org/10.1098/rsbl.2007.0149

Gatterer, H., Raab, C., Pramsohler, S., Faulhaber, M., Burtscher, M., & Netzer, N. (2015). Effect of weekly hiking on cardiovascular risk factors in the elderly. *Zeitschrift Für Gerontologie Und Geriatrie*, *48*(2), 150–153. https://doi.org/10.1007/s00391-014-0622-0

Goldenberg, M., McAvoy, L., & Klenosky, D. B. (2005). Outcomes from the Components of an Outward Bound Experience. *Journal of Experiential Education*, *28*(2), 123–146.

Hansen, K. B., & Nielsen, T. S. (2005). *Natur og grønne områder forebygger stress*. Friluftsrådet.

Hansmann, R., Hug, S.-M., & Seeland, K. (2007). Restoration and stress relief through physical activities in forests and parks. *Urban Forestry & Urban Greening*, *6*(4), 213–225. https://doi.org/10.1016/j.ufug.2007.08.004

Helge, J. W., Damsgaard, R., Overgaard, K., Andersen, J. L., Donsmark, M., Dyrskog, S. E., … Daugaard, J. R. (2008). Low-intensity training dissociates metabolic from aerobic fitness. *Scandinavian Journal of Medicine & Science in Sports*, *18*(1), 86–94. https://doi.org/10.1111/j.1600-0838.2006.00604.x

Hill, L., Swain, D., & Hill, E. (2008). Energy Balance during Backpacking. *International Journal of Sports Medicine*, *29*(11), 883–887. https://doi.org/10.1055/s-2008-1038492

Hinds, J. (2011). Exploring the psychological rewards of a wilderness experience: An interpretive phenomenological analysis. *The Humanistic Psychologist*, *39*(3), 189–205. https://doi.org/10.1080/08873267.2011.567132

Hinds, J., & Sparks, P. (2011). The Affective Quality of Human-Natural Environment Relationships. *Evolutionary Psychology*, *9*(3), 147470491100900320. https://doi.org/10.1177/147470491100900314

Horiuchi, M., Endo, J., Akatsuka, S., Uno, T., Hasegawa, T., & Seko, Y. (2013). Influence of Forest Walking on Blood Pressure, Profile of Mood States and Stress Markers from the Viewpoint of Aging (JAGv1n1). Hentet 8. november 2017, fra http://savvysciencepublisher.com/downloads/jagv1n1a2/

Houge Mackenzie, S., & Kerr, J. H. (2017). Positive motivational experience over a three-day outdoor adventure trek in Peru. *Journal of Adventure Education and Outdoor Learning*, *17*(1), 4–17. https://doi.org/10.1080/14729679.2016.1189837

Hug, S., Hansmann, R., Monn, C., Krütli, P., & Seeland, K. (2008). Restorative Effects of physical activity in forests and indoor settings. *International Journal of Fitness*, *4*, 25–38.

Humberstone, B., & Stan, I. (2009). Well-being and outdoor pedagogies in primary schooling: The nexus of well-being and safety. *Journal of Outdoor and Environmental Education*, *13*(2), 24.

Hung, K., & Crompton, J. L. (2006). Benefits and Constraints Associated with the Use of an Urban Park Reported by a Sample of Elderly in Hong Kong. *Leisure Studies*, *25*(3), 291–311. https://doi.org/10.1080/02614360500409810

Haaseth, Ø. (2012). Mestring av høydeskrekk i fjellet. *Tidsskrift for Norsk psykologforening*, *49*(9), 838–842.

Irvine, K. N., Warber, S. L., Devine-Wright, P., & Gaston, K. J. (2013). Understanding Urban Green Space as a Health Resource: A Qualitative Comparison of Visit Motivation and Derived Effects among Park Users in Sheffield, UK. *International Journal of Environmental Research and Public Health; Basel*, *10*(1), 417–42.

Jakubec, S. L., Carruthers Den Hoed, D., Ray, H., & Krishnamurthy, A. (2016). Mental well-being and quality-of-life benefits of inclusion in nature for adults with disabilities and their caregivers. *Landscape Research*, *41*(6), 616–627. https://doi.org/10.1080/01426397.2016.1197190

Johan Norman, M. A., & Mattias Boman, L. M. (2010). Influence of outdoor recreation on self-rated human health: comparing three categories of Swedish recreationists. *Scandinavian Journal of Forest Research*, *25*(3), 234–244. https://doi.org/DOI: 10.1080/02827581.2010.485999

Jung, W. H., Woo, J.-M., & Ryu, J. S. (2015). Effect of a forest therapy program and the forest environment on female workers’ stress. *Urban Forestry & Urban Greening*, *14*(2), 274–281. https://doi.org/10.1016/j.ufug.2015.02.004

Kawada, T., Li, Q., itoh-nakadai, A., Inagaki, H., Katsumata, M., Shimizu, T., … Suzuki, H. (2013). Effect of forest bathing on sleep and physical activity. *Forest Medicine*, 105–109.

Ketterer, W. P. (2011). *Psychological change among Appalachian trail thru-hikers: An interpretive phenomenological analysis*. Antioch University New England. Available at http://search.proquest.com/openview/3b1cf60b52acde0d632d492613d0d0bc/1?pq-origsite=gscholar&cbl=18750&diss=y

Kil, N., Stein, T. V., & Holland, S. M. (2014). Influences of wildland–urban interface and wildland hiking areas on experiential recreation outcomes and environmental setting preferences. *Landscape and Urban Planning*, *127*(Supplement C), 1–12. https://doi.org/10.1016/j.landurbplan.2014.04.004

Kline, J. D., Rosenberger, R. S., & White, E. M. (2011). A national assessment of physical activity on US national forests. *Journal of Forestry. 109(6): 343-351*, *109*(6), 343–351.

Korpela, K., Borodulin, K., Neuvonen, M., Paronen, O., & Tyrväinen, L. (2014). Analyzing the mediators between nature-based outdoor recreation and emotional well-being. *Journal of Environmental Psychology*, *37*(Supplement C), 1–7. https://doi.org/10.1016/j.jenvp.2013.11.003

Korpela, K. M., & Yl?n, M. (2007). Perceived health is associated with visiting natural favourite places in the vicinity. *Health & Place*, *13*(1), 138–151. https://doi.org/10.1016/j.healthplace.2005.11.002

Kurtze, N., Eikemo, T., & Hem, K.-G. (2009). *Analyse og dokumentasjon av friluftslivets effekt på folkehelse og livskvalitet*. Norsk Friluftsliv.

Lafortezza, R., Carrus, G., Sanesi, G., & Davies, C. (2009). Benefits and well-being perceived by people visiting green spaces in periods of heat stress. *Urban Forestry & Urban Greening*, *8*(2), 97–108. https://doi.org/10.1016/j.ufug.2009.02.003

Larsen, B., & Juel, K. (2016). Druknedødsfald i Danmark 2001-2014 - og udviklingen 1970-2014. Hentet 8. november 2017, fra http://www.si-folkesundhed.dk/Udgivelser/B%C3%B8ger%20og%20rapporter/2016/Drukned%C3%B8dsfald%20i%20Danmark%202001-2014%20-%20og%20udviklingen%201970-2014.aspx

Larson, L. R., Whiting, J. W., Green, G. T., & Bowker, J. M. (2015). Contributions of Non-Urban State Parks to Youth Physical Activity: A Case Study in Northern Georgia. *Journal of Park & Recreation Administration*, *33*(2), 20–36.

Larson, L., Whiting, J. W., Green, G. T., & Bowker, J. M. (2014). Physical Activity Levels and Preferences of Ethnically Diverse Visitors to Georgia State Parks. *Journal of Leisure Research; Urbana*, *46*(5), 540–562.

Lee, J., & Lee, D. (2015). Nature experience, recreation activity and health benefits of visitors in mountain and urban forests in Vienna, Zurich and Freiburg. *Journal of Mountain Science*, *12*(6), 1551–1561. https://doi.org/10.1007/s11629-014-3246-3

Lemieux, C., Doherty, S., Eagles, P., Gould, J., Hvenegaard, G., Nisbet, E. (Lisa), & Groulx, M. (2015). Healthy Outside-Healthy Inside: The Human Health & Well-being Benefits of Alberta’s Protected Areas - towards a benefits-based management agenda. *Geography and Environmental Studies Faculty Publications*. Available at http://scholars.wlu.ca/geog_faculty/25

Li, Q., Morimoto, K., Kobayashi, M., Inagaki, H., Katsumata, M., Hirata, Y., … Miyazaki, Y. (2008). A forest bathing trip increases human natural killer activity and expression of anti-cancer proteins in female subjects. *Journal of Biological Regulators and Homeostatic Agents*, *22*(1), 45–55.

Li, Q., Morimoto, K., Nakadai, A., Inagaki, H., Katsumata, M., Shimizu, T., … others. (2007). Forest bathing enhances human natural killer activity and expression of anti-cancer proteins. *International journal of immunopathology and pharmacology*, *20*(2_suppl), 3–8.

Lloyd, K., & Little, D. E. (2005). “Quality of life, aren’t we always searching for that?”: How women can achieve enhanced quality of life through participation in outdoor adventure recreation. *Leisure/Loisir*, *29*(2), 147–181. https://doi.org/10.1080/14927713.2005.9651328

Mackay, G. J., & Neill, J. T. (2010). The effect of “green exercise” on state anxiety and the role of exercise duration, intensity, and greenness: A quasi-experimental study. *Psychology of Sport and Exercise*, *11*(3), 238–245. https://doi.org/10.1016/j.psychsport.2010.01.002

MacKerron, G., & Mourato, S. (2013). Happiness is greater in natural environments. *Global Environmental Change*, *23*(5), 992–1000. https://doi.org/10.1016/j.gloenvcha.2013.03.010

Marit Andreassen, Lone Jørgensen, & Bjarne K. Jacobsen. (2007). Fysisk aktivitet i fritiden i Nordland. *Tidsskriftet den Norske Legeforening*, *24*(13). Available at http://tidsskriftet.no/2007/12/aktuelt/fysisk-aktivitet-i-fritiden-i-nordland

Marselle, M., Irvine, K., & Warber, S. (2013). Walking for Well-Being: Are Group Walks in Certain Types of Natural Environments Better for Well-Being than Group Walks in Urban Environments? *International Journal of Environmental Research and Public Health*, *10*(11), 5603–5628. https://doi.org/10.3390/ijerph10115603

Marselle, M. R., Irvine, K. N., Lorenzo-Arribas, A., & Warber, S. L. (2016). Does perceived restorativeness mediate the effects of perceived biodiversity and perceived naturalness on emotional well-being following group walks in nature? *Journal of Environmental Psychology*, *46*(Supplement C), 217–232. https://doi.org/10.1016/j.jenvp.2016.04.008

Marselle, M. R., Irvine, K. N., & Warber, S. L. (2014). Examining Group Walks in Nature and Multiple Aspects of Well-Being: A Large-Scale Study. *Ecopsychology*, *6*(3), 134–147. https://doi.org/10.1089/eco.2014.0027

Marsh, P. E. (2008). Backcountry Adventure as Spiritual Development: A Means-End Study. *Journal of Experiential Education*, *30*(3), 290–293. https://doi.org/10.1177/105382590703000314

McClain, C., & Vandermaas-Peeler, M. (2016). Social contexts of development in natural outdoor environments: children’s motor activities, personal challenges and peer interactions at the river and the creek. *Journal of Adventure Education and Outdoor Learning*, *16*(1), 31–48. https://doi.org/10.1080/14729679.2015.1050682

Mitchell, R. (2013). Is physical activity in natural environments better for mental health than physical activity in other environments? *Social Science & Medicine (1982)*, *91*, 130–134. https://doi.org/10.1016/j.socscimed.2012.04.012

Murray, R., & O’Brien, L. (2005). *Such enthusiasm–a joy to see An evaluation of Forest School in England*. Available at http://forums.forestry.gov.uk/pdf/ForestSchoolEnglandReport.pdf/$FILE/ForestSchoolEnglandReport.pdf

Nawaz, H., & Blackwell, S. (2014). Perceptions about Forest Schools: Encouraging and Promoting Archimedes Forest Schools. *Educational Research and Reviews*, *9*(15), 498–503.

Nisbet, E. K., & Zelenski, J. M. (2011). Underestimating Nearby Nature: Affective Forecasting Errors Obscure the Happy Path to Sustainability. *Psychological Science*, *22*(9), 1101–1106.

Næss, S., & Hansen, T. (2012). Naturelskere og naturbrukere. *Tidsskrift for samfunnsforskning*, *53*(04), 406–427.

O’Brien, L. (2009). Learning outdoors: the Forest School approach. *Education 3-13*, *37*(1), 45–60. https://doi.org/10.1080/03004270802291798

O’Brien, L., Burls, A., Townsend, M., & Ebden, M. (2011). Volunteering in nature as a way of enabling people to reintegrate into society. *Perspectives in Public Health; London*, *131*(2), 71–81.

O’Brien, L., & Murray, R. (2007). Forest School and its impacts on young children: Case studies in Britain. *Urban Forestry & Urban Greening*, *6*(4), 249–265. https://doi.org/10.1016/j.ufug.2007.03.006

O’Brien, L., Townsend, M., & Ebden, M. (2008). *Environmental volunteering: motivations, barriers and benefits*. UK: Scottish Forestr y Trust and Forestr y Commission.

Orsega-Smith, E., Mowen, A. J., Payne, L. L., & Godbey, G. (2004a). The Interaction of Stress and Park Use on Psycho-physiological Health in Older Adults. *Journal of Leisure Research; Urbana*, *36*(2), 232–256.

Ottosson, J., & Grahn, P. (2008). The Role of Natural Settings in Crisis Rehabilitation: How Does the Level of Crisis Influence the Response to Experiences of Nature with Regard to Measures of Rehabilitation? *Landscape Research*, *33*(1), 51–70. https://doi.org/10.1080/01426390701773813

Peacock, J., Hine, R., & Pretty, J. (2007). *The mental health benefits of green exercise activities and green care*. Mind.

Plambech, A. T. (2016). Naturen kan fremme din kreativitet. Available at http://natur-vejleder.dk/wp-content/uploads/2016/03/september_2015_kreativitet.pdf

Pons-Villanueva, J., Segui-Gomez, M., & Martinez-Gonzalez, M. A. (2010). Risk of injury according to participation in specific physical activities: a 6-year follow-up of 14 356 participants of the SUN cohort. *International Journal of Epidemiology*, *39*(2), 580–587. https://doi.org/10.1093/ije/dyp319

Pretty, J., Peacock, J., Hine, R., Sellens, M., South, N., & Griffin, M. (2007). Green exercise in the UK countryside: Effects on health and psychological well-being, and implications for policy and planning. *Journal of Environmental Planning and Management*, *50*(2), 211–231. https://doi.org/10.1080/09640560601156466

Puhakka, R., Pitkänen, K., & Siikamäki, P. (2016). The health and well-being impacts of protected areas in Finland. *Journal of Sustainable Tourism*, 1–18. https://doi.org/10.1080/09669582.2016.1243696

Rader, S. S. (2009). *Ecopsychology revealed: An empirical look at the benefits of nature experience for human beings and the world*. ALLIANT INTERNATIONAL UNIVERSITY, LOS ANGELES. Available at http://gradworks.umi.com/33/68/3368111.html

Randrup, T. B. (2008). *Sammenhæng mellem grønne områders udtryk og brug set i forhold til befolkningens sundhed*. Available at http://naturstyrelsen.dk/media/nst/66759/NaturOgSundhed.pdf

Richardson, M., & Hallam, J. (2013). Exploring the psychological rewards of a familiar semirural landscape: Connecting to local nature through a mindful approach. *The Humanistic Psychologist*, *41*(1), 35–53. https://doi.org/10.1080/08873267.2012.732156

Roberson Jr, D. N., & Babic, V. (2008). Walking and Hiking as a Way of Life. *Online Submission*. Available at http://eric.ed.gov/?i*d=*ED501659

Robinson, R. A. (2013, juli). *Described experiences of long-distance thru-hiking: A qualitative content analysis*. Capella University.

Roemmich, J. N., & Johnson, L. (2014). Seasonal Alterations in Park Visitation, Amenity Use, and Physical Activity — Grand Forks, North Dakota, 2012–2013. *Preventing Chronic Disease*, *11*. https://doi.org/10.5888/pcd11.140175

Rogerson, M., Brown, D. K., Sandercock, G., Wooller, J.-J., & Barton, J. (2016). A comparison of four typical green exercise environments and prediction of psychological health outcomes. *Perspectives in Public Health*, *136*(3), 171–180. https://doi.org/10.1177/1757913915589845

Sanderud, J. R., & Gurholt, K. P. (2015). Nysgjerrig lek: Utforskende dannelse. I *Rapport fra konferansen* (s. 218–224). Lillehammer, Norge: Norsk Friluftsliv.

Shanahan, D. F., Bush, R., Gaston, K. J., Lin, B. B., Dean, J., Barber, E., & Fuller, R. A. (2016). Health Benefits from Nature Experiences Depend on Dose. *Scientific Reports*, *6*(1). https://doi.org/10.1038/srep28551

Sherk, V., Sherk, K. A., Kim, S., Young, K., & Bemben, D. (2010). Hormone responses to a continuous bout of rock climbing in men. *Eur J Appl Physiol (2011)*, *111*, 687–693. https://doi.org/DOI 10.1007/s00421-010-1685-2

Shin, W. S., Kwon, H. G., Hammitt, W. E., & Kim, B. S. (2005). Urban forest park use and psychosocial outcomes: A case study in six cities across South Korea. *Scandinavian Journal of Forest Research*, *20*(5), 441–447. https://doi.org/10.1080/02827580500339930

Skår, M., & Krogh, E. (2009). Changes in children’s nature-based experiences near home: from spontaneous play to adult-controlled, planned and organised activities. *Children’s Geographies*, *7*(3), 339–354. https://doi.org/10.1080/14733280903024506

Soga, M., Cox, D. T. C., Yamaura, Y., Gaston, K. J., Kurisu, K., & Hanaki, K. (2017). Health Benefits of Urban Allotment Gardening: Improved Physical and Psychological Well-Being and Social Integration. *International Journal of Environmental Research and Public Health*, *14*(1), 71. https://doi.org/10.3390/ijerph14010071

Sutherland, S., & Stroot, S. (2010). The Impact of Participation in an Inclusive Adventure Education Trip on Group Dynamics. *Journal of Leisure Research; Urbana*, *42*(1), 153–176.

Tardona, D. (2014). A Program Encouraging Healthy Behavior, Nature Exploration, and Recreation through History in an Urban National Park Unit. *Journal of Park and Recreation Administration*, *32*, 73–82.

Tian, Y., He, Z., Xu, C., Huang, C., Lee, J.-H., Li, R., … Mc Naughton, L. (2015). Energy Expenditure and Fitness Response Following Once Weekly Hill Climbing at Low Altitude. *International Journal of Sports Medicine*, *36*(05), 357–364. https://doi.org/10.1055/s-0034-1395520

van den Berg, M., van Poppel, M., van Kamp, I., Andrusaityte, S., Balseviciene, B., Cirach, M., … Maas, J. (2016). Visiting green space is associated with mental health and vitality: A cross-sectional study in four european cities. *Health & Place*, *38*, 8–15. https://doi.org/10.1016/j.healthplace.2016.01.003

Vieira, M. F., de Avelar, I. S., Silva, M. S., Soares, V., & Lobo da Costa, P. H. (2015). Effects of Four Days Hiking on Postural Control. *PLOS ONE*, *10*(4), e0123214. https://doi.org/10.1371/journal.pone.0123214

W. Manning, J., Montes, J., Stone, T., W. Rietjens, R., Young, J., DeBeliso, M., & W. Navalta, J. (2015). Cardiovascular and Perceived Exertion Responses to Leisure Trail Hiking. *Journal of Outdoor Recreation, Education, and Leadership*, *7*, 83. https://doi.org/10.18666/JOREL-2015-V7-I2-7005

Whitaker, E. D. (2005). The Bicycle Makes the Eyes Smile: Exercise, Aging, and Psychophysical Well-Being in Older Italian Cyclists. *Medical Anthropology*, *24*(1), 1–43. https://doi.org/10.1080/01459740590905633

White, M. P., Elliott, L. R., Taylor, T., Wheeler, B. W., Spencer, A., Bone, A., … Fleming, L. E. (2016). Recreational physical activity in natural environments and implications for health: A population based cross-sectional study in England. *Preventive Medicine*, *91*, 383–388. https://doi.org/10.1016/j.ypmed.2016.08.023

White, M. P., Pahl, S., Ashbullby, K., Herbert, S., & Depledge, M. H. (2013). Feelings of restoration from recent nature visits. *Journal of Environmental Psychology*, *35*, 40–51. https://doi.org/10.1016/j.jenvp.2013.04.002

Whittington, A., Mack, E. N., Budbill, N. W., & McKenney, P. (2011). All-girls adventure programmes: what are the benefits? *Journal of Adventure Education and Outdoor Learning*, *11*(1), 1–14. https://doi.org/10.1080/14729679.2010.505817

Wittington, A. (2006). Challenging Girls’ Constructions of Femininity in the Outdoors. *Journal of Experiential Education*, *28*(3), 205–221.

Wolf, I. D., & Wohlfart, T. (2014). Walking, hiking and running in parks: A multidisciplinary assessment of health and well-being benefits. *Landscape and Urban Planning*, *130*, 89–103. https://doi.org/10.1016/j.landurbplan.2014.06.006

Wu, C.-L. (2004). *The effect of gender role orientation and participation in a single sex outdoor recreation program on self -efficacy* (Ed.D.). Oklahoma State University, United States -- Oklahoma. Available at http://search.proquest.com.ludwig.lub.lu.se/pqdt/docview/305085551/abstract/8367702968354000PQ/1

Yu, Y.-M., Lee, Y.-J., Kim, J.-Y., Yoon, S.-B., & Shin, C.-S. (2016). Effects of forest therapy camp on quality of life and stress in postmenopausal women. *Forest Science and Technology*, *12*(3), 125–129. https://doi.org/10.1080/21580103.2015.1108248

# Social and health

Allen-Craig, S., & Ronalds, L. (2008). Preventing family and educational disconnection through wilderness-based therapy targeting youth at risk: [Youth homelessness in Australia.]. *ACHPER Australia Healthy Lifestyles Journal*, *55*(4), 5.

Bennett, J. L., Van Puymbroeck, M., Piatt, J. A., & Rydell, R. J. (2014). Veterans’ Perceptions of Benefits and Important Program Components of a Therapeutic Fly-Fishing Program. *Therapeutic Recreation Journal; Urbana*, *48*(2), 169–187.

Bettmann, J. (2007). Changes in adolescent attachment relationships as a response to wilderness treatment. *Journal of the American Psychoanalytic Association*, *55*(1), 259–265.

Bettmann, J. E., Russell, K. C., & Parry, K. J. (2013). How Substance Abuse Recovery Skills, Readiness to Change and Symptom Reduction Impact Change Processes in Wilderness Therapy Participants. *Journal of Child and Family Studies*, *22*(8), 1039–1050. https://doi.org/10.1007/s10826-012-9665-2

Bettmann, J. E., Tucker, A., Behrens, E., & Vanderloo, M. (2017). Changes in Late Adolescents and Young Adults’ Attachment, Separation, and Mental Health During Wilderness Therapy. *Journal of Child and Family Studies*, *26*(2), 511–522. https://doi.org/10.1007/s10826-016-0577-4

Bettmann, J., & Tucker, A. (2011). Shifts in Attachment Relationships: A Study of Adolescents in Wilderness Treatment. *Child & Youth Care Forum*, *40*(6), 499–519. https://doi.org/10.1007/s10566-011-9146-6

Bowen, D. J., Neill, J. T., & Crisp, S. J. R. (2016). Wilderness adventure therapy effects on the mental health of youth participants. *Evaluation and Program Planning*, *58*, 49–59. https://doi.org/10.1016/j.evalprogplan.2016.05.005

Brewer, J., & Sparkes, A. C. (2011). The meanings of outdoor physical activity for parentally bereaved young people in the United Kingdom: insights from an ethnographic study. *Journal of Adventure Education & Outdoor Learning*, *11*(2), 127–143. https://doi.org/10.1080/14729679.2011.633382

Brodin, J. (2011). Kan utomhuspedagogik stödja lärande och inkludering? *Socialmedicinsk tidskrift*, *88*(5), 445–458.

Burke, S. M., & Utley, A. (2013). Climbing towards recovery: investigating physically injured combat veterans’ psychosocial response to scaling Mt. Kilimanjaro. *Disability and Rehabilitation*, *35*(9), 732–739. https://doi.org/10.3109/09638288.2012.707743

Bæk, U. G., & Singhammer, J. (2016). *Ud i naturen ind i livet*. DGI Østjylland.

Bøgegaard, A., & Høegmark, S. (2015). *Vildmænd - Evaluering*. SVENDBORG KOMMUNE SUNDHEDSSEKRETARIATET.

Caulkins, M. C., White, D. D., & Russell, K. C. (2006). The Role of Physical Exercise in Wilderness Therapy for Troubled Adolescent Women. *Journal of Experiential Education*, *29*(1), 18–37.

Clark, J. P., Marmol, L. M., Cooley, R., & Gathercoal, K. (2004). The Effects of Wilderness Therapy on the Clinical Concerns (on Axes I, II, and IV) of Troubled Adolescents. *Journal of Experiential Education*, *27*(2), 213–232.

Cotton, S., & Butselaar, F. (2013). Outdoor adventure camps for people with mental illness. *Australasian Psychiatry*, *21*(4), 352–358.

Daniels, B. S. (2014). *Adolescent Subjective Experience of Wilderness Therapy: A Phenomenological Approach*. The Chicago School of Professional Psychology. Available at http://search.proquest.com/openview/de9bcc674120dabeb0bd98f2ad1586ff/1?pq-origsite=gscholar&cbl=18750&diss=y

DeMille, S. M. (2015). *Do therapeutic factors and client gender impact treatment outcomes for adolescents participating in outdoor behavioral healthcare treatment?* Capella University. Available at http://search.proquest.com/openview/3ebbe5591d40f57a10cafcc3b3b45413/1?pq-origsite=gscholar&cbl=18750&diss=y

DeMille, S. M., Comart, C., & Tucker, A. (2014). Body Composition Changes in an Outdoor Behavioral Healthcare Program. *Ecopsychology*, *6*(3), 174–182. https://doi.org/10.1089/eco.2014.0012

Donaldson, D. S. (2016). *Wounded veterans: Reintegration through adventure-based experience; A narrative inquiry*. California State University, Long Beach. Available at http://search.proquest.com/openview/9fed7d0208ad85f2be6a216b5fde46d7/1?pq-origsite=gscholar&cbl=18750&diss=y

Dorsch, T., Richards, K. A. R., Swain, J., & Maxey, M. (2016). The Effect of an Outdoor Recreation Program on Individuals With Disabilities and their Family Members: A Case Study. *Therapeutic Recreation Journal*, *50*(2). https://doi.org/10.18666/TRJ-2016-V50-I2-6527

Dustin, D., Bricker, N., Arave, J., Wall, W., & West, G. (2011). The Promise of River Running as a Therapeutic Medium for Veterans Coping with Post-Traumatic Stress Disorder. *Therapeutic Recreation Journal; Urbana*, *45*(4), 326–340.

Duvall, J., & Kaplan, R. (2014). Enhancing the well-being of veterans using extended group-based nature recreation experiences. *Journal of Rehabilitation Research and Development*, *51*(5), 685–696. https://doi.org/10.1682/JRRD.2013.08.0190

Ewert, A. (2014). Military veterans and the use of Adventure Education experiences in natural environments for therapeutic outcomes. *Ecopsychology*, *6*(3), 155–164.

Ewert, A., Frankel, J., Van Puymbroeck, M., & Luo, Y.-C. (2010). The impacts of participation in Outward Bound and military service personnel: The role of experiential training. *Journal of Experiential Education*, *32*(3), 313–316.

Ewert, A., Van Puymbroeck, M., Frankel, J., & Overholt, J. (2011). Adventure Education and the Returning Military Veteran: What Do We Know? *Journal of Experiential Education*, *33*(4), 365–369. https://doi.org/10.1177/105382591003300408

Fisker, H. J. (2010). Rehabiliterende friluftsprogram. Hentet 16. juni 2017, fra http://docplayer.dk/2931036-Rehabiliterende-friluftsprogram-fisker-hans-joergen.html

Frederiksen, R. B. (2012). *Rygestop i Vordingborg kommune*. Vordingborg Kommune.

Gerrard, L. L. (2012). *Women and their woods: Reflections on adolescence and wilderness therapy experiences* (Ph.D.). University of Kentucky, United States -- Kentucky. Available at http://search.proquest.com.ep.fjernadgang.kb.dk/docview/1506823082/abstract/3E0DCAA33C98450DPQ/1

Gillespie, E., & Allen-Craig, S. (2009). The Enhancement of Resilience via a Wilderness Therapy Program. A Preliminary Investigation. *Australian Journal of Outdoor Education*, *13*(1), 39–49.

Hansen, B. J. (2016). *Ud i naturen med misbrugere*. Sundhedsnetværket. Available at http://natur-vejleder.dk/wp-content/uploads/2016/03/januar_2016_misbrugere-1.pdf

Hansen, J., & Franzen, J. (2014). *Ældre og natur i Hjørring Kommune*. Hjørring Kommune: Sundhedsnetværket. Available at http://natur-vejleder.dk/wp-content/uploads/2016/03/november_2014_ldre_og_natur_i_hjrring.pdf

Harper, N., & Cooley, R. (2007). Parental Reports of Adolescent and Family Weil-Being following a Wilderness Therapy Intervention: An Exploratory Look at Systemic Change. *Journal of Experiential Education*, *29*(3), 393–396. https://doi.org/10.1177/105382590702900314

Harper, N. J. (2009). The relationship of therapeutic alliance to outcome in wilderness treatment. *Journal of Adventure Education and Outdoor Learning*, *9*(1), 45–59. https://doi.org/10.1080/14729670802460866

Harper, N. J., Russell, K. C., Cooley, R., & Cupples, J. (2007). Catherine Freer Wilderness Therapy Expeditions: An Exploratory Case Study of Adolescent Wilderness Therapy, Family Functioning, and the Maintenance of Change. *Child and Youth Care Forum*, *36*(2–3), 111. https://doi.org/10.1007/s10566-007-9035-1

Hillstead, D. (2004). *A qualitative study of families with an oppositional defiant adolescent male who is participating in a therapeutic wilderness program: Factors that lead to relationship disintegration and resolution*. Brigham Young University, United States -- Utah. Available at https://media-proquest-com.ep.fjernadgang.kb.dk/media/pq/classic/doc/766015981/fmt/ai/rep/SPDF?cit%3Aauth=Hillstead%2C+David+R.&cit%3Atitle=A+qualitative+study+of+families+with+an+oppositional+defiant+...&cit%3Apub=ProQuest+Dissertations+and+Theses&cit%3Avol=&cit%3Aiss=&cit%3Ap*g=*&cit%3Adate=2004&ic=true&cit%3Apro*d=*ProQuest+Dissertations+%26+Theses+Global&_a=ChgyMDE3MDcwNDExNDkzMjc2Mzo4MzUzMTUSBTk4MjQwGgpPTkVfU0VBUkNIIg4xMzAuMjI2LjIyOS4xNioFMTg3NTAyCTMwNTIxODY0NzoNRG9jdW1lbnRJbWFnZUIBMFIGT25saW5lWgJGVGIDUEZUagoyMDA0LzAxLzAxcgoyMDA0LzEyLzMxegCCASlQLTEwMDg3NDktMTA0MTYtQ1VTVE9NRVItMTAwMDAyMDUtMzk2NTY3NZIBBk9ubGluZcoBSE1vemlsbGEvNS4wIChXaW5kb3dzIE5UIDYuMTsgV09XNjQ7IHJ2OjU0LjApIEdlY2tvLzIwMTAwMTAxIEZpcmVmb3gvNTQuMNIBFkRpc3NlcnRhdGlvbnMgJiBUaGVzZXOaAgdQcmVQYWlkqgIoT1M6RU1TLVBkZkRvY1ZpZXdCYXNlLWdldE1lZGlhVXJsRm9ySXRlbbICJjIwMTcwNzA0MTE0OTMyNzYzOjgzNTMxNToxNDk5MTY5MzA4NTM0ugIpUC0xMDA4NzQ5LTEwNDE2LUNVU1RPTUVSLTEwMDAwMjA1LTM5NjU2NzXKAhNEaXNzZXJ0YXRpb24vVGhlc2lz0gIBWeICAPICAA%3D%3D&_s=BWR1S0T67bl4RHpqmDdCcwasQVY%3D

Hine, R., Wood, C., Barton, J., & Pretty, J. (2011). *The health and wellbeing effects of a walking and outdoor based therapy project—Report for Discovery Quest*. Colchester, UK: University of Essex. Available at https://static1.squarespace.com/static/56e9367020c64742fe062659/t/57038ac74d088e7781128e6f/1459849938024/Discovery+Quest+Report+2011+FINAL.pdf

Hoag, M. J., Massey, K. E., Roberts, S. D., & Logan, P. (2013). Efficacy of Wilderness Therapy for Young Adults: A First Look. *Residential Treatment For Children & Youth*, *30*(4), 294–305. https://doi.org/10.1080/0886571X.2013.852452

Ingman, B. C. (2013). *Rethinking the adventure education experience: An inquiry of meanings, culture and educational virtue*. University of Denver. Available at http://search.proquest.com/openview/fd38bccf2c07149023cae31fa9b8ef78/1?pq-origsite=gscholar&cbl=18750&diss=y

Jakubec, S., Den Hoed, D. C., & Ray, H. (2014). I can reinvent myself out here’: Experiences of nature inclusion and mental well-being. I *Environmental Contexts and Disability (Research in Social Science and Disability*. Emerald Group Publishing Limited. Available at http://www.emeraldinsight.com/doi/abs/10.1108/S1479-354720140000008012

Javorski, S. E., & Gass, M. A. (2013). 10-Year Incident Monitoring Trends in Outdoor Behavioral Healthcare: Lessons learned and future directions. *Journal of Therapeutic Schools & Programs*, *6*, 113–129.

Jensen, B. (2009). *”Tør du kysse skrubtudsen?”: Naturvejledning for brugere af psykiatrien*. Available at https://psyk-info.regionsyddanmark.dk/dwn433108

Jolander, N. (2015). Naturen som medspiller i frivilligt socialt arbejde. *Sundhedsnetværket*. Available at http://natur-vejleder.dk/wp-content/uploads/2016/03/juli_2015_familieoplevelsesklubber.pdf

Jones, C. D., Lowe, L. A., & Risler, E. A. (2004). The Effectiveness of Wilderness Adventure Therapy Programs for Young People Involved in the Juvenile Justice System. *Residential Treatment For Children & Youth*, *22*(2), 53–67. https://doi.org/10.1300/J007v22n02_04

Joyce, J., & Warren, A. (2016). A Case Study Exploring the Influence of a Gardening Therapy Group on Well-Being. *Occupational Therapy in Mental Health*, *32*(2), 203–215. https://doi.org/10.1080/0164212X.2015.1111184

Kishore, T. M., & Nagar, R. K. . (2008). Mountaineering expedition by persons with intellectual disability: Impact on behavior and temperament. *Journal of Intellectual Disabilities*, *12*(3), 183–189. https://doi.org/10.1177/1744629508095322

Kyriakopoulos, A. (2011). How individuals with self-reported anxiety and depression experienced a combination of individual counselling with an adventurous outdoor experience: A qualitative evaluation. *Counselling and Psychotherapy Research,* *11*(2), 120–128. https://doi.org/DOI: 10.1080/14733145.2010.485696

Mair, J., Hammerer-Lercher, A., Mittermayr, M., Klingler, A., Humpeler, E., Pachinger, O., & Schobersberger, W. (2008). 3-week hiking holidays at moderate altitude do not impair cardiac function in individuals with metabolic syndrome. *International Journal of Cardiology*, *123*(2), 186–188. https://doi.org/10.1016/j.ijcard.2006.11.127

Malmgren, M. (2013). *Red Barnets familieoplevelsesklubber - metoder og betydninger for børn og familier*. CASA. Available at https://redbarnet.dk/media/1249/evaluering-af-naturfaellesskabsprogrammet.pdf

Marti, S. (2007). *A wilderness therapy journey: An innovative psychotherapy and healing environment for adolescent girls*. Alliant International University, United States -- California. Available at https://media-proquest-com.ep.fjernadgang.kb.dk/media/pq/classic/doc/1313922841/fmt/ai/rep/SPDF?cit%3Aauth=Marti%2C+Stephanie+J.&cit%3Atitle=A+wilderness+therapy+journey%3A+An+innovative+psychotherapy+and+healing+environment+for+adolescent+girls&cit%3Apub=ProQuest+Dissertations+and+Theses&cit%3Avol=&cit%3Aiss=&cit%3Ap*g=*&cit%3Adate=2007&ic=true&cit%3Apro*d=*ProQuest+Dissertations+%26+Theses+Global&_a=ChgyMDE3MDcwNDExNDkzMjc2Mzo4MzUzMTUSBTk4MjQwGgpPTkVfU0VBUkNIIg4xMzAuMjI2LjIyOS4xNioFMTg3NTAyCTMwNDcwNDM5MzoNRG9jdW1lbnRJbWFnZUIBMFIGT25saW5lWgJGVGIDUEZUagoyMDA3LzAxLzAxcgoyMDA3LzEyLzMxegCCASlQLTEwMDg3NDktMTA0MTYtQ1VTVE9NRVItMTAwMDAyMDUtMzk2NTY3NZIBBk9ubGluZcoBSE1vemlsbGEvNS4wIChXaW5kb3dzIE5UIDYuMTsgV09XNjQ7IHJ2OjU0LjApIEdlY2tvLzIwMTAwMTAxIEZpcmVmb3gvNTQuMNIBFkRpc3NlcnRhdGlvbnMgJiBUaGVzZXOaAgdQcmVQYWlkqgIoT1M6RU1TLVBkZkRvY1ZpZXdCYXNlLWdldE1lZGlhVXJsRm9ySXRlbbICJjIwMTcwNzA0MTE0OTMyNzYzOjgzNTMxNToxNDk5MTY5NTg2MjE1ugIpUC0xMDA4NzQ5LTEwNDE2LUNVU1RPTUVSLTEwMDAwMjA1LTM5NjU2NzXKAhNEaXNzZXJ0YXRpb24vVGhlc2lz0gIBWeICAPICAA%3D%3D&_s=RkxK55PLpe4CRwaBHjQDYcQXlU0%3D

McArdle, K., Harrison, T., & Harrison, D. (2013). Does a nurturing approach that uses an outdoor play environment build resilience in children from a challenging background? *Journal of Adventure Education and Outdoor Learning*, *13*(3), 238–254. https://doi.org/10.1080/14729679.2013.776862

McCulloch, K., McLaughlin, P., Allison, P., Edwards, V., & Tett, L. (2010). Sail training as education: more than mere adventure. *Oxford Review of Education*, *36*(6), 661–676. https://doi.org/10.1080/03054985.2010.495466

Miramontes, L. P. (2007). *Exploring at-risk youths’ personal and social development during wilderness experiences*. University of Northern Colorado. Available at http://search.proquest.com/openview/6bf7121e1d907cdc1ace32b8bf7ba2f9/1?pq-origsite=gscholar&cbl=18750&diss=y

Morita, E., Imai, M., Okawa, M., Miyaura, T., & Miyazaki, S. (2011). A before and after comparison of the effects of forest walking on the sleep of a community-based sample of people with sleep complaints. *BioPsychoSocial Medicine*, *5*(1), 13–19. https://doi.org/10.1186/1751-0759-5-13

Mosher, J. R. (2015). *Family involvement and wilderness therapy outcomes: Perspectives from program directors*. Alliant International University. Available at http://search.proquest.com/openview/efc34c32253d3d52df626dd0b192ccaa/1?pq-origsite=gscholar&cbl=18750&diss=y

Nading, M. A., Lahmar, J. J., Frew, J. W., Ghionis, N., Hanley, M., Welch, A. K., & Murrell, D. F. (2009). A ski and adventure camp for young patients with severe forms of epidermolysis bullosa. *Journal of the American Academy of Dermatology*, *61*(3), 508–511. https://doi.org/10.1016/j.jaad.2008.12.004

Norton, C. L., & Watt, T. T. (2014). Exploring the impact of a wilderness-based positive youth development program for urban youth. *Journal of Experiential Education*, *37*(4), 335–350.

Ochiai, H., Ikei, H., Song, C., Kobayashi, M., Miura, T., Kagawa, T., … Miyazaki, Y. (2015). Physiological and Psychological Effects of a Forest Therapy Program on Middle-Aged Females. *International Journal of Environmental Research and Public Health*, *12*(12), 15222–15232. https://doi.org/10.3390/ijerph121214984

Ochiai, H., Ikei, H., Song, C., Kobayashi, M., Takamatsu, A., Miura, T., … Miyazaki, Y. (2015). Physiological and Psychological Effects of Forest Therapy on Middle-Aged Males with High-Normal Blood Pressure. *International Journal of Environmental Research and Public Health*, *12*(3), 2532–2542. https://doi.org/10.3390/ijerph120302532

Pedersen, G. L. (2015). Skovmeditation - en oplevelse for både krop og sjæl. Sundhedsnetværket. Available at http://natur-vejleder.dk/wp-content/uploads/2016/03/april_2015_skovmeditation.pdf

Poulsen, D. V., Stigsdotter, U. K., Djernis, D., & Sidenius, U. (2016). ‘Everything just seems much more right in nature’: How veterans with post-traumatic stress disorder experience nature-based activities in a forest therapy garden. *Health Psychology Open*, *3*(1), 205510291663709. https://doi.org/10.1177/2055102916637090

Pryor, A., Townsend, M., Maller, C., & Field, K. (2006). Health and well-being naturally: “contact with nature” in health promotion for targeted individuals, communities and populations. *Health Promotion Journal of Australia: Official Journal of Australian Association of Health Promotion Professionals*, *17*(2), 114–123.

Rasmussen, D., Hjarsen, T., & Eschen, K. (2014). *Du griner ligesom fra hele kroppen*. Available at http://docplayer.dk/17239439-Du-griner-ligesom-fra-hele-kroppen.html

Ritchie, S., Brinkman, D., Wabano, M. J., & Young, N. (2011). Reflections on Connecting through Outdoor Adventure. *Pathways: The Ontario Journal of Outdoor Education*, *23*(4), 4–9.

Ritchie, S. D., Wabano, M. J., Corbiere, R. G., Restoule, B. M., Russell, K. C., & Young, N. L. (2015). Connecting to the Good Life through outdoor adventure leadership experiences designed for Indigenous youth. *Journal of Adventure Education and Outdoor Learning*, *15*(4), 350–370. https://doi.org/10.1080/14729679.2015.1036455

Russell, K. C. (2005). Two Years Later: A Qualitative Assessment of Youth Well-Being and the Role of Aftercare in Outdoor Behavioral Healthcare Treatment. *Child and Youth Care Forum*, *34*(3), 209–239. https://doi.org/10.1007/s10566-005-3470-7

Russell, K. C. (2006). Evaluating the Effects of the Wendigo Lake Expedition Program on Young Offenders. *Youth Violence and Juvenile Justice*, *4*(2), 185–203. https://doi.org/10.1177/1541204006286317

Russell, K. C., & Harper, N. J. (2006). Incident monitoring in outdoor behavioral healthcare programs: A four-year summary of restraint, runaway, injury, and illness rates. *Journal of Therapeutic Schools & Programs*, *1*(1), 70–91.

Sandholdt, C., & Keller, M. (2014). *RUN - relationer, udvikling og natur. Et aktivitets- og mentorprojekt*. Kriminalforsorgen.

Schrader, L. (2012). *A grounded theory investigation of at-risk youth who participate in outdoor adventure programs*. Capella University, United States -- Minnesota. Available at https://media-proquest-com.ep.fjernadgang.kb.dk/media/pq/classic/doc/2669992101/fmt/ai/rep/NPDF?cit%3Aauth=Schrader%2C+Lisa+L.&cit%3Atitle=A+grounded+theory+investigation+of+at-risk+youth+who+participate+in+outdoor+adventure+programs&cit%3Apub=ProQuest+Dissertations+and+Theses&cit%3Avol=&cit%3Aiss=&cit%3Ap*g=*&cit%3Adate=2012&ic=true&cit%3Apro*d=*ProQuest&_a=ChgyMDE3MDcwNDExNDkzMjc2Mzo4MzUzMTUSBTk4MjQwGgpPTkVfU0VBUkNIIg4xMzAuMjI2LjIyOS4xNioFMTg3NTAyCjEwMTYxNjIzNzM6DURvY3VtZW50SW1hZ2VCATBSBk9ubGluZVoCRlRiA1BGVGoKMjAxMi8wMS8wMXIKMjAxMi8xMi8zMXoAggEpUC0xMDA3MTA2LTEwNDE2LUNVU1RPTUVSLTEwMDAwMjU1LTUwNDU4NzWSAQZPbmxpbmXKAUhNb3ppbGxhLzUuMCAoV2luZG93cyBOVCA2LjE7IFdPVzY0OyBydjo1NC4wKSBHZWNrby8yMDEwMDEwMSBGaXJlZm94LzU0LjDSARZEaXNzZXJ0YXRpb25zICYgVGhlc2VzmgIHUHJlUGFpZKoCKE9TOkVNUy1QZGZEb2NWaWV3QmFzZS1nZXRNZWRpYVVybEZvckl0ZW2yAiYyMDE3MDcwNDExNDkzMjc2Mzo4MzUzMTU6MTQ5OTE2OTEyMzIyN7oCKVAtMTAwNzEwNi0xMDQxNi1DVVNUT01FUi0xMDAwMDI1NS01MDQ1ODc1ygITRGlzc2VydGF0aW9uL1RoZXNpc9ICAVniAgDyAgA%3D&_s=ki32GiXvKgRkO0U4omEIRYoZHb4%3D

Somervell, J., & Lambie, I. (2009). Wilderness therapy within an adolescent sexual offender treatment programme: A qualitative study. *Journal of Sexual Aggression*, *15*(2), 161–177. https://doi.org/10.1080/13552600902823055

Stevens, B., Kagan, S., Yamada, J., Epstein, I., Beamer, M., Bilodeau, M., & Baruchel, S. (2004). Adventure therapy for adolescents with cancer. *Pediatric Blood & Cancer*, *43*(3), 278–284. https://doi.org/10.1002/pbc.20060

Sutherland, S., & Stroot, S. A. (2009). Brad’s Story: Exploration of an Inclusive Adventure Education Experience. *Therapeutic Recreation Journal; Urbana*, *43*(3), 27–39.

Swane, C. (2005, juli 15). Grøn omsorg styrker mentale ressourcer | Sygeplejersken, DSR. *DSR*, (14). Available at https://dsr.dk/sygeplejersken/arkiv/sy-nr-2005-14/groen-omsorg-styrker-mentale-ressourcer

Swane, C. E. (2004). *Skovdagcentret Eghjorten i Hillerød : naturens betydning for mennesker med demens i relation til eksisterende forskning* (Bd. 2004). Hillerød Kommune.

Tian, Y., He, Z., Xu, C., Huang, C., Lee, J.-H., Li, R., … Mc Naughton, L. (2015). Energy Expenditure and Fitness Response Following Once Weekly Hill Climbing at Low Altitude. *International Journal of Sports Medicine*, *36*(05), 357–364. https://doi.org/10.1055/s-0034-1395520

Tonnesen, B. (2015). *Naturvejlederløntilskud Årsrapport 2015*. Gråsten: Danske Diakonhjem Dalsmark. Available at http://www.fr-tips.dk/userfiles/d27c6ace56a70b74f80026e51c2b137e.pdf

Tucker, A., Norton, C. L., DeMille, S. M., & Hobson, J. (2016). The Impact of Wilderness Therapy. *Journal of Experiential Education*, *39*(1), 15–30. https://doi.org/10.1177/1053825915607536

Ungar, M., Dumond, C., & Mcdonald, W. (2005). Risk, Resilience and Outdoor Programmes for At-risk Children. *Journal of Social Work*, *5*(3), 319–338. https://doi.org/10.1177/1468017305058938

Vallet, C., André, N., Gentet, J.-C., Verschuur, A., Michel, G., Sotteau, F., … Grélot, L. (2015). Pilot evaluation of physical and psychological effects of a physical trek programme including a dog sledding expedition in children and teenagers with cancer. *ecancermedicalscience*, *9*. https://doi.org/10.3332/ecancer.2015.558

Vikene, O. L. (2012). Psykisk helse og opplevelsen av deltakelse i friluftsliv - PDF. *Tidsskrift for Psykisk Helsearbeid*, *9*(4). Available at http://docplayer.me/6790528-Psykisk-helse-og-opplevelsen-av-deltakelse-i-friluftsliv.html

Walker, A. J., Onus, M., Doyle, M., Clare, J., & McCarthy, K. (2005). Cognitive rehabilitation after severe traumatic brain injury: A pilot programme of goal planning and outdoor adventure course participation. *Brain Injury*, *19*(14), 1237–1241. https://doi.org/10.1080/02699050500309411

Wilson, J. F., & Christensen, K. M. (2012). The Relationship Between Outdoor Recreation and Depression Among Individuals With Disabilities. *Journal of Leisure Research; Urbana*, *44*(4), 486–506.

# Education and daycare

Amanda L. McGowan. (2016). Impact of One-Semester Outdoor Education Programs on Adolescent Perceptions of Self-Authorship. *Journal of Experiential Education*, *39*(4), 386–411. https://doi.org/DOI: 10.1177/1053825916668902

Asfeldt, M., & Hvenegaard, G. (2014). Perceived learning, critical elements and lasting impacts on university-based wilderness educational expeditions. *Journal of Adventure Education and Outdoor Learning*, *14*(2), 132–152. https://doi.org/10.1080/14729679.2013.789350

Austin, M. L., Martin, B., Mittelstaedt, R., Schanning, K., & Ogle, D. (2009). Outdoor orientation program effects: Sense of place and social benefits. *Journal of Experiential Education*, *31*(3), 435–439.

Beames, S. (2005). Expeditions and the social construction of the self. *Australian Journal of Outdoor Education*, *9*(1), 14–22.

Bell, B. J. (2006). Wilderness orientation: Exploring the relationship between college preorientation programs and social support. *Journal of Experiential Education*, *29*(2), 145–167.

Bettmann, J., & Tucker, A. (2011). Shifts in Attachment Relationships: A Study of Adolescents in Wilderness Treatment. *Child & Youth Care Forum*, *40*(6), 499–519. https://doi.org/10.1007/s10566-011-9146-6

Bjørgen, K. (2015). Children’s Well-being and Involvement in Physically Active Outdoors Play in a Norwegian Kindergarten: Playful Sharing of Physical Experiences. *Child Care in Practice*, *21*(4), 305–323. https://doi.org/10.1080/13575279.2015.1051512

Bobilya, A. J., Kalisch, K., & Daniel, B. (2011). An Investigation of the Outward Bound Final Expedition. *Journal of Experiential Education*, *33*(4), 356–359. https://doi.org/10.5193/JEE33.4.356

Bobilya, A. J., Kalisch, K. R., & Daniel, B. (2014). Participants’ perceptions of their outward bound final expedition and the relationship to instructor supervisory position. *Journal of Experiential Education*, *37*(4), 397–414.

Boettcher, M. L., & Gansemer-Topf, A. M. (2015). Examining Leadership Development through Student Leader Outdoor Recreation Training. *Recreational Sports Journal*, *39*(1), 49–58. https://doi.org/10.1123/rsj.2014-0034

Bowen, D., Neill, J., R. Williams, I., Mak, A., Allen, N., & Olsson, C. (2016). A Profile of Outdoor Adventure Interventions for Young People in Australia. *Journal of Outdoor Recreation, Education, and Leadership*, *8*. https://doi.org/10.18666/JOREL-2016-V8-I1-7281

Breunig, M., O’Connell, T., Todd, S., Young, A., Anderson, L., & Anderson, D. (2008). Psychological sense of community and group cohesion on wilderness trips. *Journal of experiential education*, *30*(3), 258–261.

Cheung, A. C. K. (2011). SEER 2010 ABSTRACT: Spiritual Development of Adolescents in Adventure-Based Programs in Hong Kong. *Journal of Experiential Education*, *33*(4), 411–415. https://doi.org/10.5193/JEE33.4.411

Cooley, S. (2015, maj 31). *Developing groupwork through outdoor adventure education: A systematic evaluation of learning and transfer in higher education*. University of Birmingham, UK. https://doi.org/10.13140/RG.2.1.3836.6164

Dahl, L. (2015). Ulykker i friluftsliv på videregående skole. I *Rapport fra konferansen* (s. 108–115). Lillehammer, Norge: Norsk Friluftsliv.

D’Amato, L. G., & Krasny, M. E. (2011). Outdoor Adventure Education: Applying Transformative Learning Theory to Understanding Instrumental Learning and Personal Growth in Environmental Education. *The Journal of Environmental Education*, *42*(4), 237–254. https://doi.org/10.1080/00958964.2011.581313

Davidson, C. (2016). *Building character through adventure education: A study of levels of grit and resilience in outward bound students*. Indiana University. Available at http://search.proquest.com/openview/70ba3026e4611fb0cf348065f7208f3f/1?pq-origsite=gscholar&cbl=18750&diss=y

Edlund, C. (2011). *Student Perceptions of Outdoor Educational Experiences*. Walden University. Available at http://search.proquest.com/openview/a407944012917d84d0221d9afcefb3b9/1?pq-origsite=gscholar&cbl=18750&diss=y

Ewert, A., & Yoshino, A. (2008). A Preliminary Exploration of the Influence of Short-Term Adventure-Based Expeditions on Levels of Resilience. *Journal of Experiential Education*, *30*(3), 262–266.

Ferraro, F. M. (2015). Enhancement of Convergent Creativity Following a Multiday Wilderness Experience. *Ecopsychology*, *7*(1), 7–11. https://doi.org/10.1089/eco.2014.0043

Fjørtoft, I., & Larsen, R. (2005). HØY PULS I UTESKOLEN! En pilotstudie av elever i klasse i Ute-aktiviteter. Hentet 9. november 2017, fra http://docplayer.me/4509859-Hoy-puls-i-uteskolen-en-pilotstudie-av-elever-i-1-4-klasse-i-ute-aktiviteter.html

Gaudio, G., Greenwald, P.G., & Holton. (2010). Injury and illness in college outdoor education. *Wilderness & Environmental Medicine*, *21*, 363–370.

Goldenberg, M., Cummings, J., & Pronsolino, D. (2008). A means-end study of outcome differences of females and males associated with Outward Bound and National Outdoor Leadership School. *Research in Outdoor Education*, *9*, 16.

Goldenberg, M., & Pronsolino, D. (2008). A Means-End Investigation of Outcomes Associated with Outward Bound and NOLS Programs. *Journal of Experiential Education*, *30*(3), 271–276.

Goldenberg, M., & Soule, K. E. (2015). A four-year follow-up of means-end outcomes from outdoor adventure programs. *Journal of Adventure Education and Outdoor Learning*, *15*(4), 284–295. https://doi.org/10.1080/14729679.2014.970343

Grønningsæter, I., Hallås, O., Kristiansen, T., & Nævdal, F. (2007). Fysisk aktivitet hos 11 – 12-åringar i skulen. *Tidsskriftet Den Norske Legeforeng*. Available at http://tidsskriftet.no/sites/default/files/pdf2007--2927-9.pdf

Harun, M. T., & Salamuddin, N. (2013). Applying Elements of Outdoor Education in Teacher Education Innovation. *Asian Social Science*, *9*(16), 15. https://doi.org/10.5539/ass.v9n16p15

Hayhurst, J., Hunter, J. A., Kafka, S., & Boyes, M. (2015). Enhancing resilience in youth through a 10-day developmental voyage. *Journal of Adventure Education and Outdoor Learning*, *15*(1), 40–52. https://doi.org/10.1080/14729679.2013.843143

Henstock, M., Barker, K., & Knijnik, J. (2013). 2, 6, Heave! Sail Training’s Influence on the Development of Self-Concept and Social Networks and Their Impact on Engagement with Learning and Education. A Pilot Study. *Australian Journal of Outdoor Education*, *17*(1), 32–46.

Hinds, J. (2011). Woodland Adventure for Marginalized Adolescents: Environmental Attitudes, Identity and Competence. *Applied Environmental Education & Communication*, *10*(4), 228–237. https://doi.org/10.1080/1533015X.2011.669689

Holloway, J. A., Murray, J., Okada, R., & Emmons, A. L. (2014). Ecopsychology and Relationship Competency: The Empowerment of Women Graduate Students Through Nature Experiences. *Women & Therapy*, *37*(1–2), 141–154. https://doi.org/10.1080/02703149.2014.850343

Hovey, K., Foland, J., Foley, J. T., Kniffin, M., & Bailey, J. (2016). Predictors of Change in Body Image in Female Participants of an Outdoor Education Program. *Journal of Outdoor Recreation, Education, and Leadership*, *8*(2), 200–208. https://doi.org/10.18666/JOREL-2016-V8-I2-7698

Humberstone, B., & Stan, I. (2009). Well-being and outdoor pedagogies in primary schooling: The nexus of well-being and safety. *Journal of Outdoor and Environmental Education*, *13*(2), 24.

Ingman, B. C. (2013). *Rethinking the adventure education experience: An inquiry of meanings, culture and educational virtue*. University of Denver. Available at http://search.proquest.com/openview/fd38bccf2c07149023cae31fa9b8ef78/1?pq-origsite=gscholar&cbl=18750&diss=y

Jacobsen, C. (2005). Forældres holdning til og erfaring med naturklasseprojektet. I *Udeundervisning i folkeskolen: et casestudie om en naturklasse på Rødkilde Skole og virkningerne af en ugentlig obligatorisk naturdag på yngste klassetrin i perioden 2000-2003* (s. 257–270). Museum Tusculanum.

Jensen, M. (2015). Friluftsliv som pædagogisk metode - Studie af friluftsliv anvendt i pædagogisk arbejde med socialt udsatte unge i alderen 12 -21 år. Friluftsrådet.

Johnson, J., & Chin, J. W. (2016). Hazing rites/rights: using outdoor- and adventure education-based orientation to effect positive change for first-year athletes. *Journal of Adventure Education and Outdoor Learning*, *16*(1), 16–30. https://doi.org/10.1080/14729679.2015.1050681

Jones, J. J., & Hinton, J. L. (2007). Study of self-efficacy in a freshman wilderness experience program: Measuring general versus specific gains. *Journal of Experiential Education*, *29*(3), 382–385.

Jostad, J. (2015). *A dynamical systems theory examination of social connections in outdoor recreation programs*. The University of Utah, USA. Available at http://search.proquest.com/openview/073c3b531b703135cd73fe81bb1cd779/1?pq-origsite=gscholar&cbl=18750&diss=y

Jostad, J., Paisley, K., & Gookin, J. (2012). Wilderness-based semester learning: Understanding the NOLS experience. *Journal of Outdoor Recreation, Education, and Leadership*, *4*(1), 16–26.

Jostad, J., Sibthorp, J., & Paisley, K. (2013). Understanding groups in outdoor adventure education through social network analysis. *Australian Journal of Outdoor Education*, *17*(1), 17.

Juriza, I., Ruzanna, Z., Harlina, R., Ma, F., Radniwan, M., Razif, M., … S, L. (2011). Outdoor Camps Experiential Learning Activities for Teamwork and Leadership among Medical Students. *Procedia - Social and Behavioral Sciences*, *18*(Supplement C), 622–625. https://doi.org/10.1016/j.sbspro.2011.05.091

Jørgensen, K.-A. (2014). What is going on out there?-What does it mean for children’s experiences when the kindergarten is moving their everyday activities into the nature-landscapes and its places. Available at https://130.241.16.4/handle/2077/37251

K. Barr-Wilson, S., & Roberts, N. (2016). Adolescent Girls and Body Image: Influence of Outdoor Adventure on Healthy Living. *Journal of Outdoor Recreation, Education, and Leadership*, *8*, 148–164. https://doi.org/10.18666/JOREL-2016-V8-I2-7693

Karppinen, S. J. A. (2012). Outdoor adventure education in a formal education curriculum in Finland: action research application. *Journal of Adventure Education and Outdoor Learning*, *12*(1), 41–62. https://doi.org/10.1080/14729679.2011.569186

Kass, D., & Grandzol, C. (2012). Evaluating the Value-Added Impact of Outdoor Management Training for Leadership Development in an MBA Program. *Journal of Experiential Education*, *35*(3), 429–446. https://doi.org/10.5193/JEE35.3.429

Larry Mason Peebles,. (2006, december). *IMPROVING SELF-EFFICACY IN COLLEGE STUDENTS: A MODIFIED ADVENTURE THERAPY PROGRAM* (Dissertation Prepared for the Degree of DOCTOR OF EDUCATION). UNIVERSITY OF NORTH TEXAS. Available at https://digital.library.unt.edu/ark:/67531/metadc5443/

Liang, G., & Bo, N. (2009). The pretest-posttest design and measurement of outward-bound-type program effects on personal development. *Procedia Earth and Planetary Science*, *1*(1), 1717–1722. https://doi.org/10.1016/j.proeps.2009.09.263

Lien, M., & Goldenberg, M. (2012). Outcomes of a College Wilderness Orientation Program. *Journal of Experiential Education*, *35*(1), 253–271. https://doi.org/10.1177/105382591203500104

Løvoll, H. S., Vittersø, J., & Wold, B. (2016). Experiencing the outdoors: peak episodes are interesting but the memories are pleasant. *Journal of Adventure Education and Outdoor Learning*, 1–16. https://doi.org/10.1080/14729679.2015.1122541

MacQuarrie, S., Nugent, C., & Warden, C. (2015). Learning with nature and learning from others: nature as setting and resource for early childhood education. *Journal of Adventure Education and Outdoor Learning*, *15*(1), 1–23. https://doi.org/10.1080/14729679.2013.841095

Massey, S. (2005). The benefits of a forest school experience for children in their early years. *nfer*, 27–35.

McClain, C., & Vandermaas-Peeler, M. (2016). Social contexts of development in natural outdoor environments: children’s motor activities, personal challenges and peer interactions at the river and the creek. *Journal of Adventure Education and Outdoor Learning*, *16*(1), 31–48. https://doi.org/10.1080/14729679.2015.1050682

McIntosh, S. E., Guercio, B., Tabin, G. C., Leemon, D., & Schimelpfenig, T. (2011). Ultraviolet Keratitis Among Mountaineers and Outdoor Recreationalists. *Wilderness & Environmental Medicine; New York*, *22*(2), 144–147.

McIntosh, S. E., Leemon, D., Visitacion, J., Schimelpfenig, T., & Fosnocht, D. (2007). Medical Incidents and Evacuations on Wilderness Expeditions. *Wilderness & Environmental Medicine; New York*, *18*(4), 298–304.

Mirkin, B. J. (2013). *Examining social climate and youth social goals on extended wilderness courses: A path toward improving participant experiences*. University of New Hampshire. Available at http://search.proquest.com/openview/724d0a3e5b358355827594d8d8c10ad2/1?pq-origsite=gscholar&cbl=18750&diss=y

Mirkin, B. J., & Middleton, M. J. (2014). The social climate and peer interaction on outdoor courses. *Journal of Experiential Education*, *37*(3), 232–247.

Murray, R., & O’Brien, L. (2005). *Such enthusiasm–a joy to see An evaluation of Forest School in England*. Available at http://forums.forestry.gov.uk/pdf/ForestSchoolEnglandReport.pdf/$FILE/ForestSchoolEnglandReport.pdf

Mutz, M., & Müller, J. (2016). Mental health benefits of outdoor adventures: Results from two pilot studies. *Journal of Adolescence*, *49*, 105–114. https://doi.org/10.1016/j.adolescence.2016.03.009

Mygind, E. (2005a). Elevudsagn om naturens rum og klasseværelset. I *Udeundervisning i folkeskolen: Et casestudie om en naturklasse på Rødkilde Skole og virkningerne af en ugentlig obligatorisk naturdag på yngste klassetrin i perioden 2000-2003*. Museum Tusculanum.

Mygind, E. (2005b). *Udeundervisning i folkeskolen: et casestudie om en naturklasse på Rødkilde Skole og virkningerne af en ugentlig obligatorisk naturdag på yngste klassetrin i perioden 2000-2003*. Museum Tusculanum Press.

Mygind, E. (2007). A comparison between children’s physical activity levels at school and learning in an outdoor environment. *Journal of Adventure Education and Outdoor Learning*, *7*(2), 161–176. https://doi.org/10.1080/14729670701717580

Mygind, E. (2009). A comparison of childrens’ statements about social relations and teaching in the classroom and in the outdoor environment. *Journal of Adventure Education and Outdoor Learning*, *9*(2), 151–169. https://doi.org/10.1080/14729670902860809

Nawaz, H., & Blackwell, S. (2014). Perceptions about Forest Schools: Encouraging and Promoting Archimedes Forest Schools. *Educational Research and Reviews*, *9*(15), 498–503.

Niell, J. T. (2008). *Enhancing life effectiveness: the impact of outdoor education programs*. University of Western Sydney, Australien. Available at http://asa.scitation.org/doi/abs/10.1121/1.1907526

O’Brien, L. (2009). Learning outdoors: the Forest School approach. *Education 3-13*, *37*(1), 45–60. https://doi.org/10.1080/03004270802291798

O’Brien, L., Burls, A., Townsend, M., & Ebden, M. (2011). Volunteering in nature as a way of enabling people to reintegrate into society. *Perspectives in Public Health; London*, *131*(2), 71–81.

O’Brien, L., Great Britain, & Forestry Commission. (2006). *A marvellous opportunity for children to learn: a participatory evaluation of forest school in England and Wales*. Farnham, Surrey: Forest Research.

O’Brien, L., & Murray, R. (2007). Forest School and its impacts on young children: Case studies in Britain. *Urban Forestry & Urban Greening*, *6*(4), 249–265. https://doi.org/10.1016/j.ufug.2007.03.006

Ooko, S. W., Muthomi, H., & Odhiambo, G. (2015). Impact of outdoor adventure education on Kenyan youth, in peace building. *World Leisure Journal,* *57*(4), 297–305. https://doi.org/DOI: 10.1080/16078055.2015.1081270

O’Shea, K. (2008). Exploring the benefits of an outdoor adventure program for improving self-Esteem and self-Efficacy and reducing problem behaviors in adolescent girls. Available at http://scholarworks.umt.edu/etd/419/

Overholt, J. R. (2013). *Exploring familial relationship growth and negotiation: A case study of Outward Bound family courses*. Citeseer. Available at http://citeseerx.ist.psu.edu/viewdoc/download?doi=10.1.1.819.4964&rep=rep1&type=pdf

Philipson, B. S. (2013). *How a summer camp counselor-in-training program may foster resilience and self-efficacy in adolescent boys* (Ph.D.). Tulane University, United States -- Louisiana. Available at https://search-proquest-com.ep.fjernadgang.kb.dk/pqdtglobal/docview/1347387047/abstract/3AF382C333854977PQ/1

Ray, S. (2007). *The Experience of Adolescent Girls Participating in an Adventure Therapy Program: A Qualitative Study*.

Richmond, D. J. (2016, august). *Developing noncognitive factors through outdoor adventure education: experiences that complement classroom learning :: IR - Theses & Dissertations 3*. University of Utah, USA. Available at http://cdmbuntu.lib.utah.edu/cdm/ref/collection/etd3/id/4270, http://cdmbuntu.lib.utah.edu/cdm/ref/collection/etd3/id/4270

Rude, W. J. (2015). *Outdoor adventure education and thriving: The relationship between outdoor orientation and college student well-being*. Azusa Pacific University. Available at http://search.proquest.com/openview/ac9041da5cc4f33f3b56dd91f711ea73/1?pq-origsite=gscholar&cbl=18750&diss=y

Russell, K. C., & Harper, N. J. (2006). Incident monitoring in outdoor behavioral healthcare programs: A four-year summary of restraint, runaway, injury, and illness rates. *Journal of Therapeutic Schools & Programs*, *1*(1), 70–91.

Sammet, K. (2010). Relationships Matter: Adolescent Girls and Relational Development in Adventure Education. *Journal of Experiential Education*, *33*(2), 151–165. https://doi.org/10.5193/JEE33.2.151

Sanderud, J. R., & Gurholt, K. P. (2015). Nysgjerrig lek: Utforskende dannelse. I *Rapport fra konferansen* (s. 218–224). Lillehammer, Norge: Norsk Friluftsliv.

Scrutton, R. A. (2015). Outdoor adventure education for children in Scotland: quantifying the benefits. *Journal of Adventure Education and Outdoor Learning*, *15*(2), 123–137. https://doi.org/10.1080/14729679.2013.867813

Shirilla, P. (2009). Adventure-Based Programming and Social Skill Development in the Lives of Diverse Youth: Perspectives from Two Research Projects. *Journal of Experiential Education*, *31*(3), 410–414. https://doi.org/10.1177/105382590803100310

Sibthorp, J., Collins, R., Rathunde, K., Paisley, K., Schumann, S., Pohja, M., … Baynes, S. (2015). Fostering Experiential Self-Regulation Through Outdoor Adventure Education. *Journal of Experiential Education*, *38*(1), 26–40. https://doi.org/10.1177/1053825913516735

Sibthorp, J., Furman, N., Paisley, K., Gookin, J., & Schumann, S. (2009). Learning Transferable Lessons from Adventure Education: Qualitative Results from a NOLS Transfer Survey. Præsenteret ved Association of Outdoor Recreation & Education Conference Proceed. Available at http://connection.ebscohost.com/c/articles/58518551/learning-transferable-lessons-from-adventure-education-qualitative-results-from-nols-transfer-survey

Sibthorp, J., Paisley, K., & Gookin, J. (2007). Exploring Participant Development Through Adventure-Based Programming: A Model from the National Outdoor Leadership School. *Leisure Sciences*, *29*(1), 1–18. https://doi.org/10.1080/01490400600851346

Sibthorp, J., Paisley, K., Gookin, J., & Furman, N. (2008). The pedagogic value of student autonomy in adventure education. *Journal of Experiential Education*, *31*(2), 136–151.

Slade, M., Lowery, C., & Bland, K. (2013). Evaluating the impact of forest schools: A collaboration between a university and a primary school. *Support for Learning*, *28*(2), 66–72.

Smith, E. F., Steel, G., & Gidlow, B. (2010). The Temporary Community: Student Experiences of School-Based Outdoor Education Programmes. *Journal of Experiential Education*, *33*(2), 136–150. https://doi.org/10.5193/JEE33.2.136

Sproule, J., Martindale, R., Wang, J., Allison, P., Nash, C., & Gray, S. (2013). Investigating the experience of outdoor and adventurous project work in an educational setting using a self-determination framework. *European Physical Education Review*, *19*(3), 315–328. https://doi.org/10.1177/1356336X13495629

Stan, I. (2008). *Group Interaction in the “Outdoor Classroom”: the Process of Learning in Outdoor Education*. Brunel University.

Stenberg, K. (2007). Free Life: A Report from the. *Pathways: The Ontario Journal of Outdoor Education*, *19*(3), 29–33.

Stoddart, F. (2004). Developing social capital through outdoor education in Cumbria: A case study. *Connections and Disconnections: Examining the reality and rhetoric. International perspectives on outdoor education theory and practice. Victoria, Australia, La Trobe University*. Available at http://www.latrobe.edu.au/education/downloads/2004_conference_stoddart.pdf

Sølvik, R. M. (2013). Friluftsliv som sosialt l\a eringslandskap for ungdom i risiko. Eit fenomenologisk-inspirert kasusstudium. Available at https://www.duo.uio.no/handle/10852/41175

Tardona, D. (2014). A Program Encouraging Healthy Behavior, Nature Exploration, and Recreation through History in an Urban National Park Unit. *Journal of Park and Recreation Administration*, *32*, 73–82.

Veitch, K. B., David Crawford, G. A., & Jo Salmon. (2013). Is park visitation associated with leisure-time and transportation physical activity? *Preventive Medicin*, *57*(5), 732–734. https://doi.org/DOI: 10.1016/j.ypmed.2013.08.001

Vella, E. J., Milligan, B., & Bennett, J. L. (2013). Participation in Outdoor Recreation Program Predicts Improved Psychosocial Well-Being Among Veterans With Post-Traumatic Stress Disorder: A Pilot Study. *Military Medicine*, *178*(3), 254–260. https://doi.org/10.7205/MILMED-D-12-00308

Vigsø, B., & Nielsen, V. (2006). *Børn og Udeliv*. Danmark: CVU Vest Press.

Wang, C. J., Woon-Chia, L., & Kahlid, A. (2006). Effects of a five-day Outward Bound course on female students in Singapore. *Journal of Outdoor and Environmental Education*, *10*(2), 20.

Ward, W., & Hobbs, W. (2006). Changes in Perceptions of Fear in a Short-Term, College Outdoor Adventure Program. *Journal of Experiential Education*, *28*(3), 274–278. https://doi.org/10.1177/105382590602800314
