# Supplementary material for: Immersive Nature-Experiences as Health Promotion Interventions for Healthy, Vulnerable, and Sick Populations? A Systematic Review and Appraisal of Controlled Studies
Source: Front Psychol. 2019 May 3;10:943. doi: 10.3389/fpsyg.2019.00943 (PMC6509207; doi:10.3389/fpsyg.2019.00943)
Supplement: Supplementary file 4 [file Table_4.docx]

Supplementary material D: All outcomes across sectors and health domains

|  |  |  | **Recreation** | | | |  | **Health & Social** | | | |  | **Education** | | | |  | **Total** | | | |
| --- | --- | --- | --- | --- | --- | --- | --- | --- | --- | --- | --- | --- | --- | --- | --- | --- | --- | --- | --- | --- | --- |
|  |  |  | + | +/ | / | - |  | + | +/ | / | - |  | + | +/ | / | - |  | + | +/ | / | - |
| **Mental health** | | | **57** | **22** | **31** | **1** |  | **5-** | **5** | **28** | **1** |  | **37** | **5** | **12** | **1** |  | **144** | **32** | **71** | **3** |
|  | Psychological wellbeing | | 22 | 13 | 11 | - |  | 26 | 2 | 16 | 1 |  | 5 | - | - | 1 |  | 53 | 15 | 27 | 2 |
|  |  | Positive affect | 9 | - | 1 | - |  | 2 | 1 | - | - |  | - | - | - | - |  | 11 | 1 | 1 | - |
|  |  | Negative affect | 1 | - | 7 | - |  | - | - | 1 | - |  | - | - | - | - |  | 1 | - | 8 | - |
|  |  | Stress | 3 | - | 1 | - |  | - | - | 2 | - |  | 1 | - | - | - |  | 4 | - | 3 | - |
|  |  | Mood states | 1 | 13 | - | - |  | 6 | - | 1 | - |  | 2 | - | - | - |  | 9 | 13 | 1 | - |
|  |  | Depression/depressive state/hopelessness | - | - | - | - |  | 7 | - | 4 | - |  | - | - | - | - |  | 7 | - | 4 | - |
|  |  | Fatigue | - | - | - | - |  | 1 | - | - | 1 |  | - | - | - | - |  | 1 | - | - | 1 |
|  |  | Level of burnout | - | - | - | - |  | 1 | - | - | - |  | - | - | - | - |  | 1 | - | - | - |
|  |  | Anxiety | 2 | - | - | - |  | 1 | - | 4 | - |  | - | - | - | - |  | 3 | - | 4 | - |
|  |  | Thoughts of suicide | - | - | - | - |  | 1 | - | - | - |  | - | - | - | - |  | 1 | - | - | - |
|  |  | Rumination/negative memories and feelings towards self | 2 | - | - | - |  | - | - | 1 | - |  | - | - | - | - |  | 2 | - | 1 | - |
|  |  | Vitality | 3 | - | - | - |  | - | - | - | - |  | - | - | - | - |  | 3 | - | - | - |
|  |  | Quality of life/general wellbeing | 1 | - | 1 | - |  | 5 | 1 | - | - |  | 2 | - | - | - |  | 8 | 1 | 1 | - |
|  |  | Life purpose | - | - | - | - |  | - | - | - | - |  | - | - | - | 1 |  | - | - | - | 1 |
|  |  | Internalizing and externalizing behaviors | - | - | 1 | - |  | - | - | 1 | - |  | - | - | - | - |  | - | - | 2 | - |
|  |  | Generalized symptoms of psychopathology | - | - | - | - |  | - | - | 1 | - |  | - | - | - | - |  | - | - | 1 | - |
|  |  | Schizophrenia symptom severity | - | - | - | - |  | - | - | 1 | - |  | - | - | - | - |  | - | - | 1 | - |
|  |  | PTSD symptoms | - | - | - | - |  | 1 | - | - | - |  | - | - | - | - |  | 1 | - | - | - |
|  |  | *Mental functioning (e.g., anxiety and locus of control)** | | | | | | 1 | - | - | - |  | - | - | - | - |  | 1 | - | - | - |
|  | Psychophysiological stress-indicators | | 23 | 9 | 12 | - |  | 5 | - | - | - |  | 1 | - | - | - |  | 29 | 9 | 12 | - |
|  |  | Heart rate variability | 3 | 4 | 3 | - |  | 3 | - | - | - |  | - | - | - | - |  | 6 | 4 | 3 | - |
|  |  | Heart rate** | 4 | 3 | 1 | - |  | - | - | - | - |  | - | - | - | - |  | 4 | 3 | 1 | - |
|  |  | Adrenaline | 2 | - | - | - |  | - | - | - | - |  | - | - | - | - |  | 2 | - | - | - |
|  |  | Noradrenaline | 2 | - | - | - |  | - | - | - | - |  | - | - | - | - |  | 2 | - | - | - |
|  |  | Dopamine | 2 | - | - | - |  | - | - | - | - |  | - | - | - | - |  | 2 | - | - | - |
|  |  | Activity in frontal cortex of the brain | 1 | - | - | - |  | - | - | - | - |  | - | - | - | - |  | 1 | - | - | - |
|  |  | Cortisol | 8 | 1 | 4 | - |  | 2 | - | - | - |  | 1 | - | - | - |  | 11 | 1 | 4 | - |
|  |  | Hemoglobin concentration in the prefrontal area of the brain | 1 | - | - | - |  | - | - | - | - |  | - | - | - | - |  | 1 | - | - | - |
|  |  | Salivary amylase | - | - | 4 | - |  | - | - | - | - |  | - | - | - | - |  | - | - | 4 | - |
|  |  | Serum cortisol awakening response | - | 1 | - | - |  | - | - | - | - |  | - | - | - | - |  | - | 1 | - | - |
|  | Cognitive indicators | | 9 | - | 5 | 1 |  | 5 | 1 | 1 | - |  | 7 | 1 | 6 | - |  | 21 | 2 | 12 | 1 |
|  |  | Cognitive performance | 6 | - | - | - |  | 1 | - | - | - |  | - | - | 1 | - |  | 7 | - | 1 | - |
|  |  | Creativity | 1 | - | - | - |  | - | - | - | - |  | - | - | - | - |  | 1 | - | - | - |
|  |  | Attention capacity | 1 | - | 1 | 1 |  | 1 | 1 | 1 | - |  | 1 | - | 2 | - |  | 3 | 1 | 4 | 1 |
|  |  | Concentration | - | - | 1 | - |  | - | - | - | - |  | - | - | - | - |  | - | - | 1 | - |
|  |  | Inhibitory/regulative control | - | - | 1 | - |  | - | - | - | - |  | - | - | - | - |  | - | - | 1 | - |
|  |  | Working memory | 1 |  | 2 | - |  | - | - | - | - |  | - | - | - | - |  | 1 | - | 2 | - |
|  |  | Short term memory | - | - | - | - |  | 1 | - | - | - |  | - | - | - | - |  | 1 | - | - | - |
|  |  | Goal setting | - | - | - | - |  | 1 | - | - | - |  | 1 | 1 | 1 | - |  | 2 | 1 | 1 | - |
|  |  | Problem solving | - | - | - | - |  | - | - | - | - |  | 2 | - | 2 | - |  | 2 | - | 2 | - |
|  |  | Academic performance | - | - | - | - |  | - | - | - | - |  | 3 | - | - | - |  | 3 | - | - | - |
|  |  | *School achievement (e.g., academic performance)** | - | - | - | - |  | 1 | - | - | - |  | - | - | - | - |  | 1 | - | - | - |
|  | Psychosocial indicators | | 3 | - | 3 | - |  | 14 | 2 | 11 | - |  | 24 | 4 | 6 | - |  | 41 | 6 | 2- | - |
|  |  | Identity formation/ sense of identity | - | - | - | - |  | 1 | - | - | - |  | - | 1 | - | - |  | 1 | 1 | - | - |
|  |  | Confidence | - | - | - | - |  | 1 | - | - | - |  | - | - | - | - |  | 1 | - | - | - |
|  |  | Autonomy | - | - | - | - |  | 1 | - | - | - |  | 1 | - | - | - |  | 2 | - | - | - |
|  |  | Locus of control/empowerment | - | - | - | - |  | - | - | 2 | - |  | - | - | - | - |  | - | - | 2 | - |
|  |  | Self-compassion | - | - | - | - |  | 1 | - | - | - |  | - | - | - | - |  | 1 | - | - | - |
|  |  | Self-efficacy | - | - | - | - |  | 2 | - | 1 | - |  | 7 | - | 3 | - |  | 9 | - | 4 | - |
|  |  | Self-esteem | 3 | - | 2 | - |  | 4 | 2 | 2 | - |  | 9 | 1 | 1 | - |  | 16 | 3 | 5 | - |
|  |  | Self-control/-regulation | - | - | - | - |  | 1 | - | - | - |  | - | 1 | - | - |  | 1 | 1 | - | - |
|  |  | Life effectiveness | - | - | - | - |  | - | - | - | - |  | 1 | - | - | - |  | 1 | - | - | - |
|  |  | Resilience | - | - | - | - |  | - | - | 1 | - |  | 4 | - | 1 | - |  | 4 | - | 2 | - |
|  |  | Self-concept/self-perception | - | - | 1 | - |  | 1 | - | 3 | - |  | 1 | 1 | 1 | - |  | 2 | 1 | 5 | - |
|  |  | Body image | - | - | - | - |  | 1 | - | - | - |  | - | - | - | - |  | 1 | - | - | - |
|  |  | Self-pity | - | - | - | - |  | - | - | 1 | - |  | - | - | - | - |  | - | - | 1 | - |
|  |  | Daily functioning | - | - | - | - |  | - | - | 1 | - |  | - | - | - | - |  | - | - | 1 | - |
|  |  | Growth mindset | - | - | - | - |  | - | - | - | - |  | 1 | - | - | - |  | 1 | - | - | - |
|  |  | *Self-concept (e.g., self-efficacy and self-control)** | | | |  |  | 1 | - | - | - |  | - | - | - | - |  | 1 | - | - | - |
| **Physical health** | | | **6** | **10** | **7** | **-** |  | **12** | **4** | **10** | **-** |  | **1** | **-** | **1** | **-** |  | **19** | **14** | **18** | **-** |
|  | Cardiovascular indicators | | 5 | 7 | 5 | - |  | 3 | 3 | 1 | - |  | - | - | - | - |  | 8 | 10 | 6 | - |
|  |  | Systolic and diastolic blood pressure | 3 | 7 | 3 | - |  | 2 | 2 | 1 | - |  | - | - | - | - |  | 5 | 9 | 4 | - |
|  |  | Cardiovascular disease risk biomarkers | 2 | - | 2 | - |  | 1 | 1 |  | - |  | - | - | - | - |  | 3 | 1 | 2 | - |
|  | Immune function | | 1 | 3 | 1 | - |  | 2 | 1 | 1 | - |  | - | - | - | - |  | 3 | 4 | 2 | - |
|  |  | Oxidative stress | - | 1 | - | - |  | 1 | - | - | - |  | - | - | - | - |  | 1 | 1 | - | - |
|  |  | Pro‐inflammatory cytokines | 1 | - | - | - |  | - | 1 | 1 | - |  | - | - | - | - |  | 1 | 1 | 1 | - |
|  |  | Leukocyte subsets | - | 2 | 1 | - |  | 1 | - | - | - |  | - | - | - | - |  | 1 | 2 | 1 | - |
|  | Body composition and function | | - | - | 1 | - |  | 4 | - | 6 | - |  | 1 | - | 1 | - |  | 5 | - | 8 | - |
|  |  | BMI | - | - | 1 | - |  | - | - | 3 | - |  | - | - | - | - |  | - | - | 4 | - |
|  |  | Motor skills | - | - | - | - |  | - | - | - | - |  | 1 | - | - | - |  | 1 | - | - | - |
|  |  | Walking rehabilitation | - | - | - | - |  | 1 | - | - | - |  | - | - | - | - |  | 1 | - | - | - |
|  |  | Pulmonary function | - | - | - | - |  | 1 | - | - | - |  | - | - | - | - |  | 1 | - | - | - |
|  |  | Waist circumference | - | - | - | - |  | - | - | 1 | - |  | - | - | - | - |  | - | - | 1 | - |
|  |  | Muscle strength | - | - | - | - |  | - | - | 1 | - |  | - | - | - | - |  | - | - | 1 | - |
|  |  | Perceived pain | - | - | - | - |  | 1 | - | - | - |  | - | - | - | - |  | 1 | - | - | - |
|  |  | Sick leave | - | - | - | - |  | - | - | 1 | - |  | - | - | - | - |  | - | - | 1 | - |
|  |  | Physical fitness | - | - | - | - |  | - | - | - | - |  | - | - | 1 | - |  | - | - | 1 | - |
|  |  | *Bodily function and physical health (e.g., changes in weight)** | - | - | - | - |  | 1 | - | - | - |  | - | - | - | - |  | 1 | - | - | - |
|  | Active behaviors | | - | - | - | - |  | 3 | - | 2 | - |  | - | - | - | - |  | 3 | - | 2 | - |
|  |  | Percieved barriers to physical activity | - | - | - | - |  | - | - | 1 | - |  | - | - | - | - |  | - | - | 1 | - |
|  |  | Physical activity | - | - | - | - |  | 1 | - | 1 | - |  | - | - | - | - |  | 1 | - | 1 | - |
|  |  | Variety of physical activity | - | - | - | - |  | 1 | - | - | - |  | - | - | - | - |  | 1 | - | - | - |
|  |  | Sedentarism | - | - | - | - |  | 1 | - | - | - |  | - | - | - | - |  | 1 | - | - | - |
| **Social health** | | | **1** | **-** | **-** | **-** |  | **15** | **-** | **6** | **-** |  | **12** | **3** | **4** | **-** |  | **28** | **3** | **10** | **-** |
|  | Supportive environments | | - | - | - | - |  | 4 | - | 2 | - |  | - | 1 | - | - |  | 4 | 1 | 2 | - |
|  |  | Family function | - | - | - | - |  | 1 | - | - | - |  | - | - | - | - |  | 1 | - | - | - |
|  |  | Social support | - | - | - | - |  | 1 | - | 1 | - |  | - | 1 | - | - |  | 1 | 1 | 1 | - |
|  |  | Alienation | - | - | - | - |  | 1 | - | - | - |  | - | - | - | - |  | 1 | - | - | - |
|  |  | Sense of belonging/social connectedness | - | - | - | - |  | 1 | - | 1 | - |  | - | - | - | - |  | 1 | - | 1 | - |
|  | Behaviors | | 1 | - | - | - |  | 3 | - | 1 | - |  | 1 | - | 1 | - |  | 5 | - | 2 | - |
|  |  | Helping/prosocial behaviors | 1 | - | - | - |  | - | - | - | - |  | - | - | - | - |  | 1 | - | - | - |
|  |  | Antisocial behaviors | - | - | - | - |  | 1 | - | - | - |  | - | - | - | - |  | 1 | - | - | - |
|  |  | Social avoidance | - | - | - | - |  | - | - | 1 | - |  | - | - | - | - |  | - | - | 1 | - |
|  |  | Bullying | - | - | - | - |  | - | - | - | - |  | - | - | 1 | - |  | - | - | 1 | - |
|  |  | Socioprofessional status/school attendance | - | - | - | - |  | 1 | - | - | - |  | 1 | - | - | - |  | 2 | - | - | - |
|  |  | *Risky behaviors (e.g., recidivism and substance abuse)** | - | - | - | - |  | 1 | - | - | - |  | - | - | - | - |  | 1 | - | - | - |
|  | Skills and relationships | | - | - | - | - |  | 8 | - | 3 | - |  | 11 | 2 | 3 | - |  | 19 | 2 | 6 | - |
|  |  | Social cognition/competence | - | - | - | - |  | 1 | - | - | - |  | 2 | 1 | 1 | - |  | 3 | 1 | 1 | - |
|  |  | Cooperation skills | - | - | - | - |  | - | - | - | - |  | 5 | - | - | - |  | 5 | - | - | - |
|  |  | Social functioning | - | - | - | - |  | 1 | - | - | - |  | - | - | - | - |  | 1 | - | - | - |
|  |  | Amount of conflict/conflict resolution skills | - | - | - | - |  | 1 | - | - | - |  | 1 | - | 1 | - |  | 2 | - | 1 | - |
|  |  | Peer rejection | - | - | - | - |  | - | - | 2 | - |  | - | - | - | - |  | - | - | 2 | - |
|  |  | Friendships/peer relations | - | - | - | - |  | - | - | - | - |  | 1 | - | 1 | - |  | 1 | - | 1 | - |
|  |  | Gender-based prejudice/role-conformity | - | - | - | - |  | - | - | 1 | - |  | 1 | - | - | - |  | 1 | - | 1 | - |
|  |  | Interpersonal relationships/problems | - | - | - | - |  | 2 | - | - | - |  | - | 1 | - | - |  | 2 | 1 | - | - |
|  |  | Knowledge about bullying | - | - | - | - |  |  | - | - | - |  | 1 | - | - | - |  | 1 | - | - | - |
|  |  | *Interpersonal competencies**** | - | - | - | - |  | 1 | - | - | - |  | - | - | - | - |  | 1 | - | - | - |
|  |  | *Family development (e.g., parent-child relationship)** | - | - | - | - |  | 1 | - | - | - |  | - | - | - | - |  | 1 | - | - | - |
|  |  | *Social development (e.g., alienation and social skills)** | - | - | - | - |  | 1 | - | - | - |  | - | - | - | - |  | 1 | - | - | - |
| **Total *n*** | | | **64** | **32** | **38** | **1** |  | **77** | **9** | **44** | **1** |  | **5-** | **8** | **17** | **1** |  | **191** | **49** | **99** | **3** |

+: Intervention had significant positive effect on outcome (raw count), -: Intervention had significant negative effect on outcome (raw count), +/: Intervention had significant positive or nonsignificant effect on subsets of outcome (raw count), /: Findings were non-significant (raw count), % p: Percentage of total that had positive effect on outcome, % p+m: Percentage of total that had positive or mixed effect on outcome.

* Results are based on aggregate measure from the meta-analysis by Bowen and Neill (81)

** Heart rate is sometimes used as an indicator of stress-related phenomena. However, heart rate is subject to a multitude of physiologic cardiac functions and therefore not an interpretationally robust measure of stress.

*** Findings are based on meta-analysis based on five controlled by Bedard (79).
